# Force generation in human blood platelets by filamentous actomyosin structures

**DOI:** 10.1016/j.bpj.2023.07.010

**Published:** 2023-07-20

**Authors:** Anna Zelená, Johannes Blumberg, Dimitri Probst, Rūta Gerasimaitė, Gražvydas Lukinavičius, Ulrich S. Schwarz, Sarah Köster

**Affiliations:** 1 Institute for X-Ray Physics, University of Göttingen, Göttingen, Germany; 2 Institute for Theoretical Physics, University of Heidelberg, Heidelberg, Germany; 3 BioQuant-Center for Quantitative Biology, University of Heidelberg, Heidelberg, Germany; 4 Max Planck Institute for Multidisciplinary Sciences, Göttingen, Germany; 5 German Center for Cardiovascular Research (DZHK), Partner Site Göttingen, Göttingen, Germany; 6 Cluster of Excellence “Multiscale Bioimaging: from Molecular Machines to Networks of Excitable Cells” (MBExC), University of Göttingen, Göttingen, Germany

## Abstract

Blood platelets are central elements of the blood clotting response after wounding. Upon vessel damage, they bind to the surrounding matrix and contract the forming thrombus, thus helping to restore normal blood circulation. The hemostatic function of platelets is directly connected to their mechanics and cytoskeletal organization. The reorganization of the platelet cytoskeleton during spreading occurs within minutes and leads to the formation of contractile actomyosin bundles, but it is not known if there is a direct correlation between the emerging actin structures and the force field that is exerted to the environment. In this study, we combine fluorescence imaging of the actin structures with simultaneous traction force measurements in a time-resolved manner. In addition, we image the final states with superresolution microscopy. We find that both the force fields and the cell shapes have clear geometrical patterns defined by stress fibers. Force generation is localized in a few hotspots, which appear early during spreading, and, in the mature state, anchor stress fibers in focal adhesions. Moreover, we show that, for a gel stiffness in the physiological range, force generation is a very robust mechanism and we observe no systematic dependence on the amount of added thrombin in solution or fibrinogen coverage on the substrate, suggesting that force generation after platelet activation is a threshold phenomenon that ensures reliable thrombus contraction in diverse environments.

## Significance

Blood clotting is an essential physiological process and requires precise control: decreased and increased clotting both can have fatal consequences. The major cellular players in blood clotting are platelets. Upon activation these tiny cell fragments spread out at the site of injury, contract, and eventually form the blood clot, a cellular aggregate held together by a fibrinogen matrix. Combining live-cell imaging, traction force microscopy, and superresolution imaging, we demonstrate that cytoskeletal structure formation and force generation are strongly correlated. Force is localized in focal adhesions at the endpoints of a few actomyosin bundles arranged in simple geometrical patterns and the resulting force is not systematically dependent on thrombin concentration and fibrinogen coverage, suggesting a very robust mechanism of force generation.

## Introduction

Blood platelets are essential for blood clotting and primary hemostasis, and thus play an important role in wound healing. Platelets are non-nucleated fragments of megakaryocytes (1) with a large variability between the individual cells. They do not contain any microtubule organizing center (2), but the cytoskeletal filaments F-actin, microtubules (1), desmin, and vimentin intermediate filaments (3), as well as different types of molecular motors, including dynein, which slides microtubules (4), and myosin, which contracts actin filaments (5). In the resting state, the platelets circulate in the blood stream as small discoids, shaped by the so-called marginal band consisting of a microtubule coil (1). The activation of platelets can be triggered by various reagents such as adenosine diphosphate (ADP), thrombin, and serotonin (6) but also by mechanical shear (7). The most common activator of platelets is thrombin, which is also responsible for stabilization of the blood clot by enzymatic conversion of fibrinogen into fibrous fibrin (1,8,9,10). Once the platelets are activated, the marginal band first expands due to motor activity and then coils under the opposing force of the actomyosin cortex (4,11,12,13). Therefore, platelets show a spherical shape after activation. At the same time, they activate integrin-based adhesion to matrix proteins. Therefore, on a flat substrate, they strongly adhere and show a very flat and thin organization (14). Platelets first form filopodia, then thin lamelopodia (15,16,17,18,19,20), and finally contractile actomyosin bundles, which, in contrast to other adherent cell types, tend to be excluded from the cell center that typically is filled with granules and the coiled microtubules resulting from the marginal band (21,22).

In summary, actomyosin is essential for force generation and platelet contraction. To gain a better understanding of the underlying mechanisms, direct—ideally dynamic—imaging of actin structures is of great importance. However, due to the lack of a nucleus, transfections are not possible and most experiments rely on staining of actin structures in fixed platelets by phalloidin. Using superresolution fluorescence microscopy in combination with electron microscopy, actin structures have been imaged at high resolution (23). The overall shapes of actin structures in platelets have been categorized and characterized by morphometric analysis, depending on different protein coatings and different substrate stiffnesses (23). Notably, platelets from mice expressing LifeAct-GFP have been employed as well (22,24). These experiments revealed that platelets spread via actin-dependent structures, i.e., filopodia, which are then filled by lamellipodia. The recently introduced silicon-rhodamine (SiR) actin probe (25) allows for life staining of actin in human platelets as well, and first experiments revealed a time constant of 245 s ± 144 s for the formation of the complete actin cytoskeleton, compared to a faster spreading of the platelet membrane with a time constant of 68 s ± 34 s (26).

Complementing imaging studies and motivated by the central role of force generation by platelets, force measurements have been performed by, e.g., bulk rheology measurements (27) and on so-called microthrombi on elastic micro-pillars (28). To distinguish effects by the platelets from influences of the matrix that connects them, single-cell measurements have been performed. Atomic force microscopy (AFM) reveals an average contractile force of 19 ± 3.1 nN (29). A similar force range, albeit slightly higher, was obtained from platelet contraction cytometry, based on the displacement of fibrinogen-coated microdot pairs on hydrogels (30). The forces are in the range of 20–50 nN. By staining fixed platelets, the authors conclude that actin is involved in platelet force generation.

Traction force microscopy (TFM), rather than measuring total forces only, allows for computing spatio-temporally resolved force fields. Experiments with soft gels (4 kPa) have shown contractile forces in the range of 34 nN 25 min after platelet attachment to the gels (31). Further development of the experimental setup and data analysis procedures has shown that forces produced by platelets can reach an averaged value of 200 nN in the range of 30 min. Moreover, the increased spatial resolution has allowed us to detect so-called force hotspots of the traction forces at the periphery of the platelets (32). Thus, the force levels obtained by TFM are about an order of magnitude higher than values from experiments with other methods, possibly because the platelets are very flat and exert their force in a more distributed manner on flat substrates.

The imaging experiments of actin structures in platelets along with force measurements suggest that the actomyosin system is responsible for platelet spreading, shape dynamics, and contraction. However, a direct combination of imaging and force measurements has not been achieved before. Therefore, here, we combine time-resolved TFM with simultaneous live imaging of actin to quantify and correlate actin structures and force fields in a time-resolved manner. Interestingly, the force hotspots that have been observed before are spatially and temporally correlated with the endpoints of the actomyosin fiber structures. We confirm these findings by superresolution STED microscopy of actin and vinculin at the endpoint of the spreading process. We thus provide the first direct proof of force generation by fibrous actomyosin structures in platelets. For a gel stiffness in the physiological range, we do not observe a systematic dependence of force generation on the thrombin concentration in solution or the fibrinogen coverage of the substrate.

## Materials and methods

### Platelet purification and live-cell actin staining

The experiments are performed in accordance with the Ethics Committee of the University Medical Center Göttingen, votum 11/11/09. Platelet concentrates are obtained from healthy donors. Platelets are purified as previously described (31,32,33). In brief, 4 mL of the platelet concentrate is transferred into a tube together with prostaglandin E1 (PGE1 2.6 *μ*g/mL, Cayman Chemical Company, Ann Harbor, MI, USA). The content is mixed and centrifuged for 20 min at 480 × g and 21°C. The supernatant is replaced by PIPES saline glucose buffer (PSG; i.e., 5 mM PIPES, 145 mM NaCl, 5 mM glucose, 4 mM KCl, 1 mM MgCl_2_, 0.05 mM Na_2_ HPO_4_, pH 6.8) with PGE1. The centrifugation step is repeated twice. After the last removal of the supernatant, the pellet is resuspended in HEPES-Tyrode buffer (HT; i.e., 134 mM NaCl, 12 mM NaHCO_3_, 2.9 mM KCl, 1 mM MgCl_2_, 5 mM HEPES, 5 mM glucose, 0.34 mM NaH_2_ PO_4_, pH 7.4) with bovine serum albumin (BSA; 5 mg/mL, Macs BSA stock solution, Milteny Biotech, Bergisch Gladbach, Germany). The platelets are counted using a hematocrit capillary (sodium heparinized 75 mm/75 *μ*L, Hirschmann Laborgeräte, Eberstadt, Germany) and diluted to a final concentration of 2 × 10^7^ cells/mL. After purification and until the start of the experiments, the platelet suspension is kept on a cell rotor (MACSmix Tube Rotator, Miltenyi Biotech, Bergisch, Gladbach, Germany). The cell suspension is then used within 4 h. SiR-actin (final concentration 0.5 *μ*M, exitation/emission: 652/674 nm, Spirochrome, Stein am Rhein, Switzerland) is added to the diluted platelet suspension and incubated for 15 min before the samples are used in the experiments.

### Fabrication of polyacrylamide substrates

For the live-cell experiments, the protocol for gel preparation is adapted from (34). Square glass coverslips (24 mm × 24 mm no. 1, VWR, Radnor, PA, USA) are cleaned with isopropanol and dried under a nitrogen stream. Subsequently, both sides of the glass coverslips are exposed to an air plasma (0.5 mbar, ZEPTO, Diener Electronics, Ebhausen, Germany) for 1.5 min at 50 W. Next, the coverslips are soaked in a silanization solution consisting of 2% (v/v) 3-(trimethoxysilyl)propyl methacrylate (Sigma-Aldrich, St. Louis, MO, USA) and 1% (v/v) acetic acid (100%, Carl-Roth, Karlsruhe, Germany) in absolute ethanol (≥99.8%, Carl-Roth) for 10 min. The excess of the solution is aspirated and the coverslips are rinsed with ethanol. Air-dried silanized coverslips are baked at 120°C for 1 h and stored at room temperature (RT).

For the experiments employing different thrombin concentrations, circular glass coverslips (Ø 18 mm no. 1, VWR) are cleaned with isopropanol, dried under a nitrogen stream, coated with PlusOne Repel-Silane (GE Healthcare, Little Chalfont, UK) from both sides for 5 min, and washed with ethanol. A polyacrylamide (PAA) mixture for a final gel stiffness of 34 kPa is prepared by mixing 2.5 mL of acrylamide (40%, Bio-Rad Laboratories, Hercules, CA, USA), 0.75 mL of bis-acrylamide (2%, Bio-Rad Laboratories), and 6.75 mL of phosphate-buffered saline (1× PBS; i.e., 137 mM NaCl, 2.7 mM KCl, 10 mM Na_2_ HPO_4_, 1.8 mM kH_2_ PO_4_, pH 7.2). This stiffness corresponds to healthy femoral artery tissue (35). Then 10 *μ*L of carboxylated beads (0.2% solids, FluoSpheres, carboxylate-modified microspheres, 40 nm in diameter, yellow-green, 505/515 nm, Thermo Fisher Scientific, Waltham, MA, USA) are added to 490 *μ*L of the PAA solution. The addition of 10 *μ*L ammonium persulfate (10% APS, Bio-Rad Laboratories) and 0.5 *μ*L of TEMED (N,N,N′,N′-tetramethylethane-1,2-diamine, Bio-Rad Laboratories) initiates the polymerization. The PAA solution is mixed and 10 *μ*L are pipetted on a circular, PlusOne Repel-Silane-treated coverslip and covered by a squared silanized coverslip. The gel is polymerized upside down for 1 h. After the polymerization, the gel is soaked in PBS and the circular glass coverslip is removed. The top layer of the gel is coated twice with Sulfo-SANPAH (0.4 mM in 50 mM HEPES buffer (pH 8), Thermo Fisher Scientific) by applying UV light for 8 min (365 nm 2 tube 8 W, HerolabLaborgeräte, Wiesloch, Germany). Subsequently, 150 *μ*L of fibrinogen solution (100 *μ*g/mL, CalBiochem-Merck, Darmstadt, Germany) is added on top of the gel and incubated overnight at 5°C to achieve uniform coverage. Coated gels are washed with PBS and kept submerged in PBS until used in the experiments.

For testing different degrees of fibrinogen coating, we use micro-patterned substrates. To do so, we employ light-induced molecular adsorption of proteins (LIMAP) using a PRIMO setup (Alveóle, Paris, France) installed on an inverted fluorescence microscope (IX83, Olympus, Hamburg, Germany) equipped with a 20× objective (LUCPLFLN,/0.45 N.A. air, Olympus) and controlled via the software Leonardo (Alveóle). Circular glass coverslips (Ø 20 mm no. 1, VWR) are washed with isopropanol, dried under a nitrogen stream, and plasma treated for 3 min at 50 W. A polydimethylsiloxane (PDMS) stencil with a well of Ø 3.5 mm is placed on top of the plasma-treated coverslip. The glass coverslip area inside the well is passivated using 10 *μ*L of PLL-g-PEG (0.1 mg/mL in PBS (PLL(20)-g[3.5]-PEG (2 kDa)), SuSoS, Dübendorf, Switzerland) and incubated for 1 h, followed by washing three times with PBS. After removing the PBS, the empty well is filled with 5 *μ*L of photo-initiator (Product of Liaison for Protein Patterning (PLPP), Alveóle). The PLPP degrades the passivated layer of PLL-g-PEG after exposure to UV light to a degree that is linearly dependent on the dose (36). The maximum dose we use for printing a pattern is 2000 mJ/mm^2^ corresponding to 100% fibrinogen coverage. According the linear dependence between dose and coverage, we also prepare samples with 25% and 10% of the dose. After exposure to the UV light, the glass area inside the well is washed three times with PBS and subsequently filled by 10 *μ*L of fibrinogen solution (100 *μ*g/mL, CalBiochem-Merck). The protein is incubated inside of the well for 2 h at 5°C and washed three times with PBS. Successful patterning and transfer to the gel is proved by using fluorescently labeled fibrinogen (100 *μ*g/mL, Alexa Fluor 488 conjugate, excitation/emission: 495/519 nm, Invitrogen, Darmstadt, Germany; see supporting material, Figs. S1 and S2). To transfer the fibrinogen patterns from the glass coverslips to PAA gels, the PDMS stencil is removed and the glass coverslip is dried by blotting with tissues. A PAA mixture is prepared as described above (note that in this case the fluorescent beads are passivated with PLL-g-PEG (0.1 mg/mL in PBS)), pipetted to the top of the circular, patterned coverslip, and covered by a squared, silanized coverslip. After 1 h, the gels are soaked in PBS and the top glasses with the pattern are gently removed. The surface of the gel now presents the previously produced fibrinogen pattern.

For experiments with confocal and stimulated emission depletion (STED) microscopy, the PAA gels are produced similarly to the protocol described above. We use round glass coverslips with a 50 *μ*m grid (Ø 28 mm no. 1.5 Schott glass, Ibidi, Martinsried, Germany) to locate of cells after fixation. A clean coverslip is exposed to air plasma (0.5 mbar) for 30 s at 40 W, followed by a 5-min incubation with 3-aminopropyltrimethoxysilane (APTMS; Sigma-Aldrich). Subsequently, the coverslip is rinsed with ultrapure water, dried, and treated with 0.5% glutaraldehyde (70% EM grade, Polysciences, Warrington, PA, USA) in PBS, and incubated for 30 min at RT. After incubation, the coverslip is washed with ultrapure water and dried. The dried coverslips are used for the fabrication of PAA gels as described above. The polymerized gels are coated with Sulfo-SANPAH and incubated with fibrinogen (100 *μ*g/mL).

### Live-cell TFM and epi-fluorescence imaging

**Figure 1:**
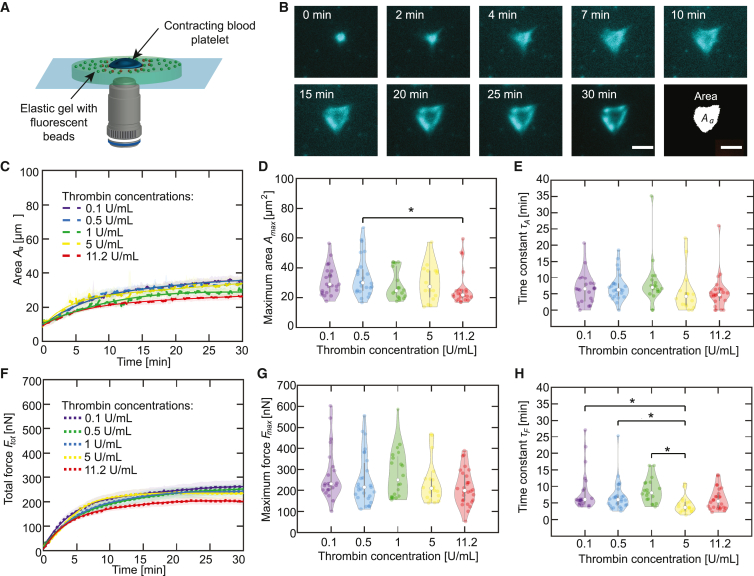
Above a threshold, thrombin concentration has no systematic effect on platelet spreading and force generation. (*A*) Schematic representation of the experimental setup. A contracting platelet (blue) on top an elastic gel (light green) displaces the fluorescent beads (green). The deformation of the gel due to the contraction is visualized as orange beads. (*B*) Epifluorescence micrographs of a blood platelet stained for actin and spreading on an elastic gel after stimulation by thrombin. The last image shows a binarized version of the t=30 min micrograph. The scale bars correspond to 5 *μ*m and refer to all sub-panels. The example platelet shown here is also shown in Figs. 3, 4*A*, and 5. (*C*) The average time-dependent total area covered by actin structures $A_{a}\left(t\right)$ upon stimulation by different concentrations of thrombin. Each averaged curve contains a minimum of 18 datasets and the SEs are shown as the transparent areas. Averaged exponential fits are shown as solid lines. (*D*) Maximum areas for each dataset determined from the averaged total $A_{a}\left(t\right)$ curves shown in (*C*). (*E*) Time constants for each dataset determined from the averaged total $A_{a}\left(t\right)$ curves shown in (*C*). (*F*) Average total force curves for blood platelets (*n*≥ 18) upon stimulation by different concentrations of thrombin are shown as dotted lines, and the SEs are included as the transparent areas. Averaged exponential fits are shown as solid lines. (*G*) Maximum forces for each dataset comprising the averaged total force curves shown in (*F*). (*H*) Time constants for each dataset comprising the averaged total force curves shown in (*F*). The violin plot outlines illustrate the kernel probability density; the width of the shaded area represents the proportion of the data points located there. In each violin plot, the white circle indicates the median, the thick gray bar indicates interquartile range, and the thin gray line indicates the full range of distribution without outliers. Experimental conditions with a statistical difference below a 5% significance level are marked by asterisks (^∗^). To see this figure in color, go online.

All TFM experiments, independent of the gel preparation procedure, are conducted on an inverted microscope (IX81, Olympus), equipped with a 60× water immersion objective (UPlanSApo, numerical aperture (NA) = 1.2, working distance (WD) = 0.28 mm, Olympus) an MT20 Xenon arc lamp (150 W, Olympus) lamp operated at 23% of the maximum intensity and a CMOS (complementary metal-oxide-semiconductor) camera (Orca Flash 4.0, Hamamatsu Photonics Deutschland, Herrsching am Ammersee, Germany). Prepared PAA gels are washed with HT buffer containing BSA and placed inside an incubation chamber (Stage Top Incubator STX, Tokai Hit, Fujinomiya, Shizuoka-ken, Japan), including a home-built sample holder optimized for 24 × 24-mm coverslips. The incubation chamber guarantees stable physiological conditions of 5% CO_2_ and 37°C throughout the experiment. All gels have a thickness of at least 50 *μ*m and we select areas with homogeneous bead coverage for the experiments. Fig. 1 *A* shows a schematic representation of the experimental setup.

Ninety microliters of SiR-actin-stained platelet suspension are pipetted on the prewarmed PAA gel. The average number of platelets on the recorded area after 2 h is 1.5 × 10^5^ cm^−2^. We analyze only platelets that do not interact with other platelets. Ten microliters of thrombin solution (thrombin from human plasma, activity 2800 U/mg (U denotes NIH-units here), Sigma-Aldrich; diluted in HT with BSA) is added directly before the recording starts to activate the platelets. The final thrombin concentration varies from 0.05 to 11.2 U/mL. The different fibrinogen coatings are investigated using a thrombin concentration of 0.1 U/mL. For the fluorescence imaging, we employ a dual-band filter (FITC/Cy5; excitation at 470 and 628 nm and emission at 537 and 694 nm (AHF Analysentechnik, Tübingen, Germany)). We record the platelet spreading for 2 h at one frame every 7.5 s with an exposure time of 50 ms each for the actin structures and the beads. We chose a total recording time of 2 h to allow different platelets to adhere and start spreading at different time points and so as to capture 30 min of spreading and contraction time for each platelet. After 30 min, the platelets are fully contracted. Some remain in this contracted state, whereas, for some, the underlying gel relaxes, possibly due to detachment of the platelet.

### Superresolution imaging

For experiments that combine live-cell TFM with confocal and STED microscopy on fixed platelets, we proceed in two steps. In a first step (see supporting material, Fig. S3 *A*) platelets are stained by 0.5 *μ*M SiR-actin and activated by adding thrombin (0.1 U/mL). We record adhering, spreading, and contracting platelets for 2 h through the gel to obtain TFM data.

In a second step, platelets are fixed and permeabilized by 0.1% (v/v) Triton-X-100 (Carl-Roth) and 4% (w/v) formaldehyde (16%, methanol free, Thermo Fisher Scientific) in cytoskeleton buffer (9.3 mM MES, 128.34 mM KCl, 2.79 mM MgCl, 1.86 mM EGTA, pH 6.1 adjusted with NaOH) with 10% sucrose (Sigma-Aldrich) for 15–20 min and rinsed three times with PBS at RT. After the fixation, the samples are incubated with NaBH_4_ (30 mM sodium borohydride, Fluka/Honeywell, Charlotte, NC, USA) in PBS for 10 min to quench autofluorescence. After washing with PBS, the samples are incubated with a blocking solution containing 0.1% (v/v) Tween20 (Carl-Roth) and 3% (w/v) BSA (Capricorn Scientific, Ebsdorfergrund, Germany) in PBS for 30 min at RT. Subsequently, the gels with the fixed platelets are incubated with a primary antibody against vinculin (dilution 1:80, monoclonal mouse anti-vinculin V9131, Sigma-Aldrich) in blocking solution for 40 to 50 min at RT. After washing with PBS containing 0.1% (v/v) Tween 20, the samples are incubated with a secondary antibody (anti-mouse) labeled with Abberior STAR 580 (1:400 2 mg/mL, Abberior, Göttingen, Germany) and additionally 0.5 *μ*M SiR-actin in blocking solution for 1 h at RT. After washing with PBS containing 0.1% (v/v) Tween 20, samples are rinsed with ultrapure water and mounted on glass coverslips (Ø 25 mm no. 1.5, VWR) with ProLong Diamond antifade Mountant (Invitrogen) and left to polymerize for 1 day at RT. These samples are imaged through the glass slide using superresolution STED microscopy (see supporting material, Fig. S3 *B*), using an Abberior STED Facility Line microscope (Abberior Instruments, Göttingen, Germany) built on a motorized inverted microscope IX83 (Olympus) equipped with an 60× silicone oil immersion objective (UPlanSApo, NA = 1.3, WD = 0.3 mm, Olympus). For this setup, the pixel size is 30 nm and the pinhole is set to 0.92 AU (Airy units with 1 AU = 1.22 λ/NA) for STED imaging. The dwell time is set to 20 *μ*s. The fluorescent probe SiR-actin is excited by 2% power of a 640-nm laser and detected in the emission range 650–755 nm; for STED, we use a 775-nm laser with 10% power; we scan each line 10 times. The fluorescent probe STAR 580 is excited by 50% power of a 561-nm laser and detected in the emission range 571–630 nm; for STED, we use a 775-nm laser with 30% power; we scan each line 10 times. The spherical correction is set to 1 to improve the image quality.

### Data analysis

For analysis of the TFM data, we employ optical flow using the Kanade-Lucas-Tomasi (KLT) algorithm with a regularization-based approach (32). Briefly, the image sequences with the recorded beads are down-sampled from 16 to 8 bit in ImageJ (37). The Shi-Tomasi corner detector tracks to find position of maximum 1000 beads. To avoid double counting of clusters, a minimum distance between beads is set to 3 pixels. The pyramidal KLT algorithm is used to track the intensity gradients around the detected beads within search windows of size 48 × 48 pixels and in the second round of size 24 × 24 pixels. The displacements are tracked between successive images and the complete trace is reconstructed with reference to the undeformed image before adhesion of the platelet. We assume a linear movement inside each of the search windows using a forward Euler approach. The drift correction is applied on a small area of the image sequence without deformation and the detected drift is subtracted from all calculated displacements. The drift-corrected displacements are interpolated onto a regular 4 × 4 pixels grid. Application of a Tukey filter (*α* = 0.2 for the cells shown in Figs. 3 *A* and 5 *A*, *α* = 0.4 for Fig. 6 *B*.i to v; Fig. 7 *C*.i to v and in the supporting material) ensures a zero velocity field at the border of the grid with drift-corrected displacements. The traction forces are calculated by Fourier transform traction cytometry (FTTC) with Tikhonov regularization using regularization parameters defined by a generalized cross-validation (GCV) function. The total force $F_{tot}$ is given by

(1) $$F_{tot}=\int_{A_{roi}}\left|\vec{T}\left(\vec{x}\right)\right|\;{\text{dA}}_{\text{roi},}$$

where $\vec{T}\left(\vec{x}\right)$ is the traction vector at a given position $\vec{x}$ and $A_{roi}$ defines the selected area containing a single contracting platelet.

The time point $t_{0}$ for each video is defined such that it corresponds to the starting point of contraction determined from the total force $F_{tot}\left(t\right)$: the curve is smoothed using the “rloess” function in MATLAB (38), which reduces the effect of outliers and $t_{0}$ is defined as the time point of the first maximum of the second derivative of the smoothed $F_{tot}\left(t\right)$. The area $A_{a}\left(t\right)$ covered by actin at any given time point in the image sequence corresponds to the platelet spread area. It is determined by first applying a Gaussian filter with standard deviation 1 on the image sequence to reduce the noise. The smoothing is essential in particular for the analysis of the early time points. Next, we threshold the image following Kittler’s method of minimum error thresholding (39). The method performs well in cases where the distributions of the object and background have unequal variances. The finally obtained area $A_{a}$ is cleaned of unconnected segments and plotted against time. For large peaks in the signal due to detection of inseparable clusters of floating platelets that do, however, not attach to the platelet of interest, the data are interpolated using the nearest neighboring non-outlier values. Outliers are defined as area values more than three standard deviations from the mean. A detailed description of the algorithm used here can be found in the supporting material in Fig. S4.

The resolution of the traction reconstruction is predominantly determined by the spacing between neighboring beads and the interpolation grid spacing. Assuming that the bead positions are uncorrelated, we can use a Poisson distribution to describe the local bead density. From this distribution, the average distance between neighboring beads can be estimated to be

(2) $$\left\langle r\right\rangle =\frac{1}{2}\sqrt{\frac{hw}{N_{B}}}$$

where *h* and *w* are the height and width, respectively, of the area recorded in the image, and $N_{B}$ is the number of the detected beads. For a typical image we found *h* = *w* = 25 *μ*m and $N_{B}=1000$. This results in an average bead spacing ⟨r⟩=395 nm, that can be resolved with the optical microscope and justifies the average grid spacing of 400 nm used in the regular grid interpolation.

We quantify the correlation between the intensity of the vinculin immunostaining and the magnitude of the traction forces using previously published MATLAB scripts by Lickert et al. (23). First, the script “Define Cells” is used to determine the centroid of the actin boundary. Second, the script “Process Cells” is applied to divide the vinculin images and the traction force maps into 20 equal angular sectors that originate from the centroid determined before. The intensity values in each sector are averaged and plotted against the azimuthal angle (red crosses for vinculin, blue crosses for force magnitude). These circumferential profiles are fitted to the function Y(α)=1+a·cos(α)+b·sin(α), where *α* is the azimuthal angle.

To extract the stress fibers, we use a combination of the MATLAB scripts by Lickert et al. (23) and the tool FilamentSensor (40). We again use the scripts Define Cells and Process Cells, but this time to obtain a binarized bitmap of the actin staining as well as a prediction of the local stress fiber orientation angle. We then determine the dominant orientation angle and produce two bitmap images: one in which only those bits of the actin image are retained, in which the corresponding orientation angle is roughly parallel to the dominant orientation angle (angles differ by less than 45°), and one in which only those bits of the actin image are retained in which this is not the case. We found that this procedure of processing stress fibers in these two groups improves their reconstruction. Both bitmap images are then processed independently using the FilamentSensor. We load the images using the “Stack View” option and select “As straight pieces” in the “Line Sensor” menu to obtain the filaments in a segment-wise manner. We then use the “Export filaments as .cs” option to extract the segments. In a post-processing step, we first assemble the segments into piece-wise straight lines and then merge the filament datasets obtained from the parallel and non-parallel bitmap images.

To compare the localization of vinculin to actin in the STED images, we employ a Laplacian filter on the vinculin data to identify the centers of the vinculin patches. We then use thresholding and a connected component analysis to identify the spatial extent of the patches around these points. Sizes and orientations are computed by performing a moment analysis and fitting ellipses to the results. The mathematical details are given in the supporting material.

For statistical comparison of different experimental conditions, we first apply a Kruskall-Wallis rank sum test to all conditions. In cases where the Kruskall-Wallis rank sum test shows a difference on a significance level of 5%, we additionally perform a Dunn’s test on all possible pairings, again with a 5% significance level.

## Results

### Effect of thrombin concentration on platelet spreading and force generation

To investigate the spreading speed and force evolution of platelets when activated by different thrombin concentrations between 0.05 and 11.2 U/mL, we record movies of the emerging actin structures and, in parallel, the bead patterns below the platelets, which are then used to calculate the force fields with TFM procedures. All gels are incubated with a fibrinogen solution with a concentration of 100 *μ*g/mL. At a thrombin concentration of 0.05 U/mL, we do not observe spreading, actin structure formation, or force generation (see Fig. S5 in the supporting material), in contrast to the situation at 0.1 U/mL. We thus find that the threshold thrombin concentration for platelet activation lies between 0.05 and 0.1 U/mL. Above this threshold, the actin structures in thrombin-activated platelets dramatically change upon spreading on a fibrinogen-coated gel, as shown for a typical example in Fig. 1 *B* and Video S1. Note that imaging at high magnification is challenging when the optical path encompasses a PAA gel in addition to a glass coverslip. We analyze the increase of the area occupied by actin structures $A_{a}$ with respect to time, rather than the fluorescence intensity, as it has been shown that the binding kinetics of the SiR-actin probe is slower than the actin polymerization dynamics (26). Fig. 1 *C* shows $A_{a}\left(t\right)$, color coded for the different thrombin concentrations used for platelet activation. The dashed lines denote the data, averaged from a minimum of 18 platelets each, whereas the transparent areas show the SEs. The individual data curves are shown in the supporting material in Fig. S6. For comparison, we fit the data with an exponential growth function,

(3) $$A_{a}\left(t\right)=A_{\max}\cdot \left[1-B_{a}\cdot \exp\left(-t/\tau_{A}\right)\right]\text{,}$$

where $A_{\max}$ is the maximum area, $A_{\max}\left(1-B_{a}\right)$ is the initial detected area, and $\tau_{A}$ is the time constant for the time-resolved area increase. The fit parameters $A_{\max}$ and $\tau_{A}$ are shown in Fig. 1 *D* and *E*, respectively. At a significance level of 5%, we observe a statistical difference for the maximum area $A_{\max}$ between 0.5 and 11.2 U/mL thrombin, but not between the other conditions and not for the time constant $\tau_{A}$ (Kruskall-Wallis rank sum test and Dunn’s test).

Video S1. Typical example of the development of the actin structure in a spreading platelet; 7.5 s per frame Note that the contrast for each frame has been adjusted based on the intensity histogram for better optical visualization of early time points. Scale bar corresponds to 5 *μ*m.

The total force $F_{tot}\left(t\right)$, derived from the traction forces according to Eq. 1 for each thrombin concentration is shown in Fig. 1 *F*. The averaged data are shown as dotted lines and the SEs as transparent areas. The individual data curves are shown in the supporting material in Fig. S7. For a quantitative comparison, we again fit an exponential growth function,

(4) $$F_{tot}\left(t\right)=F_{\max}\cdot \left[1-B_{f}\cdot \exp\left(-t/\tau_{F}\right)\right]\text{,}$$

where $F_{\max}$ is the maximum force, $F_{\max}\left(1-B_{f}\right)$ is the initial force, and $\tau_{F}$ is the time constant. The fit parameters $F_{\max}$ and $\tau_{F}$ are shown in Fig. 1 *G* and *H*, respectively. At a significance level of 5%, we observe a statistical difference for the time constant $\tau_{F}$ between 5 U/mL and 0.1, 0.5, and 1 U/mL, but not between the other conditions and not for the maximum force $F_{\max}$ (Kruskall-Wallis rank sum test and Dunn’s test). Thus, our data show that, despite their small size, platelets produce large forces of several hundreds of nanonewtons and that these forces are not systematically dependent of the thrombin concentration. Interestingly, the development of the force magnitude and the actin structures are on the same timescale.

### Effect of fibrinogen coverage on platelet spreading and force generation

**Figure 2:**
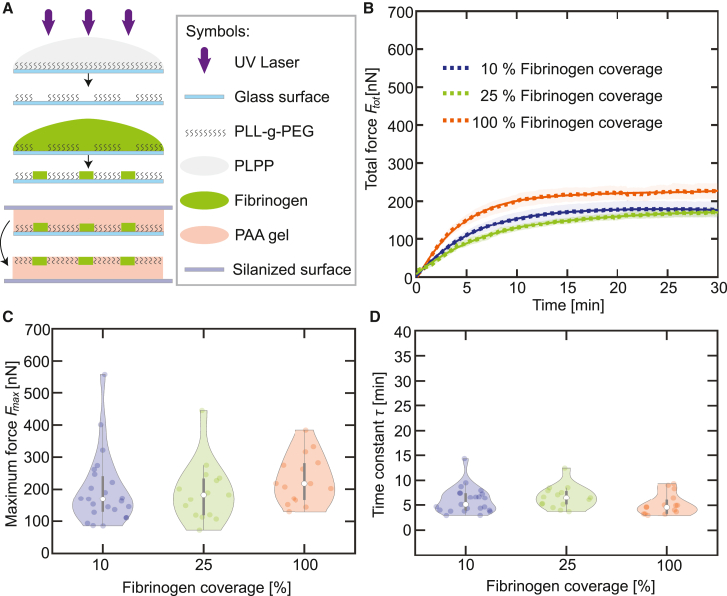
Fibrinogen coverage has no effect on force generation. (*A*) Schematic representation of the preparation of micro-patterned gel surfaces with varying fibrinogen coverage (100%, 25%, 10%) by photo patterning (LIMAP). (*B*) Average total force curves for blood platelets (*n*≥ 15) upon stimulation by thrombin (0.1 U/mL) on gels covered by various concentrations of fibrinogen (dotted lines); the SEs are included as the transparent areas. Averaged exponential fits are shown as solid lines. Each dataset comprising the averaged curves is fitted and the maximum forces and time constants are determined. (*C*) Maximum forces for each dataset determined from the averaged total force curves shown in (*B*). (*D*) Time constants for each dataset determined the averaged total force curves shown in (*B*). The violin plot outlines illustrate the kernel probability density; the width of the shaded area represents the proportion of the data points located there. In each violin plot, the white circle indicates the median, the thick gray bar indicates interquartile range, and the thin gray line indicates the full range of distribution without outliers. To see this figure in color, go online.

In a next step, we systematically investigate—similar to the experiments described above—the force evolution in dependence of the fibrinogen coverage. We use a thrombin concentration of 0.1 U/mL just above the threshold determined before and a fibrinogen solution with a concentration of 100 *μ*g/mL. The varying surface coverage (100%, 25%, 10%) is obtained by photo patterning (LIMAP) as shown in Fig. 2 *A*. We again record the substrate deformation and determine the total force $F_{tot}\left(t\right)$ for each fibrinogen coverage (see Fig. 2 *B*). The individual data curves are shown in the supporting material in Fig. S8. Each single measurement curve is fitted exponentially according to Eq. 4, and the averaged fit curves are shown as solid lines. The fit parameters $F_{\max}$ and τ are shown in Fig. 2 *C* and *D*, respectively, and they show no significant difference (Kruskall-Wallis rank sum test). Imaging the actin structures within platelets spreading on LIMAP-coated surfaces leads to high background signal and we are thus not able to determine the temporal development of the spread area. However, Fig. S9 in the supporting material shows a comparison of the contraction forces on fibrinogen-coated gels and on 100% photo patterned gels and they agree well, underlining the validity of our method. Taken together, our results show that neither the thrombin concentration in the platelet suspension nor the fibrinogen coverage on the substrate influence the force generation in a systematic manner.

### Spatial correlation of force fields and cell shape in spreading platelets

**Figure 3:**
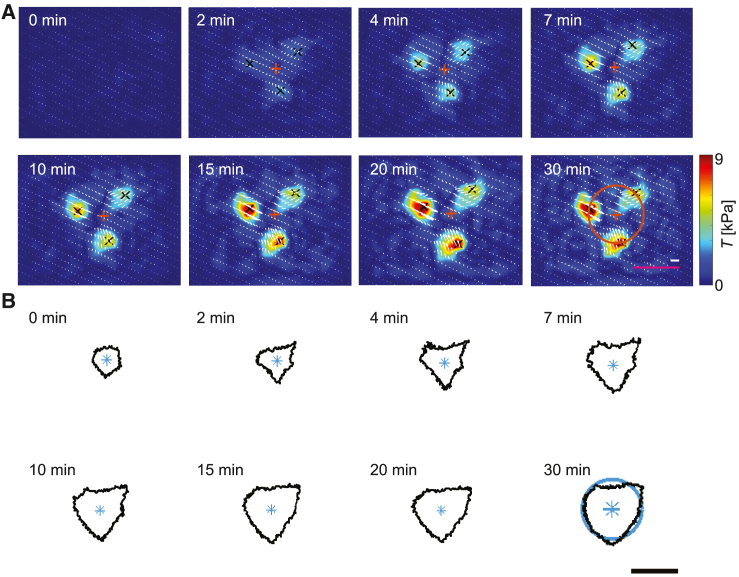
Time-resolved traction forces reveal emergence of force hotspots and show same geometry as cell shape. (*A*) Image sequence showing the temporal evolution of the force distribution. Black crosses represent the centers of the force hotspots and white arrows indicate the direction of the force field. For each image, the centroid of the hotspots is calculated and is represented as an orange cross. As an example, the orange circle shown on the t=30 min image has a radius equal to the mean radial distance of the force (${\text{MRD}}_{f}$) between the centroid and the black crosses. Corresponding ${\text{MRD}}_{f}$ are calculated for each time point of the video and each individual platelet. The magenta scale bar corresponds to 5 *μ*m, the white scale bar corresponds to 5 kPa, and they refer to all sub-panels. (*B*) Image sequence showing the time evolution of the actin boundary corresponding to the same time points shown in (*A*). For each image, the centroid is represented by a blue star. As an example, the blue circle shown in the t=30 min image has a radius equal to the mean radial distance (${\text{MRD}}_{a}$) between the centroid and the actin boundary. Corresponding ${\text{MRD}}_{a}$ are calculated for each time point of the video and each individual platelet. The scale bar corresponds to 5 *μ*m and refers to all sub-panels. The example platelet shown here is also shown in Figs. 1*B*, 4*A*, and 5. To see this figure in color, go online.

To better understand the correlation between force generation and the cell shape, in Fig. 3 *A* and *B* we show the traction fields and cell contours, respectively, for a typical time series. We observe that both processes show the same triangular geometry. The white arrows indicate the force vectors generated by the platelet. This force is mostly concentrated at the three corners of the triangular cell in force hotspots (32). We further observe that they point toward the geometrical center. We note that the internal forces are likely higher than the values we record here (see Figs. 1 *F* and 2 *B*), because TFM can only measure the vectorial sum of the forces acting through the adhesions onto the substrate. The overlay of the actin boundary and the force map is shown as Video S2, the bead patterns used to determine the force map are shown in Video S3.

Video S2. Temporal development of the traction force map, corresponding to Video S1, with the detected boundary of the actin area indicated as black lines The centroid is represented by a blue star. Magenta crosses represent the centers of the force hotspots and white arrows indicate the direction of the force field. For each image, the centroid of the hotspots is calculated and is represented as an orange cross. Scale bar corresponds to 5 *μ*m.

Video S3. Temporal development of the fluorescent bead positions used for calculation of traction force map The data correspond to Videos S1 and S2. Scale bar corresponds to 5 *μ*m.

**Figure 4:**
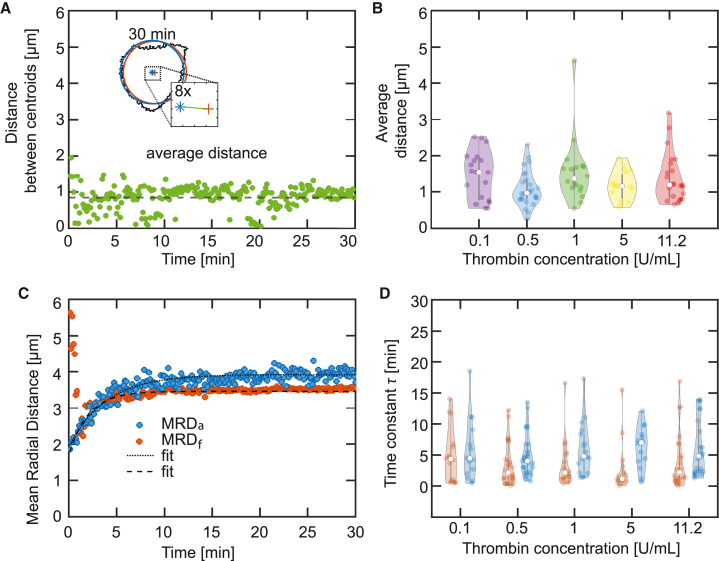
The centroids of the force hotspots and the actin boundary collapse with time. (*A*) The inset shows an overlay of the actin boundary (black), the ${\text{MRD}}_{a}$ circle (blue), and the ${\text{MRD}}_{f}$ circle (orange) for the t=30 min image. The distance between the centroids of the two circles is shown in the zoom-in and is represented by the green line. The distance between the centroids is calculated for each time point and is plotted in a time-resolved manner. The average distance is represented as the dashed black line. This analysis is repeated for every platelet used in the experiments with differing thrombin concentrations. The example platelet shown here is also shown in Figs. 1*B*, 3*A*, and 5. (*B*) Distribution of the average distance between ${\text{MRD}}_{a}$ and ${\text{MRD}}_{f}$ circles for each thrombin concentration. (*C*) Temporal evolution of the ${\text{MRD}}_{a}$ (blue) and ${\text{MRD}}_{f}$ (orange) for the example shown in Fig. 3 and (*A*). (*D*) Distribution of the time constant for the radial evolution for each ${\text{MRD}}_{a}$ circle (blue violin plots) and ${\text{MRD}}_{f}$ circle (orange violin plots). See axis labels for different conditions. The violin plot outlines illustrate the kernel probability density; the width of the shaded area represents the proportion of the data points located there. In each violin plot, the white circle indicates the median, the thick gray bar indicates interquartile range, and the thin gray line indicates the full range of distribution without outliers. To see this figure in color, go online.

To quantify the spatial and temporal correlation of the force fields and cell shape, we first determine the position of the force hotspots using the MATLAB function “Fast 2D peak finder” (41). Here, we focus on those peaks that exceed a level of 50% of the highest traction force at each time point. For each platelet, we determine the centroid of these peaks $C_{f}\left(x_{cf},y_{cf}\right)$ (see orange crosses in Fig. 3 *A*) and the mean radial distance of the force ${\text{MRD}}_{f}$, defined as the average Euclidean distance between the centroid $C_{f}$ and the detected hotspots:

(5) $${\text{MRD}}_{f}=\frac{1}{K_{f}}\cdot \sum_{k=1}^{K_{f}}\sqrt{{\left(x_{cf}-x_{f}\left(k\right)\right)}^{2}+{\left(y_{cf}-y_{f}\left(k\right)\right)}^{2}}\text{,}$$

where $K_{f}$ is number of detected peaks and $\left(x_{f},y_{f}\right)$ are coordinates of the detected peaks. Second, we determine the centroid of the actin boundary $C_{a}\left(x_{ca},y_{ca}\right)$ (see blue stars in Fig. 3 *B*) at each time point. Additionally, we introduce a variable for the mean radial distance of the actin area ${\text{MRD}}_{a}$, defined as the average Euclidean distance between the centroid $C_{a}$ and the actin boundary:

(6) $${\text{MRD}}_{a}=\frac{1}{K_{a}}\cdot \sum_{k=1}^{K_{a}}\sqrt{{\left(x_{ca}-x_{a}\left(k\right)\right)}^{2}+{\left(y_{ca}-y_{a}\left(k\right)\right)}^{2}}\text{,}$$

where $K_{a}$ is number of pixels on the actin boundary and $\left(x_{a},y_{a}\right)$ are the coordinates of the pixels. In Fig. 4 *A*, we show a typical example of the time-dependent distance between the centroid of the force hotspots $C_{f}$ and centroid of the actin boundary $C_{a}$. The averaged value is indicated by the black dashed line. For each measurement, we determine the time-averaged distance, shown in Fig. 4 *B*. The results show no significant difference (Kruskall-Wallis rank sum test, p > 0.05) between the thrombin concentrations. These results show that the cell as a whole arrives relatively fast at its final shape and an equilibrium of mutually balancing forces, which effectively are centered on the centroid. Thus, shape and force fields nicely correspond to each other.

In Fig. 4 *C* we present an example of ${\text{MRD}}_{a}\left(t\right)$ and ${\text{MRD}}_{f}\left(t\right)$ of one typical platelet. It is evident that both curves show a very similar exponential increase. We quantify this trend by fitting the curves with an exponential model:

(7) $$\text{MRD}\left(t\right)={\text{MRD}}_{\max}\cdot \left[1-B_{\text{MRD}}\cdot \exp\left(-\left(t+d\right)/\tau_{\text{MRD}}\right)\right]\text{,}$$

where ${\text{MRD}}_{\max}$ is the maximum ${\text{MRD}}_{a}$ or ${\text{MRD}}_{f}$, *d* is the time offset, and τ is the time constant. We determine the time constants as plotted in Fig. 4 *D*. In agreement with all previous results, $\tau_{{\text{MRD}}_{a}}$ and $\tau_{{\text{MRD}}_{f}}$ also show no significant difference between the measured conditions (Kruskall-Wallis rank sum test, p > 0.05). However, when comparing the medians and interquartile ranges (IQRs) of $\tau_{{\text{MRD}}_{a}}$ (*n* = 104, 5.2 ± 3.9 min) and $\tau_{{\text{MRD}}_{f}}$ (*n* = 104, 3.8 ± 3.4 min) for all conditions, we find that $\tau_{{\text{MRD}}_{f}}$ is significantly faster (Wilcoxon rank sum test, p < 0.05). Thus, the distribution of force hotspots is equilibrated early on, whereas the platelet shape, as given by the area covered by actin structures, is still developing further.

**Figure 5:**
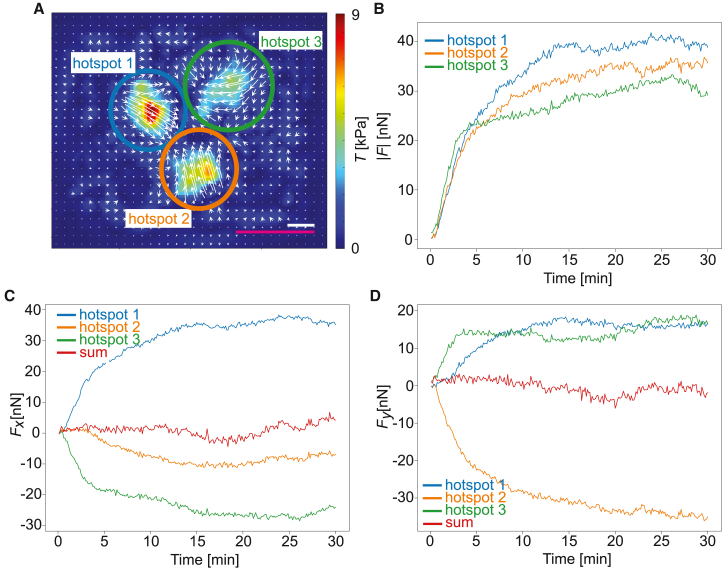
The force hotspots form a balanced system of strongly localized forces. (*A*) Reconstructed tangential traction forces at the final time point of spreading for the cell presented in Fig. 3 with three manually defined adhesion hotspot areas indicated by blue, green, and orange circles. The color coding is valid for all subfigures. The magenta scale bar corresponds to 5 *μ*m and white scale bar corresponds to 5 kPa. (*B*) Time evolution of the magnitude of the traction force vectors obtained by integrating the traction vector field in each individually marked adhesion area. (*C*) Time evolution of the *x* components of the traction force vectors for the each individually marked adhesion area. The red curve shows sum of all three components. (*D*) The time evolution of the *y* components of the traction force vectors for each individually marked adhesion area. The red curve shows sum of all three components. The example platelet shown here is also shown in Figs. 1*B*, 3, and 4*A*. To see this figure in color, go online.

In Fig. 5, we analyze the force balance of one adherent platelet. We calculate the force vectors for the three hotspots, which are all directed toward a point close to the centroid of the cell with a strength between 20 and 40 nN each at the final time point of spreading (Fig. 5 *A* and *B*). These traces confirm the time constant for the increase in contractility concluded from the evolution of total force in Fig. 2 *B*. As shown in Fig. 5 *C* and *D*, the *x* and *y* components of the three hotspot forces nicely add up to zero, thus confirming force balance and the high resolution of our measurements. Overall we conclude that this system is a geometrically simple system of strongly localized and balanced forces.

### Spatial correlation of force fields and subcellular structures in spreading platelets

**Figure 6:**
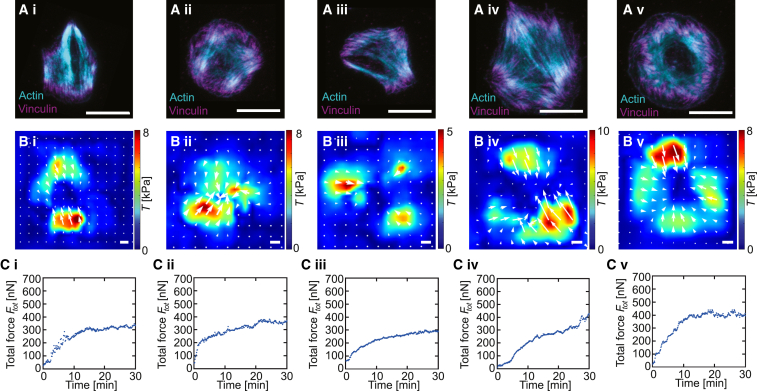
STED imaging of fixed and stained cells reveals details of cell organization during force generation. Typical examples of i) spindle-like, ii) square, iii) triangular, iv) polygonal, and v) circular platelets. (*A*) Superresolution STED imaging; actin in cyan and vinculin in magenta; the white scale bars correspond to 5 *μ*m. (*B*) Traction forces before fixation with the white arrows indicating the direction of the force fields; the white scale bars correspond to 5 kPa. (*C*) Time evolution of the total force produced by the cell before fixation. See Fig. S10 in the supporting material for five additional examples. To see this figure in color, go online.

Imaging of the cytoskeletal structures through the PAA gel is challenging and does not give the same resolution as fixed and stained samples (compare the examples in Fig. 1 *B* and Video S1). Therefore, to complement the dynamic data we record during the force measurements, we chemically fix platelets at the end of the spreading process and label actin (SiR-actin) and vinculin (immunostaining). We then embed the samples and perform superresolution STED microscopy through the glass slide on top of the platelets. In Fig. 6 *A*, we show representative examples of the images obtained by this procedure (actin in cyan and vinculin in magenta). We find that the triangular pattern shown in Figs. 3 and 5 is very common (compare Fig. 6 *A*.iii) but that other geometrical patterns also occur, namely spindles (Fig. 6 *A*.i), squares (Fig. 6 *A*.ii), polygonal (Fig. 6 *A*.iv), and circular shapes (Fig. 6 *A*.v). In Fig. 6 *B* we show the corresponding force maps recorded at the end of the spreading process, just before fixation and STED imaging, where the white arrows indicate the direction of the force field and showing areas with concentrated force hotspots at the edges of the platelets. Again, shapes and force fields show similar geometries. In Fig. 6 *C* we finally show the time courses leading to these force fields. Despite the very different cell organizations, these time courses and the plateau levels for the forces are very similar.

**Figure 7:**
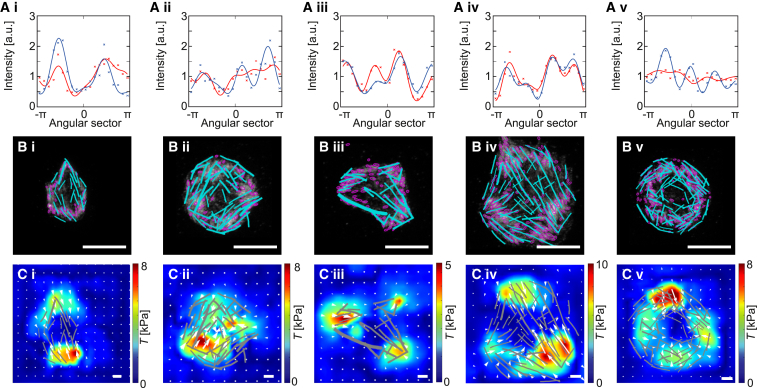
Actin structures, vinculin patterns, and force fields are correlated. The same cells (i–v) as in Fig. 6 are shown. (*A*) Circumferential profiles of the vinculin signal (red) and force magnitude (blue); the crosses show the data, the solid lines are fits. (*B*) Overlay of segmented vinculin spots (focal adhesions; magenta) and stress fibers (cyan); the white scale bars correspond to 5 *μ*m. (*C*) Overlay of the traction forces field and the segmented stress fibers (gray); the white scale bars correspond to 5 kPa. See Fig. S11 in the supporting material for five additional examples. To see this figure in color, go online.

In the STED images from Fig. 6 *A*, actin in cyan represents intracellular force generation, whereas vinculin in magenta represents adhesion and force transmission to the substrate. To contract the extracellular environment, these two systems have to work together. To analyze this aspect in quantitative detail, we turn to a pairwise correlation between vinculin intensity distribution, actin stress fiber orientation, and force fields. In Fig. 7 *A*, we show the radially integrated vinculin intensity (23) (red crosses and solid fit curves), indicative of the positions of the focal adhesions, and the radially integrated force magnitude (blue). In cells i to iv, the maxima of the curves overlay strongly, demonstrating a pronounced angular correlation of the traction forces and the focal adhesions. The circular cell (v) is an exception to this observation, as in this case, the vinculin is almost evenly distributed over all angles. In Fig. 7 *B*, we focus on the stress fibers, which are segmented (cyan) and shown together with the vinculin signal, which has been processed with thresholding and a moment analysis to indicate patch sizes and orientations (magenta ellipses). We see that the ends of the stress fibers usually correspond to vinculin patches and that their orientations tend to co-align. We note that the converse is not necessarily true, because there are also many vinculin spots that do not correspond to the endpoints of stress fibers. We also see that the circular cell is special because its stress fibers are arranged in a circular fashion and do not cross the interior of the cell; nevertheless, here too we see alignment between stress fibers and vinculin patches. Finally, Fig. 7 *C* shows that the forces at the ends of stress fibers correlate with the directions of the force field. This correlation is most obvious in the case of the spindle-shaped cell (i), when we have only one family of stress fibers. In the other cases, stress fibers of different orientations meet at one force hotspot, and then it seems to be the vectorial sum of these stress fibers that aligns with the force vectors. Again, this reflects the fact that TFM can only measure the vectorial sum of the forces transmitted through the adhesion.

Taken together, STED microscopy through glass cover slips enables us to resolve the adhesions and stress fibers in the platelets much more clearly than when using live-cell imaging through PAA gels. Notably, through the procedures established here, we are now able to combine the high resolution of STED microscopy with the dynamic traction force measurements by imaging the endpoint of these dynamics. For all 10 cells analyzed (five in Figs. 6 and 7; five more in Figs. S10 and S11 in the supporting material), we observe that traction hotspots align with the endpoints of actin stress fibers, which are anchored in focal adhesions, as shown by the vinculin immunostaining. The force arrows and the focal adhesions tend to be co-aligned, although the segmentations are not sufficient to perform a more detailed analysis. Overall, our data provide strong indication that the stress fibers ending in the focal adhesions indeed are the force-generating elements in platelets.

## Discussion

Due to the challenge of simultaneously measuring forces and imaging the cytoskeleton of the small and nucleus-free platelets, it is still not clear how exactly cytoskeletal organization and force generation correspond to each other. Here we directly connect force generation and cytoskeletal structure formation by combining our earlier advances in in situ actin imaging (26) and TFM (32). The lack of a nucleus in platelets leads us to use SiR-actin as a probe to visualize the formation of stress fibers in human platelets and we reveal similar structures as have been observed by immunostaining of chemically fixed platelets (22,23,24) as well as in time-resolved experiments with spreading platelets on stiff glass substrates (26). We confirm our new findings by superresolution STED microscopy, which reveals even more details but requires fixation. For TFM, we use state-of-the-art methods to achieve high spatial and temporal resolution, which is essential to investigate the relation between force field evolution and the temporal development of cytoskeletal structures. In agreement with our earlier work (32), we find that the maximum total force of few hundreds of nanonewtons is generated within 30 min and with the average time constants for the force increase and area increase 6.0±2.9 min and 5.5±3.6, respectively. This observation is also in a good agreement with the observed time constant for actin structure formation on glass (4.1±2.4 min) (26).

Because of the limited resolution of our live-cell imaging, in particular for the early time points, we analyze the overall substrate coverage by actin, which corresponds to the spreading area, rather than individual fibrous actin structures. The limitations in resolution are caused by the slow binding kinetics of the SiR-actin probe (25) as well as by the fact that the imaging is performed though a relatively thick PAA gel with an increasing background by unbound dyes. Our results confirm that platelets produce the largest traction forces in force hotspots (32). We are able to spatially and temporarily correlate these hotspots with the endpoints of straight actin structures, which we interpret as force-generating actin stress fibers. Consistent with our results reported here, a recent study found that at a high-level tension regime integrins are focused at two to three spots at the periphery after 4 min (42). Interestingly, we observe a significantly slower time constant (Wilcoxon rank sum test, p < 0.5) for the spatial development of actin structures compared to the time constant for the positions of the hotspots. This indicates that the hotspots are spatially stabilized before the actin structures are fully rearranged. This could indicate that the spatial force distribution is organized at early time points of the actin polymerization; at a later stage, the acto-myosin interactions increase the force and stabilize the contraction.

Our approach of combining live-cell imaging with time-resolved TFM and STED imaging of the final actin/vinculin structures provides a consistent picture of force generation in platelets. The actin stress fibers, which can be segmented well in the STED images, are anchored in focal adhesions, which we visualize by vinculin staining, and correlate in direction with the measured forces, with the caveat that, when different stress fibers are anchored to the same adhesion, their forces seem to add up. The shape of each platelet is mostly defined by these stress fibers, which lead to simple geometric shapes, such as spindles or triangles. Additional vinculin structures, and therefore adhesion points, are found in the regions between the endpoints of the stress fibers, where they might serve as anchor points for lamellipodia and other intracellular structures. When analyzing the force fields that the platelets exert on the elastic substrate, we observe hotspots, which emerge early in the spreading process, are colocalized with the vinculin staining in the focal adhesions, remain spatially stable over time, and increase in force magnitude, presumably through growth of stress fibers. Thus, over a time course of about 30 min, a total force per platelet of several hundreds of nanonewtons is reached.

Neither the total force, nor the time constant for force generation, nor the actin area or the correlation of actin structures and forces fields are systematically influenced by varying concentrations of thrombin added to the platelets suspension as long as the threshold concentration for activation by thrombin is ensured. In agreement with earlier results by Myers et al. (30) for stiffness values of 25 or 50 kPa, which are closest to our value of 34 kPa, the effect of thrombin is indeed not very pronounced and may be negligible given the platelet-to-platelet variation we encounter in our experiment. We might have obtained different results for lower stiffness, as reported earlier (30), but here we focus on a physiologically relevant stiffness value (35). Our measured total platelet forces, integrated over the total platelet spread area, are higher than the forces measured by Myers et al. (30), likely because, in that work, only the 1D force exerted on two dots is determined, whereas we take the full platelet force field into account. Because these forces are strongly localized, we also expect that platelets in physiological 3D environments exert forces that are larger and more localized than formerly appreciated. To prove this expectation, in the future one would have to develop methods for 3D-TFM that allow for spatially resolving the forces of the very small platelets, such as using 3D-printed structured environments (43).We also find that the total force is not influenced by the fibrinogen coverage on the substrate. Biologically, this could indicate that platelet contraction, and possibly function in general, is a very robust process that is not easily influenced by external parameters once activated. In the future, it would be interesting to also study other pathways to platelet activation, such as ADP or serotonin (6), and different substrate coatings, such as fibronectin and collagen. In contrast to our results, a recent study shows that an increasing thrombin concentration increases platelet forces in microthrombi (28). Hence, our results on a single platelet may indicate that the force increase in microthrombi is more likely connected to the cell-cell interaction or to the tension of fibrin fibers in the larger cluster of cells. It is not clear yet if all platelets within a microthrombus actively contribute to the force increase and how. Therefore, further research on small ensembles of platelets is needed to validate this hypothesis. An investigation of their adhesion and dynamics of the forces could provide information in this regard.

Taken together, our study demonstrates a close relation between filamentous actomyosin structures and force generation in healthy blood platelets. Our approach has the potential to better understand in the future how bleeding disorders such as Glanzmann’s thrombasthenia might arise because they disturb the relation between formation of actin structures and force generation.

## Author contributions

S.K. and U.S.S. designed research and supervised this work. A.Z. performed research. J.B. and D.P. contributed data analysis tools. R.G. and G.L. supported the superresolution experiments. J.B. and A.Z. analyzed data. A.Z. and S.K. wrote the original draft of the paper. A.Z., J.B., U.S.S., and S.K. worked on the final version. All authors reviewed and approved the paper.

## Supporting material

Document S1. Figures S1–S11

Document S2. Article plus supporting material
