# Supplementary material for: Force generation in human blood platelets by filamentous actomyosin structures
Source: Biophys J. 2023 Jul 20;122(16):3340–53. doi: 10.1016/j.bpj.2023.07.010 (PMC10465724; doi:10.1016/j.bpj.2023.07.010)
Supplement: Document S1. Figures S1–S11 [file mmc1.pdf]

Biophysical Journal, Volume *122*

Supplemental information

Force generation in human blood platelets by filamentous actomyosin structures

Anna Zelená, Johannes Blumberg, Dimitri Probst, Rūta Gerasimaitė, Gražvydas Lukinavičius, Ulrich S. Schwarz, and Sarah Köster

Supplementary figures

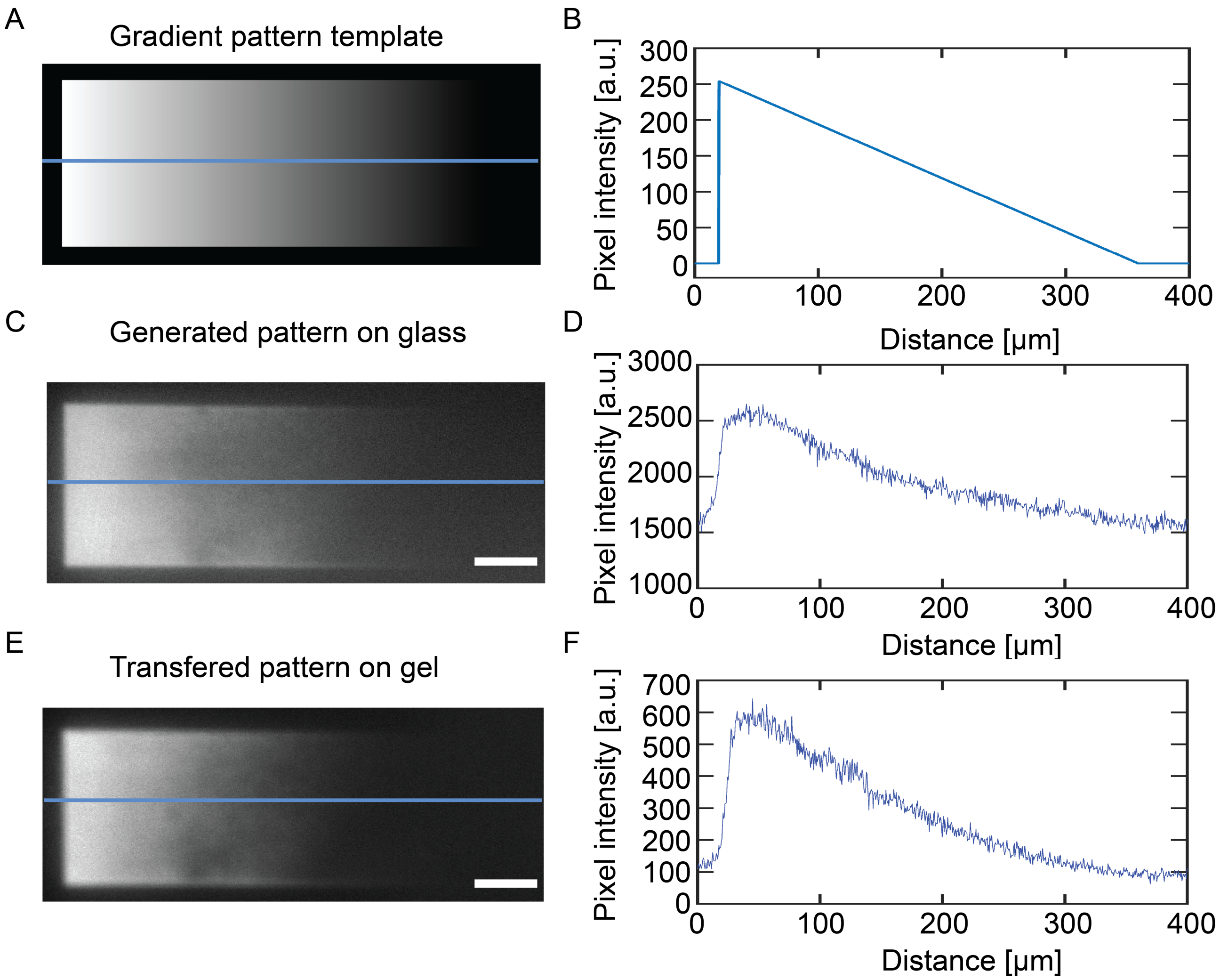

Fig. S1 (A) Template pattern in gray scale. The blue line marks the line scan shown in (B). (C, D) Corresponding gradient pattern generated with LIMAP on glass after incubation with 100 μg/mL fluorescently labeled fibrinogen using a laser power of 2000 mJ/mm^2. (E, F) Corresponding gradient pattern obtained after transfer to the PAA gel. Scale bars correspond to 50 μm.

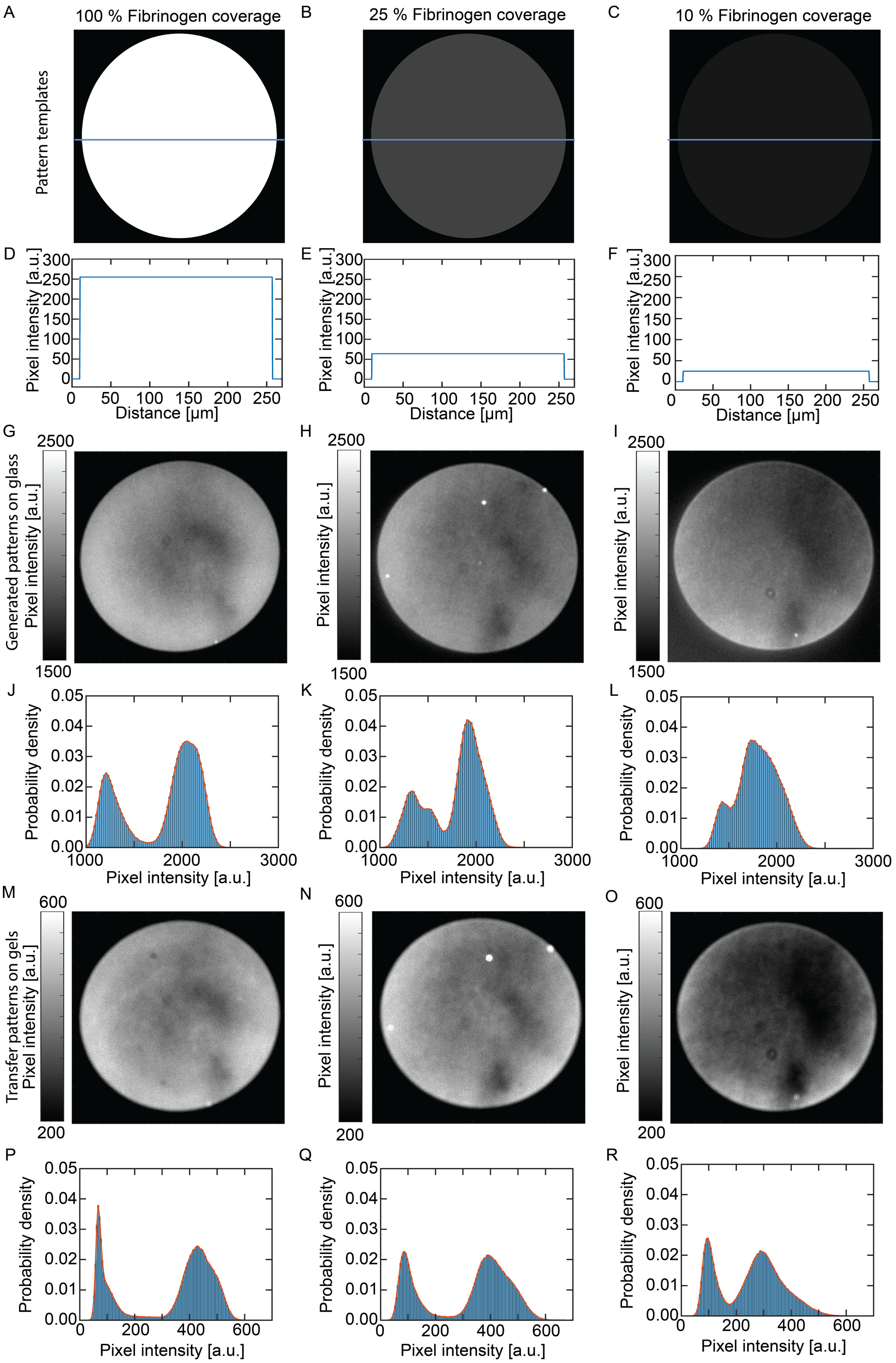

Fig. S2 (A, B, C) Template patterns in gray scale. The blue lines mark the line scans shown in (D, E, F). (G, H, I) Corresponding circular patterns with a diameter of 250 μm generated using LIMAP on glass after incubation with 100 μg/mL fluorescently labeled fibrinogen using a laser power of 2000 mJ/mm^2. (J, K, L) Corresponding histograms. (M, N, O) Circular patterns from (G, H, I) after transfer to the gels. (P, Q, R) Corresponding histograms.

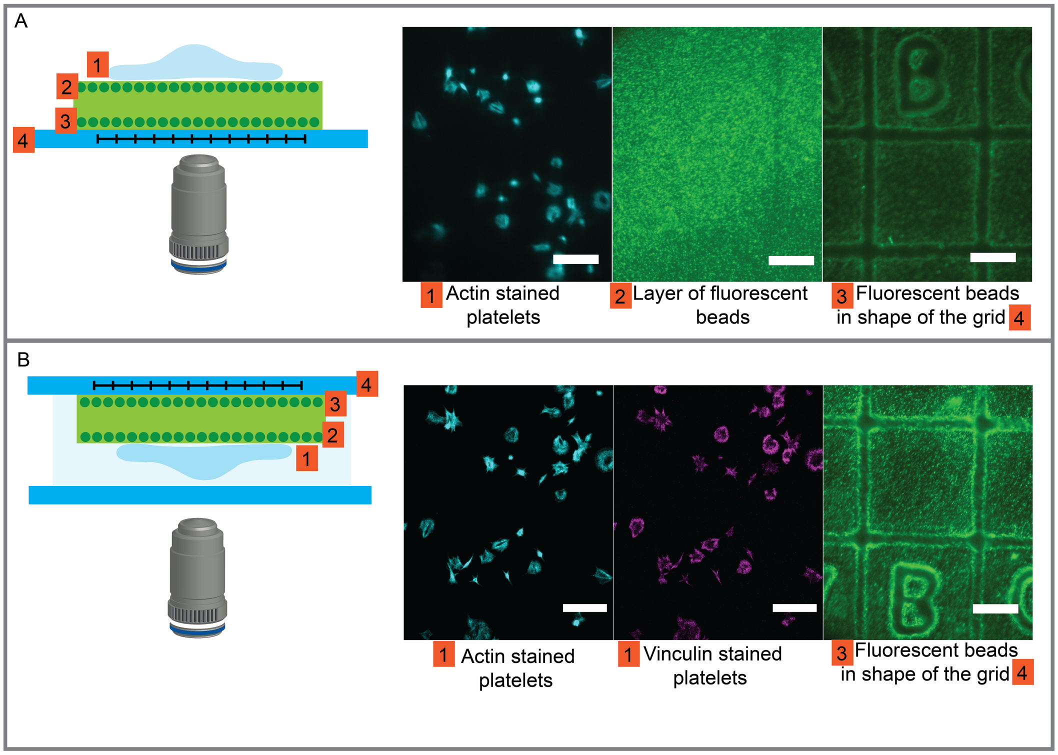

Fig. S3 Schematic of the procedure taken for superresolution imaging of platelets. A) Step one: life cell imaging and simultaneous TFM through the thick PAA gel with embedded fluorescent beads. (1) Shows an example image of actin structures (SiR-actin) in the platelets; (2) shows the bead layer just underneath the spreading platelet; (3) shows the grid pattern on the glass slide (4), which is used for relocalization of the same platelet. B) Step two: STED imaging of fixed platelets. (1) Shows the same actin structures as in (A), now at high resolution, and the corresponding vinculin structures (focal adhesions); (3) the beads take on the grid pattern and can be used for relocating the same cells. Scale bars correspond to 20 μm.

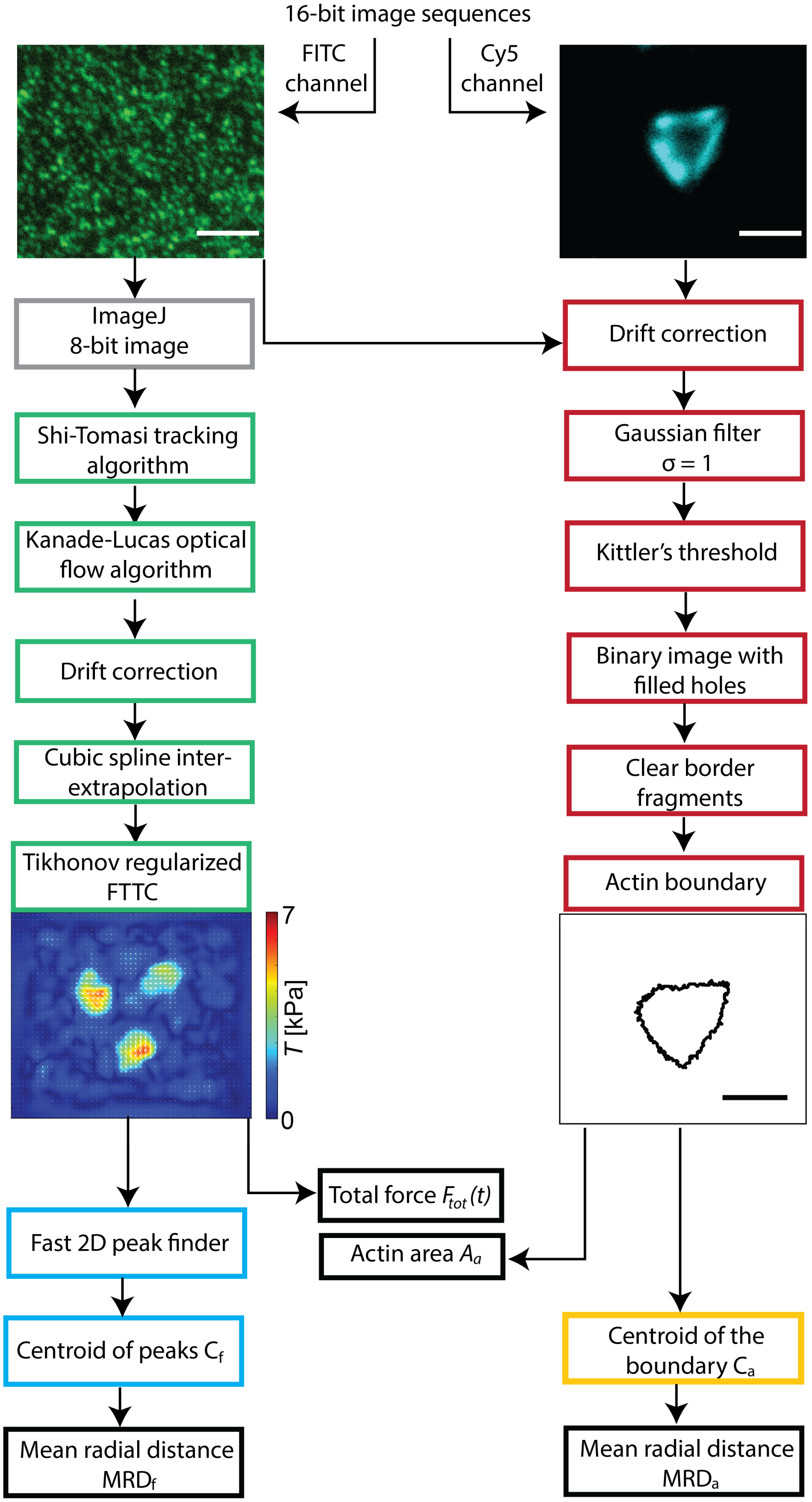

Fig. S4 Scheme of algorithms used for the analysis for traction force and actin area computation. The colors describe the classification of each step: green boxes show the individual steps in the algorithm for regularization-based approach of TFM, gray box shows input data for TFM, red boxes show the actin area determination, the yellow box shows the determination of the mean radial distance of the actin area MRD_a, blue boxes show the determination of the mean radial distance of the force MRD_f and black boxes show the data outputs. Scale bars correspond to 5 μm

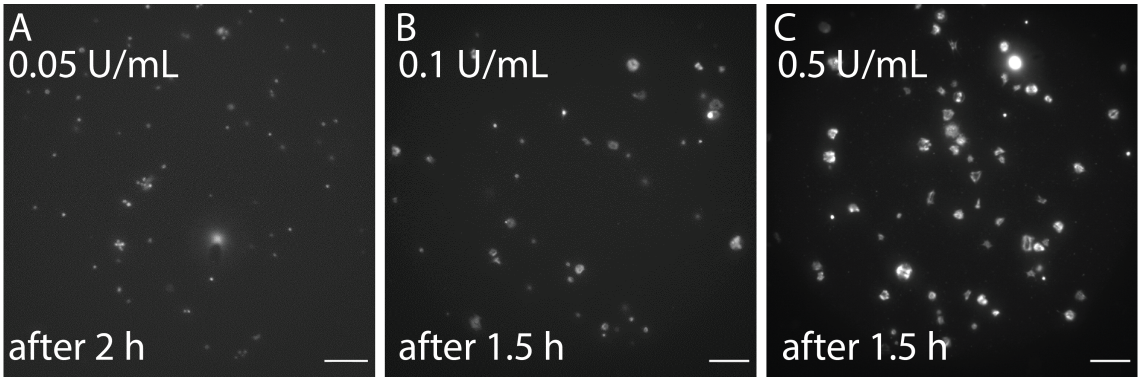

Fig. S5 Examples of activated platelets on gels labeled with SiR-actin after a minimum of 1.5 hours. A) Platelets activated by 0.05 U/mL thrombin. B) Platelets activated by 0.1 U/mL thrombin. C) Platelets activated by 0.5 U/mL thrombin. Scale bars correspond to 20 μm.

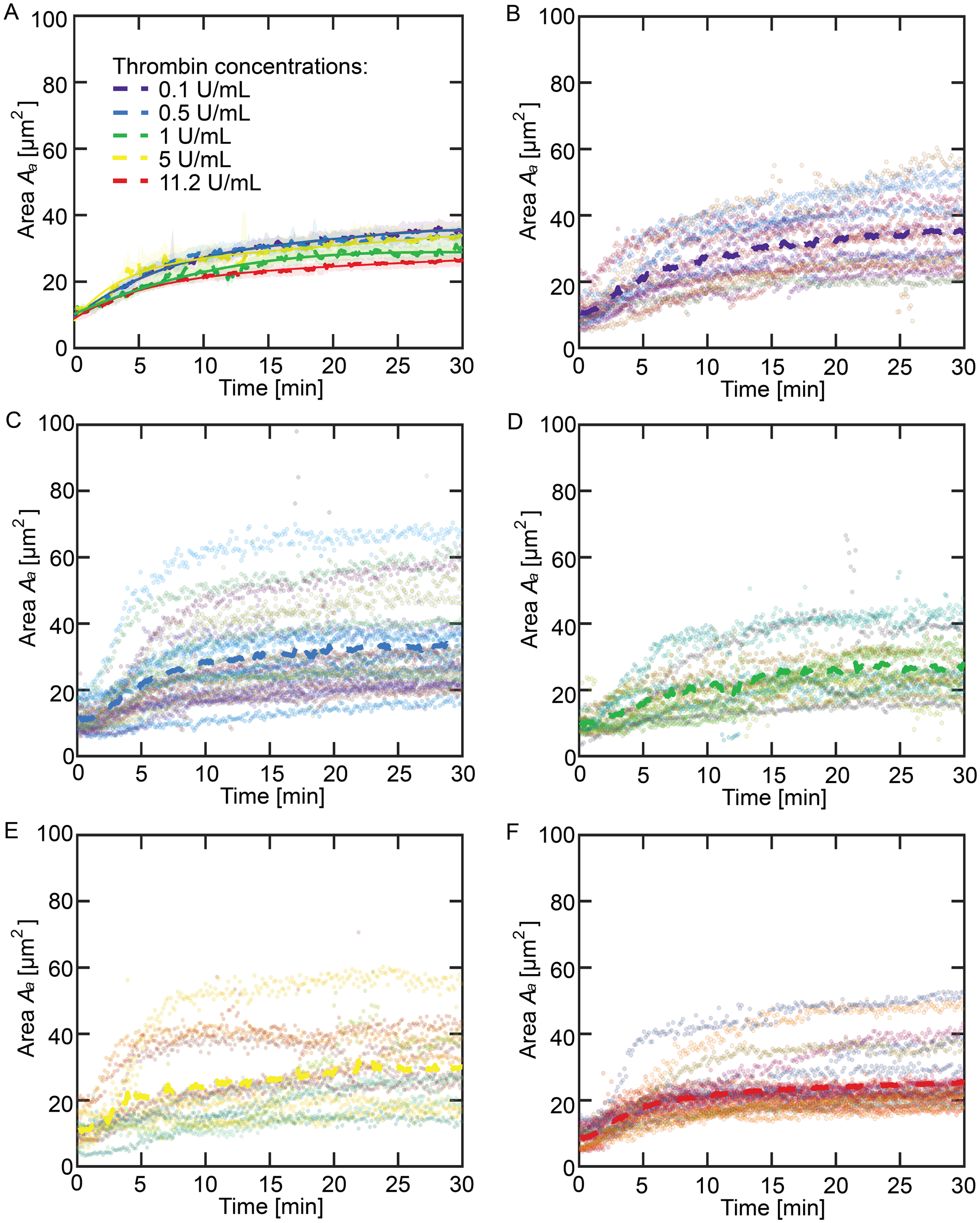

Fig. S6 The average time-dependent total area covered by actin structures $A_a(t)$ upon stimulation by different concentrations of thrombin. (A) Same plot as shown in Fig. 1C in the main text. Individual data curves for (B) 0.1 u/mL, (C) 0.5 U/mL, (D) 1 U/mL, (E) 5 U/mL, (F) 11.2 U/mL.

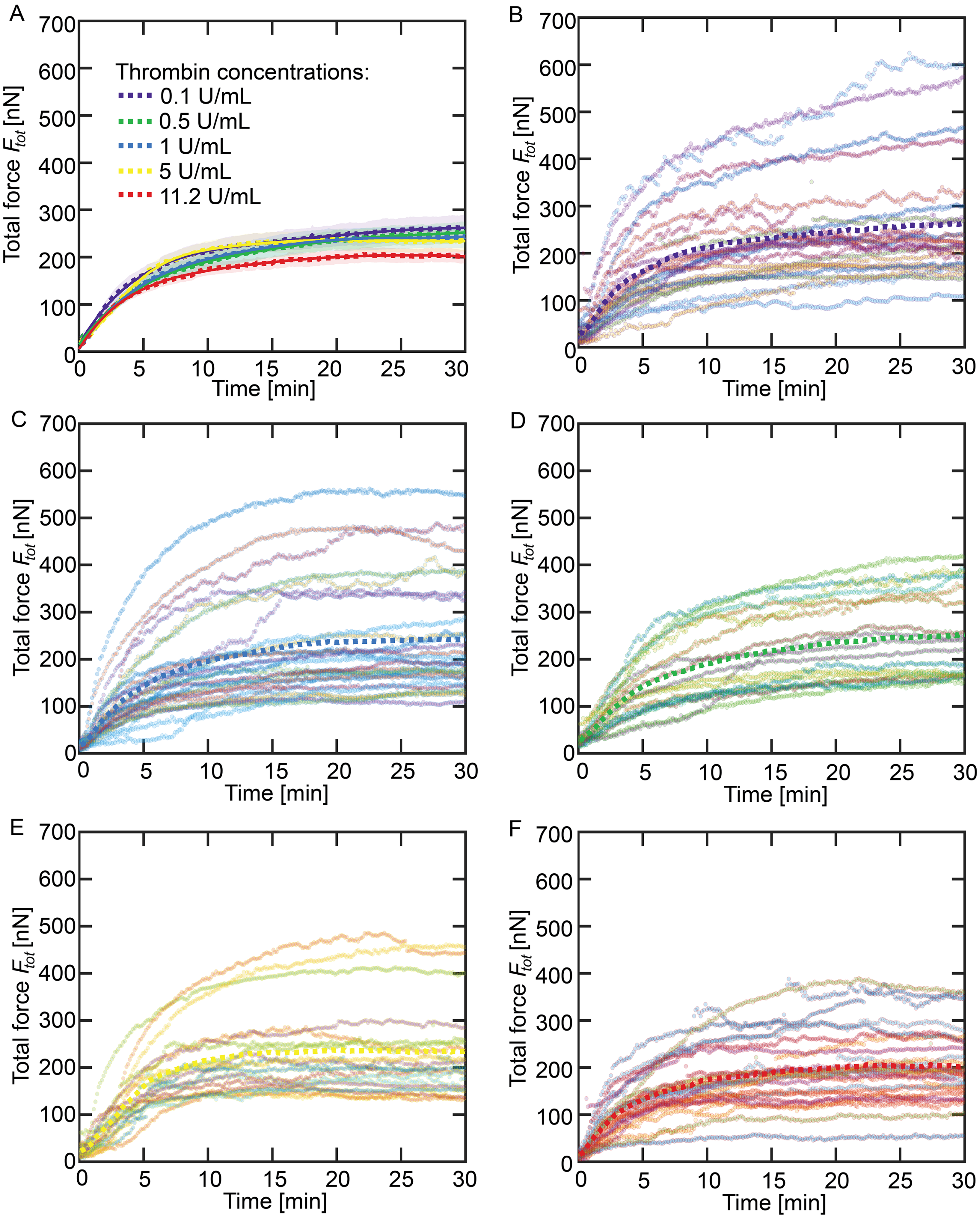

Fig. S7 Average total force curves for blood platelets upon stimulation by different concentrations of thrombin. (A) Same plot as shown in Fig. 1F in the main text. Individual data curves for (B) 0.1 u/mL, (C) 0.5 U/mL, (D) 1 U/mL, (E) 5 U/mL, (F) 11.2 U/mL.

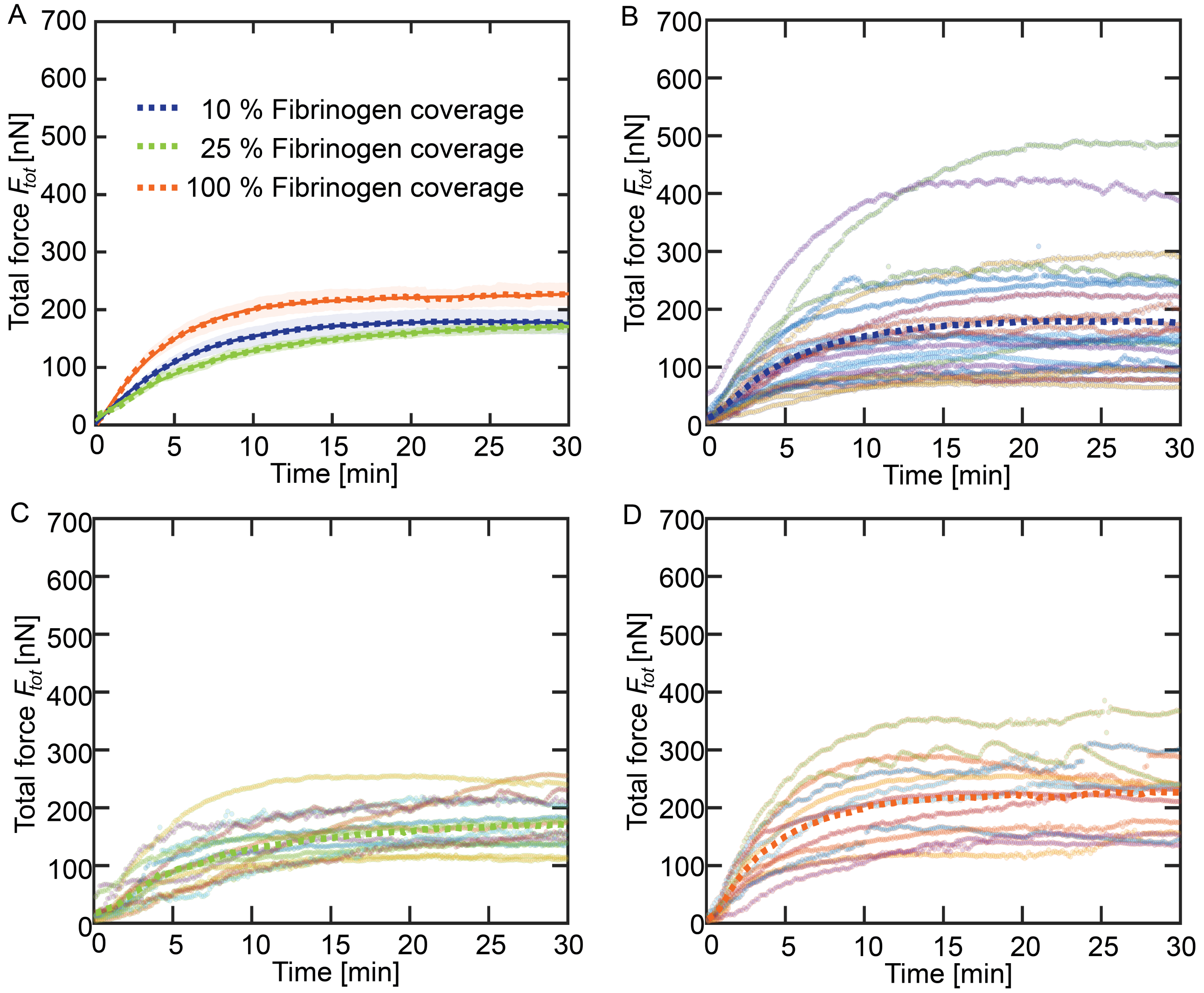

Fig. S8 Average total force curves for blood platelets upon stimulation by thrombin (0.1 U/ml) on gels covered by various concentrations of fibrinogen. (A) Same plot as shown in Fig. 2B in the main text. Individual data curves for (B) 10%, (C) 25%, (D) 100% fibrinogen coverage.

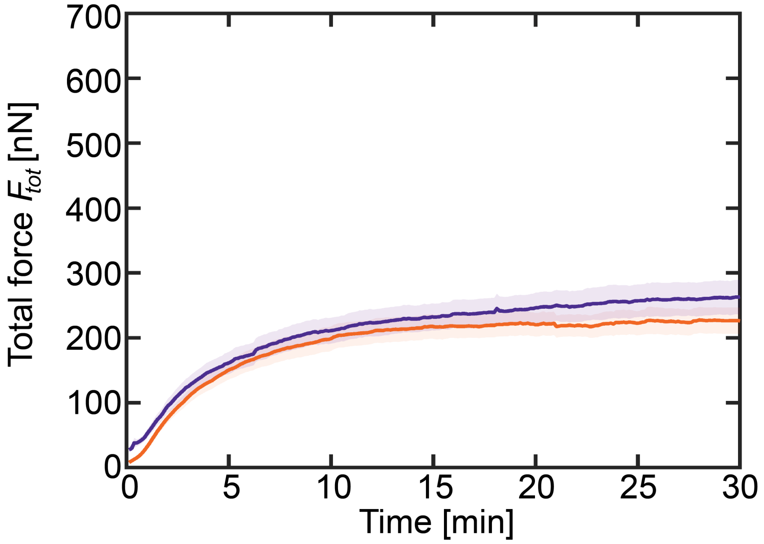

Fig. S9 Average total force curves for blood platelets upon stimulation by 0.1 U/mL thrombin on fibrinogen-coated gels obtained either by photo patterning (LIMAP) (orange line, the standard error is included as the orange transparent area) or by regular coating (blue line, the standard error is included as the blue transparent area).

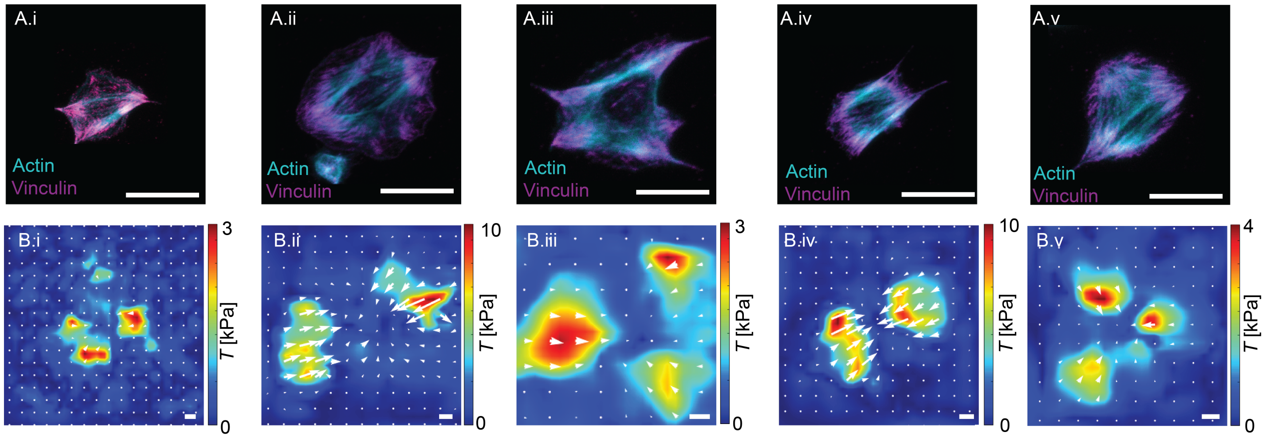

Fig. S10 STED-imaging of fixed and stained cells reveals details of cell organization during force generation. (A) Superresolution STED-imaging; actin in cyan and vinculin in magenta; the white scale bars correspond to 5 μm. (B) Traction forces before fixation with the white arrows indicating the direction of the force fields; the white scale bars correspond to 5 kPa.

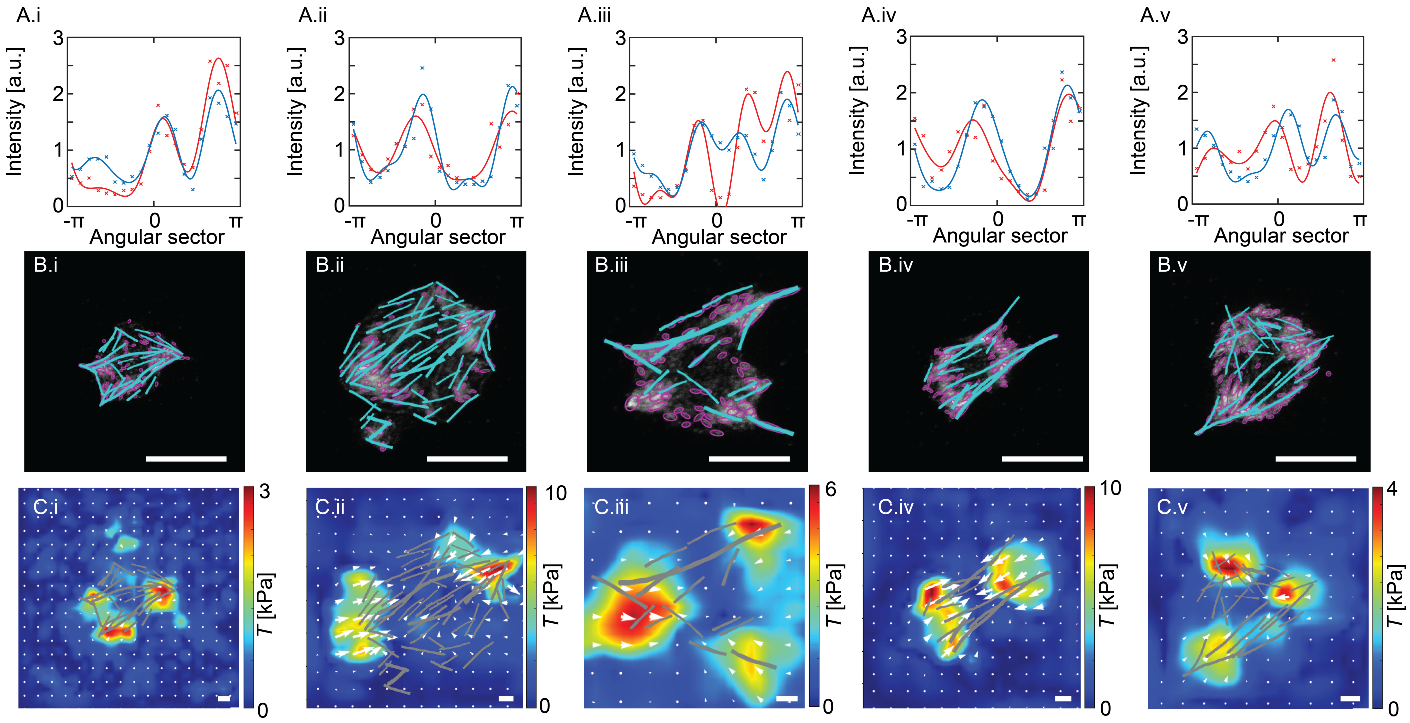

Fig. S11 Actin structures, vinculin patterns and force fields are correlated. The same cells (i) to (v) as in Fig. S10 are shown. (A) Circumferential profiles of the vinculin signal (red) and force magnitude (blue); the crosses show the data, the solid lines are fits. (B) Overlay of segmented vinculin spots (focal adhesions; magenta) and stress fibers (cyan); the white scale bars correspond to 5 μm. (C) Overlay of the traction forces field and the segmented stress fibers (gray); the white scale bars correspond to 5 kPa.

Captions of supplementary movies

Movie S1 Typical example for the development of the actin structure in a spreading platelet; 7.5 s per frame. Note that the contrast for each frame has been adjusted based on the intensity histogram for better optical visualization of early time points. Scale bar corresponds to 5 μm.

Movie S2 Temporal development of the traction force map, corresponding to Movie S1; with the detected boundary of the actin area indicated as black lines. The centroid is represented by a blue star. Magenta crosses represent the centers of the force hotspots and white arrows indicate the direction of the force field. For each image, the centroid of the hotspots is calculated and is represented as an orange cross. Scale bar corresponds to 5 μm.

Movie S3 Temporal development of the fluorescent bead positions used for calculation of traction force map. The data correspond to Movies S1 and S2. Scale bar corresponds to 5 μm.

Determination of adhesion sizes and orientations from the vinculin data

In order to extract the position of each adhesion, we first apply a 2nd order Sobel filter to compute the Laplacian. This identifies the points where the intensity is maximal. We apply a threshold to create a bitmap around these points. We then perform a connected component analysis and only retain sufficently large patches. For each component i, the orientation ϕ_i, the length a_i of its semi-mayor axis and its excentricity ε_i can be calculated using a moment analysis. We calculate the center of the adhesion $(x_0, y_0)^T$ as

$$\begin{pmatrix} x_0 \\ y_0 \end{pmatrix} = \int_{\Omega_i} \begin{pmatrix} x \\ y \end{pmatrix} \mathrm{d}x\,\mathrm{d}y,$$

where Ω_i is the area of the patch found in the component analysis. We then find the centered moment matrix M_i by

$$M_i = \int_{\Omega_i} \begin{pmatrix} (x-x_0)^2 & (x-x_0)(y-y_0) \\ (x-x_0)(y-y_0) & (y-y_0)^2 \end{pmatrix},$$

using summation to numerically solve the integral. If the adhesion was an ellipse, we would have for the moment matrix

$$M_{el} = \alpha a^4 \sqrt{1+\varepsilon^2} \begin{pmatrix} 1+\varepsilon^2 \sin^2(\phi) & \varepsilon^2 \cos(\phi)\sin(\phi) \\ \varepsilon^2 \cos(\phi)\sin(\phi) & 1+\varepsilon^2\cos^2(\phi) \end{pmatrix},$$

where the factor α takes into account that the threshold will only retain the innermost parts of the adhesion. By comparing both expressions for the moment matrix, we find that

$$\begin{aligned}
\phi &= \frac{1}{2}\arctan 2(2M_{i,xy}, M_{i,xx} - M_{i,yy}) \\
S &= \alpha a^4 \sqrt{1+\varepsilon^2}(2+\varepsilon^2) = M_{i,xx} + M_{i,yy} \\
T &= \alpha a^4 \sqrt{1+\varepsilon^2}\varepsilon^2 = \sqrt{(M_{i,xx} - M_{i,yy})^2 + 4 * M_{i,xy}} \\
\varepsilon &= \sqrt{\frac{2T}{S-T}} \\
a &= \left(\frac{2T}{\alpha\sqrt{1+\varepsilon^2}\varepsilon^2}\right)^{1/4}.
\end{aligned}$$

The $\arctan 2$ function follows the usual convention, such that $\arctan 2(y,x) = \arctan(y/x)$ for positiv y and x values. In our analysis, we empirically selected a threshold value of -2.5, retained components comprimising more them 25 pixels and used a scaling factor $\alpha = 3^{-4}$.
