# Supplementary material for: Force generation in human blood platelets by filamentous actomyosin structures
Source: Biophys J. 2023 Jul 20;122(16):3340–53. doi: 10.1016/j.bpj.2023.07.010 (PMC10465724; doi:10.1016/j.bpj.2023.07.010)
Supplement: Document S2. Article plus supporting material [file mmc5.pdf]

Biophysical Journal
Article

Biophysical Society

Force generation in human blood platelets by filamentous actomyosin structures

Anna Zelená,[1] Johannes Blumberg,[2,3] Dimitri Probst,[2,3] Rūta Gerasimaitė,[4] Gražvydas Lukinavičius,[4] Ulrich S. Schwarz,[2,3,*] and Sarah Köster[1,5,6,*]
[1]Institute for X-Ray Physics, University of Göttingen, Göttingen, Germany; [2]Institute for Theoretical Physics, University of Heidelberg, Heidelberg, Germany; [3]BioQuant-Center for Quantitative Biology, University of Heidelberg, Heidelberg, Germany; [4]Max Planck Institute for Multidisciplinary Sciences, Göttingen, Germany; [5]German Center for Cardiovascular Research (DZHK), Partner Site Göttingen, Göttingen, Germany; and [6]Cluster of Excellence "Multiscale Bioimaging: from Molecular Machines to Networks of Excitable Cells" (MBExC), University of Göttingen, Göttingen, Germany

ABSTRACT Blood platelets are central elements of the blood clotting response after wounding. Upon vessel damage, they bind to the surrounding matrix and contract the forming thrombus, thus helping to restore normal blood circulation. The hemostatic function of platelets is directly connected to their mechanics and cytoskeletal organization. The reorganization of the platelet cytoskeleton during spreading occurs within minutes and leads to the formation of contractile actomyosin bundles, but it is not known if there is a direct correlation between the emerging actin structures and the force field that is exerted to the environment. In this study, we combine fluorescence imaging of the actin structures with simultaneous traction force measurements in a time-resolved manner. In addition, we image the final states with superresolution microscopy. We find that both the force fields and the cell shapes have clear geometrical patterns defined by stress fibers. Force generation is localized in a few hotspots, which appear early during spreading, and, in the mature state, anchor stress fibers in focal adhesions. Moreover, we show that, for a gel stiffness in the physiological range, force generation is a very robust mechanism and we observe no systematic dependence on the amount of added thrombin in solution or fibrinogen coverage on the substrate, suggesting that force generation after platelet activation is a threshold phenomenon that ensures reliable thrombus contraction in diverse environments.

SIGNIFICANCE Blood clotting is an essential physiological process and requires precise control: decreased and increased clotting both can have fatal consequences. The major cellular players in blood clotting are platelets. Upon activation these tiny cell fragments spread out at the site of injury, contract, and eventually form the blood clot, a cellular aggregate held together by a fibrinogen matrix. Combining live-cell imaging, traction force microscopy, and superresolution imaging, we demonstrate that cytoskeletal structure formation and force generation are strongly correlated. Force is localized in focal adhesions at the endpoints of a few actomyosin bundles arranged in simple geometrical patterns and the resulting force is not systematically dependent on thrombin concentration and fibrinogen coverage, suggesting a very robust mechanism of force generation.

INTRODUCTION

Blood platelets are essential for blood clotting and primary hemostasis, and thus play an important role in wound healing. Platelets are non-nucleated fragments of megakaryocytes (1) with a large variability between the individual cells. They do not contain any microtubule organizing center (2), but the cytoskeletal filaments F-actin, microtubules (1), desmin, and vimentin intermediate filaments (3), as well as different types of molecular motors, including dynein, which slides microtubules (4), and myosin, which contracts actin filaments (5). In the resting state, the platelets circulate in the blood stream as small discoids, shaped by the so-called marginal band consisting of a microtubule coil (1). The activation of platelets can be triggered by various reagents such as adenosine diphosphate (ADP), thrombin, and serotonin (6) but also by mechanical shear (7). The most common activator of platelets is thrombin, which is also responsible for stabilization of the blood clot

Submitted September 21, 2022, and accepted for publication July 17, 2023.
*Correspondence: schwarz@thphys.uni-heidelberg.de or sarah.koester@uni-goettingen.de
Anna Zelená and Johannes Blumberg contributed equally to this work.
Editor: Kinneret Keren.
https://doi.org/10.1016/j.bpj.2023.07.010
© 2023 Biophysical Society.
This is an open access article under the CC BY license (http://creativecommons.org/licenses/by/4.0/).

by enzymatic conversion of fibrinogen into fibrous fibrin (1,8–10). Once the platelets are activated, the marginal band first expands due to motor activity and then coils under the opposing force of the actomyosin cortex (4,11–13). Therefore, platelets show a spherical shape after activation. At the same time, they activate integrin-based adhesion to matrix proteins. Therefore, on a flat substrate, they strongly adhere and show a very flat and thin organization (14). Platelets first form filopodia, then thin lamelopodia (15–20), and finally contractile actomyosin bundles, which, in contrast to other adherent cell types, tend to be excluded from the cell center that typically is filled with granules and the coiled microtubules resulting from the marginal band (21,22).

In summary, actomyosin is essential for force generation and platelet contraction. To gain a better understanding of the underlying mechanisms, direct—ideally dynamic—imaging of actin structures is of great importance. However, due to the lack of a nucleus, transfections are not possible and most experiments rely on staining of actin structures in fixed platelets by phalloidin. Using superresolution fluorescence microscopy in combination with electron microscopy, actin structures have been imaged at high resolution (23). The overall shapes of actin structures in platelets have been categorized and characterized by morphometric analysis, depending on different protein coatings and different substrate stiffnesses (23). Notably, platelets from mice expressing LifeAct-GFP have been employed as well (22,24). These experiments revealed that platelets spread via actin-dependent structures, i.e., filopodia, which are then filled by lamellipodia. The recently introduced silicon-rhodamine (SiR) actin probe (25) allows for life staining of actin in human platelets as well, and first experiments revealed a time constant of 245 s $\pm$ 144 s for the formation of the complete actin cytoskeleton, compared to a faster spreading of the platelet membrane with a time constant of 68 s $\pm$ 34 s (26).

Complementing imaging studies and motivated by the central role of force generation by platelets, force measurements have been performed by, e.g., bulk rheology measurements (27) and on so-called microthrombi on elastic micro-pillars (28). To distinguish effects by the platelets from influences of the matrix that connects them, single-cell measurements have been performed. Atomic force microscopy (AFM) reveals an average contractile force of 19 $\pm$ 3.1 nN (29). A similar force range, albeit slightly higher, was obtained from platelet contraction cytometry, based on the displacement of fibrinogen-coated microdot pairs on hydrogels (30). The forces are in the range of 20–50 nN. By staining fixed platelets, the authors conclude that actin is involved in platelet force generation.

Traction force microscopy (TFM), rather than measuring total forces only, allows for computing spatio-temporally resolved force fields. Experiments with soft gels (4 kPa) have shown contractile forces in the range of 34 nN 25 min after platelet attachment to the gels (31). Further development of the experimental setup and data analysis procedures has shown that forces produced by platelets can reach an averaged value of 200 nN in the range of 30 min. Moreover, the increased spatial resolution has allowed us to detect so-called force hotspots of the traction forces at the periphery of the platelets (32). Thus, the force levels obtained by TFM are about an order of magnitude higher than values from experiments with other methods, possibly because the platelets are very flat and exert their force in a more distributed manner on flat substrates.

The imaging experiments of actin structures in platelets along with force measurements suggest that the actomyosin system is responsible for platelet spreading, shape dynamics, and contraction. However, a direct combination of imaging and force measurements has not been achieved before. Therefore, here, we combine time-resolved TFM with simultaneous live imaging of actin to quantify and correlate actin structures and force fields in a time-resolved manner. Interestingly, the force hotspots that have been observed before are spatially and temporally correlated with the endpoints of the actomyosin fiber structures. We confirm these findings by superresolution STED microscopy of actin and vinculin at the endpoint of the spreading process. We thus provide the first direct proof of force generation by fibrous actomyosin structures in platelets. For a gel stiffness in the physiological range, we do not observe a systematic dependence of force generation on the thrombin concentration in solution or the fibrinogen coverage of the substrate.

MATERIALS AND METHODS

Platelet purification and live-cell actin staining

The experiments are performed in accordance with the Ethics Committee of the University Medical Center Göttingen, votum 11/11/09. Platelet concentrates are obtained from healthy donors. Platelets are purified as previously described (31–33). In brief, 4 mL of the platelet concentrate is transferred into a tube together with prostaglandin E1 (PGE1 2.6 μg/mL, Cayman Chemical Company, Ann Harbor, MI, USA). The content is mixed and centrifuged for 20 min at 480 $\times$ g and 21°C. The supernatant is replaced by PIPES saline glucose buffer (PSG; i.e., 5 mM PIPES, 145 mM NaCl, 5 mM glucose, 4 mM KCl, 1 mM $MgCl_2$, 0.05 mM Na_2HPO_4, pH 6.8) with PGE1. The centrifugation step is repeated twice. After the last removal of the supernatant, the pellet is resuspended in HEPES-Tyrode buffer (HT; i.e., 134 mM NaCl, 12 mM $NaHCO_3$, 2.9 mM KCl, 1 mM $MgCl_2$, 5 mM HEPES, 5 mM glucose, 0.34 mM NaH_2PO_4, pH 7.4) with bovine serum albumin (BSA; 5 mg/mL, Macs BSA stock solution, Milteny Biotech, Bergisch Gladbach, Germany). The platelets are counted using a hematocrit capillary (sodium heparinized 75 mm/75 μL, Hirschmann Laborgeräte, Eberstadt, Germany) and diluted to a final concentration of 2×10^7 cells/mL. After purification and until the start of the experiments, the platelet suspension is kept on a cell rotor (MACSmix Tube Rotator, Miltenyi Biotech, Bergisch, Gladbach, Germany). The cell suspension is then used within 4 h. SiR-actin (final concentration 0.5 μM, exitation/emission: 652/674 nm, Spirochrome, Stein am Rhein, Switzerland) is added to the diluted platelet suspension and incubated for 15 min before the samples are used in the experiments.

Fabrication of polyacrylamide substrates

For the live-cell experiments, the protocol for gel preparation is adapted from (34). Square glass coverslips (24 mm × 24 mm no. 1, VWR, Radnor, PA, USA) are cleaned with isopropanol and dried under a nitrogen stream. Subsequently, both sides of the glass coverslips are exposed to an air plasma (0.5 mbar, ZEPTO, Diener Electronics, Ebhausen, Germany) for 1.5 min at 50 W. Next, the coverslips are soaked in a silanization solution consisting of 2% (v/v) 3-(trimethoxysilyl)propyl methacrylate (Sigma-Aldrich, St. Louis, MO, USA) and 1% (v/v) acetic acid (100%, Carl-Roth, Karlsruhe, Germany) in absolute ethanol (≥99.8%, Carl-Roth) for 10 min. The excess of the solution is aspirated and the coverslips are rinsed with ethanol. Air-dried silanized coverslips are baked at 120°C for 1 h and stored at room temperature (RT).

For the experiments employing different thrombin concentrations, circular glass coverslips (Ø 18 mm no. 1, VWR) are cleaned with isopropanol, dried under a nitrogen stream, coated with PlusOne Repel-Silane (GE Healthcare, Little Chalfont, UK) from both sides for 5 min, and washed with ethanol. A polyacrylamide (PAA) mixture for a final gel stiffness of 34 kPa is prepared by mixing 2.5 mL of acrylamide (40%, Bio-Rad Laboratories, Hercules, CA, USA), 0.75 mL of bis-acrylamide (2%, Bio-Rad Laboratories), and 6.75 mL of phosphate-buffered saline (1× PBS; i.e., 137 mM NaCl, 2.7 mM KCl, 10 mM Na_2 HPO_4, 1.8 mM kH_2 PO_4, pH 7.2). This stiffness corresponds to healthy femoral artery tissue (35). Then 10 μL of carboxylated beads (0.2% solids, FluoSpheres, carboxylate-modified microspheres, 40 nm in diameter, yellow-green, 505/515 nm, Thermo Fisher Scientific, Waltham, MA, USA) are added to 490 μL of the PAA solution. The addition of 10 μL ammonium persulfate (10% APS, Bio-Rad Laboratories) and 0.5 μL of TEMED (N,N,N′,N′-tetramethylethane-1,2-diamine, Bio-Rad Laboratories) initiates the polymerization. The PAA solution is mixed and 10 μL are pipetted on a circular, PlusOne Repel-Silane-treated coverslip and covered by a squared silanized coverslip. The gel is polymerized upside down for 1 h. After the polymerization, the gel is soaked in PBS and the circular glass coverslip is removed. The top layer of the gel is coated twice with Sulfo-SANPAH (0.4 mM in 50 mM HEPES buffer (pH 8), Thermo Fisher Scientific) by applying UV light for 8 min (365 nm 2 tube 8 W, HerolabLaborgeräte, Wiesloch, Germany). Subsequently, 150 μL of fibrinogen solution (100 μg/mL, CalBiochem-Merck, Darmstadt, Germany) is added on top of the gel and incubated overnight at 5°C to achieve uniform coverage. Coated gels are washed with PBS and kept submerged in PBS until used in the experiments.

For testing different degrees of fibrinogen coating, we use micropatterned substrates. To do so, we employ light-induced molecular adsorption of proteins (LIMAP) using a PRIMO setup (Alveóle, Paris, France) installed on an inverted fluorescence microscope (IX83, Olympus, Hamburg, Germany) equipped with a 20× objective (LUCPLFLN,/0.45 N.A. air, Olympus) and controlled via the software Leonardo (Alveóle). Circular glass coverslips (Ø 20 mm no. 1, VWR) are washed with isopropanol, dried under a nitrogen stream, and plasma treated for 3 min at 50 W. A polydimethylsiloxane (PDMS) stencil with a well of Ø 3.5 mm is placed on top of the plasma-treated coverslip. The glass coverslip area inside the well is passivated using 10 μL of PLL-g-PEG (0.1 mg/mL in PBS (PLL(20)-g[3.5]-PEG (2 kDa)), SuSoS, Dübendorf, Switzerland) and incubated for 1 h, followed by washing three times with PBS. After removing the PBS, the empty well is filled with 5 μL of photo-initiator (Product of Liaison for Protein Patterning (PLPP), Alveóle). The PLPP degrades the passivated layer of PLL-g-PEG after exposure to UV light to a degree that is linearly dependent on the dose (36). The maximum dose we use for printing a pattern is 2000 mJ/mm^2 corresponding to 100% fibrinogen coverage. According the linear dependence between dose and coverage, we also prepare samples with 25% and 10% of the dose. After exposure to the UV light, the glass area inside the well is washed three times with PBS and subsequently filled by 10 μL of fibrinogen solution (100 μg/mL, CalBiochem-Merck). The protein is incubated inside of the well for 2 h at 5°C and washed three times with PBS. Successful patterning and transfer to the gel is proved by using fluorescently labeled fibrinogen (100 μg/mL, Alexa Fluor 488 conjugate, excitation/emission: 495/519 nm, Invitrogen, Darmstadt, Germany; see supporting material, Figs. S1 and S2). To transfer the fibrinogen patterns from the glass coverslips to PAA gels, the PDMS stencil is removed and the glass coverslip is dried by blotting with tissues. A PAA mixture is prepared as described above (note that in this case the fluorescent beads are passivated with PLL-g-PEG (0.1 mg/mL in PBS)), pipetted to the top of the circular, patterned coverslip, and covered by a squared, silanized coverslip. After 1 h, the gels are soaked in PBS and the top glasses with the pattern are gently removed. The surface of the gel now presents the previously produced fibrinogen pattern.

For experiments with confocal and stimulated emission depletion (STED) microscopy, the PAA gels are produced similarly to the protocol described above. We use round glass coverslips with a 50 μm grid (Ø 28 mm no. 1.5 Schott glass, Ibidi, Martinsried, Germany) to locate of cells after fixation. A clean coverslip is exposed to air plasma (0.5 mbar) for 30 s at 40 W, followed by a 5-min incubation with 3-aminopropyltrimethoxysilane (APTMS; Sigma-Aldrich). Subsequently, the coverslip is rinsed with ultrapure water, dried, and treated with 0.5% glutaraldehyde (70% EM grade, Polysciences, Warrington, PA, USA) in PBS, and incubated for 30 min at RT. After incubation, the coverslip is washed with ultrapure water and dried. The dried coverslips are used for the fabrication of PAA gels as described above. The polymerized gels are coated with Sulfo-SANPAH and incubated with fibrinogen (100 μg/mL).

Live-cell TFM and epi-fluorescence imaging

All TFM experiments, independent of the gel preparation procedure, are conducted on an inverted microscope (IX81, Olympus), equipped with a 60× water immersion objective (UPlanSApo, numerical aperture (NA) = 1.2, working distance (WD) = 0.28 mm, Olympus) an MT20 Xenon arc lamp (150 W, Olympus) lamp operated at 23% of the maximum intensity and a CMOS (complementary metal-oxide-semiconductor) camera (Orca Flash 4.0, Hamamatsu Photonics Deutschland, Herrsching am Ammersee, Germany). Prepared PAA gels are washed with HT buffer containing BSA and placed inside an incubation chamber (Stage Top Incubator STX, Tokai Hit, Fujinomiya, Shizuoka-ken, Japan), including a home-built sample holder optimized for 24 × 24-mm coverslips. The incubation chamber guarantees stable physiological conditions of 5% CO_2 and 37°C throughout the experiment. All gels have a thickness of at least 50 μm and we select areas with homogeneous bead coverage for the experiments. Fig. 1 *A* shows a schematic representation of the experimental setup.

Ninety microliters of SiR-actin-stained platelet suspension are pipetted on the prewarmed PAA gel. The average number of platelets on the recorded area after 2 h is 1.5 × 10^5 cm^{-2}. We analyze only platelets that do not interact with other platelets. Ten microliters of thrombin solution (thrombin from human plasma, activity 2800 U/mg (U denotes NIH-units here), Sigma-Aldrich; diluted in HT with BSA) is added directly before the recording starts to activate the platelets. The final thrombin concentration varies from 0.05 to 11.2 U/mL. The different fibrinogen coatings are investigated using a thrombin concentration of 0.1 U/mL. For the fluorescence imaging, we employ a dual-band filter (FITC/Cy5; excitation at 470 and 628 nm and emission at 537 and 694 nm (AHF Analysentechnik, Tübingen, Germany)). We record the platelet spreading for 2 h at one frame every 7.5 s with an exposure time of 50 ms each for the actin structures and the beads. We chose a total recording time of 2 h to allow different platelets to adhere and start spreading at different time points and so as to capture 30 min of spreading and contraction time for each platelet. After 30 min, the platelets are fully contracted. Some remain in this contracted state, whereas, for some, the underlying gel relaxes, possibly due to detachment of the platelet.

Superresolution imaging

For experiments that combine live-cell TFM with confocal and STED microscopy on fixed platelets, we proceed in two steps. In a first step (see supporting material, Fig. S3 *A*) platelets are stained by 0.5 μM SiR-actin and activated by adding thrombin (0.1 U/mL). We record adhering, spreading, and contracting platelets for 2 h through the gel to obtain TFM data.

In a second step, platelets are fixed and permeabilized by 0.1% (v/v) Triton-X-100 (Carl-Roth) and 4% (w/v) formaldehyde (16%, methanol free, Thermo Fisher Scientific) in cytoskeleton buffer (9.3 mM MES, 128.34 mM KCl, 2.79 mM MgCl, 1.86 mM EGTA, pH 6.1 adjusted with NaOH) with 10% sucrose (Sigma-Aldrich) for 15–20 min and rinsed three times with PBS at RT. After the fixation, the samples are incubated with $NaBH_4$ (30 mM sodium borohydride, Fluka/Honeywell, Charlotte, NC, USA) in PBS for 10 min to quench autofluorescence. After washing with PBS, the samples are incubated with a blocking solution containing 0.1% (v/v) Tween20 (Carl-Roth) and 3% (w/v) BSA (Capricorn Scientific, Ebsdorfergrund, Germany) in PBS for 30 min at RT. Subsequently, the gels with the fixed platelets are incubated with a primary antibody against vinculin (dilution 1:80, monoclonal mouse anti-vinculin V9131, Sigma-Aldrich) in blocking solution for 40 to 50 min at RT. After washing with PBS containing 0.1% (v/v) Tween 20, the samples are incubated with a secondary antibody (anti-mouse) labeled with Abberior STAR 580 (1:400 2 mg/mL, Abberior, Göttingen, Germany) and additionally 0.5 μM SiR-actin in blocking solution for 1 h at RT. After washing with PBS containing 0.1% (v/v) Tween 20, samples are rinsed with ultrapure water and mounted on glass coverslips (Ø 25 mm no. 1.5, VWR) with ProLong Diamond antifade Mountant (Invitrogen) and left to polymerize for 1 day at RT. These samples are imaged through the glass slide using superresolution STED microscopy (see supporting material, Fig. S3 *B*), using an Abberior STED Facility Line microscope (Abberior Instruments, Göttingen, Germany) built on a motorized inverted microscope IX83 (Olympus) equipped with an 60× silicone oil immersion objective (UPlanSApo, NA = 1.3, WD = 0.3 mm, Olympus). For this setup, the pixel size is 30 nm and the pinhole is set to 0.92 AU (Airy units with 1 AU = 1.22 λ/NA) for STED imaging. The dwell time is set to 20 μs. The fluorescent probe SiR-actin is excited by 2% power of a 640-nm laser and detected in the emission range 650–755 nm; for STED, we use a 775-nm laser with 10% power; we scan each line 10 times. The fluorescent probe STAR 580 is excited by 50% power of a 561-nm laser and detected in the emission range 571–630 nm; for STED, we use a 775-nm laser with 30% power; we scan each line 10 times. The spherical correction is set to 1 to improve the image quality.

Data analysis

For analysis of the TFM data, we employ optical flow using the Kanade-Lucas-Tomasi (KLT) algorithm with a regularization-based approach (32). Briefly, the image sequences with the recorded beads are downsampled from 16 to 8 bit in ImageJ (37). The Shi-Tomasi corner detector tracks to find position of maximum 1000 beads. To avoid double counting of clusters, a minimum distance between beads is set to 3 pixels. The pyramidal KLT algorithm is used to track the intensity gradients around the detected beads within search windows of size 48 × 48 pixels and in the second round of size 24 × 24 pixels. The displacements are tracked between successive images and the complete trace is reconstructed with reference to the undeformed image before adhesion of the platelet. We assume a linear movement inside each of the search windows using a forward Euler approach. The drift correction is applied on a small area of the image sequence without deformation and the detected drift is subtracted from all calculated displacements. The drift-corrected displacements are interpolated onto a regular 4 × 4 pixels grid. Application of a Tukey filter (α = 0.2 for the cells shown in Figs. 3 *A* and 5 *A*, α = 0.4 for Fig. 6 *B*.i to v; Fig. 7 *C*.i to v and in the supporting material) ensures a zero velocity field at the border of the grid with drift-corrected displacements. The traction forces are calculated by Fourier transform traction cytometry (FTTC) with Tikhonov regularization using regularization parameters defined by a generalized cross-validation (GCV) function. The total force F_{tot} is given by

$$F_{tot} = \int_{A_{roi}} \left| \vec{T}(\vec{x}) \right| \mathrm{dA}_{\mathrm{roi},} \tag{1}$$

where $\vec{T}(\vec{x})$ is the traction vector at a given position $\vec{x}$ and A_{roi} defines the selected area containing a single contracting platelet.

The time point t_0 for each video is defined such that it corresponds to the starting point of contraction determined from the total force $F_{tot}(t)$: the curve is smoothed using the "rloess" function in MATLAB (38), which reduces the effect of outliers and t_0 is defined as the time point of the first maximum of the second derivative of the smoothed $F_{tot}(t)$. The area $A_a(t)$ covered by actin at any given time point in the image sequence corresponds to the platelet spread area. It is determined by first applying a Gaussian filter with standard deviation 1 on the image sequence to reduce the noise. The smoothing is essential in particular for the analysis of the early time points. Next, we threshold the image following Kittler's method of minimum error thresholding (39). The method performs well in cases where the distributions of the object and background have unequal variances. The finally obtained area A_a is cleaned of unconnected segments and plotted against time. For large peaks in the signal due to detection of inseparable clusters of floating platelets that do, however, not attach to the platelet of interest, the data are interpolated using the nearest neighboring non-outlier values. Outliers are defined as area values more than three standard deviations from the mean. A detailed description of the algorithm used here can be found in the supporting material in Fig. S4.

The resolution of the traction reconstruction is predominantly determined by the spacing between neighboring beads and the interpolation grid spacing. Assuming that the bead positions are uncorrelated, we can use a Poisson distribution to describe the local bead density. From this distribution, the average distance between neighboring beads can be estimated to be

$$\langle r \rangle = \frac{1}{2}\sqrt{\frac{hw}{N_B}} \tag{2}$$

where h and w are the height and width, respectively, of the area recorded in the image, and N_B is the number of the detected beads. For a typical image we found $h = w = 25$ μm and $N_B = 1000$. This results in an average bead spacing $\langle r \rangle = 395$ nm, that can be resolved with the optical microscope and justifies the average grid spacing of 400 nm used in the regular grid interpolation.

We quantify the correlation between the intensity of the vinculin immunostaining and the magnitude of the traction forces using previously published MATLAB scripts by Lickert et al. (23). First, the script "Define Cells" is used to determine the centroid of the actin boundary. Second, the script "Process Cells" is applied to divide the vinculin images and the traction force maps into 20 equal angular sectors that originate from the centroid determined before. The intensity values in each sector are averaged and plotted against the azimuthal angle (red crosses for vinculin, blue crosses for force magnitude). These circumferential profiles are fitted to the function $Y(\alpha) = 1 + a \cdot \cos(\alpha) + b \cdot \sin(\alpha)$, where α is the azimuthal angle.

To extract the stress fibers, we use a combination of the MATLAB scripts by Lickert et al. (23) and the tool FilamentSensor (40). We again use the scripts Define Cells and Process Cells, but this time to obtain a binarized bitmap of the actin staining as well as a prediction of the local stress fiber orientation angle. We then determine the dominant orientation angle and produce two bitmap images: one in which only those bits of the actin image are retained, in which the corresponding

orientation angle is roughly parallel to the dominant orientation angle (angles differ by less than 45°), and one in which only those bits of the actin image are retained in which this is not the case. We found that this procedure of processing stress fibers in these two groups improves their reconstruction. Both bitmap images are then processed independently using the FilamentSensor. We load the images using the "Stack View" option and select "As straight pieces" in the "Line Sensor" menu to obtain the filaments in a segment-wise manner. We then use the "Export filaments as .cs" option to extract the segments. In a post-processing step, we first assemble the segments into piecewise straight lines and then merge the filament datasets obtained from the parallel and non-parallel bitmap images.

To compare the localization of vinculin to actin in the STED images, we employ a Laplacian filter on the vinculin data to identify the centers of the vinculin patches. We then use thresholding and a connected component analysis to identify the spatial extent of the patches around these points. Sizes and orientations are computed by performing a moment analysis and fitting ellipses to the results. The mathematical details are given in the supporting material.

For statistical comparison of different experimental conditions, we first apply a Kruskall-Wallis rank sum test to all conditions. In cases where the Kruskall-Wallis rank sum test shows a difference on a significance level of 5%, we additionally perform a Dunn's test on all possible pairings, again with a 5% significance level.

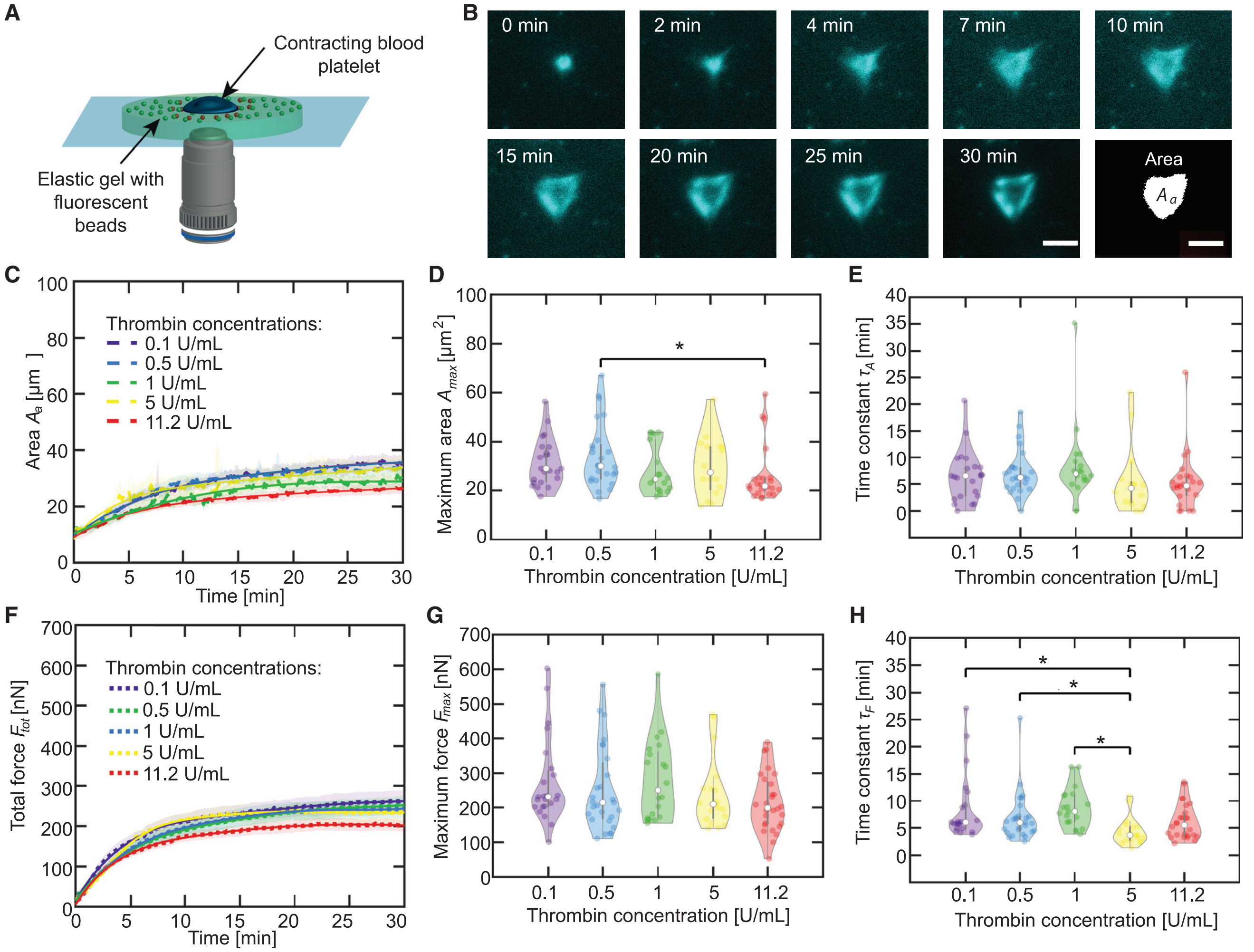

FIGURE 1 Above a threshold, thrombin concentration has no systematic effect on platelet spreading and force generation. (*A*) Schematic representation of the experimental setup. A contracting platelet (blue) on top an elastic gel (light green) displaces the fluorescent beads (green). The deformation of the gel due to the contraction is visualized as orange beads. (*B*) Epifluorescence micrographs of a blood platelet stained for actin and spreading on an elastic gel after stimulation by thrombin. The last image shows a binarized version of the $t = 30$ min micrograph. The scale bars correspond to 5 μm and refer to all subpanels. The example platelet shown here is also shown in Figs. 3, 4 *A*, and 5. (*C*) The average time-dependent total area covered by actin structures $A_a(t)$ upon stimulation by different concentrations of thrombin. Each averaged curve contains a minimum of 18 datasets and the SEs are shown as the transparent areas. Averaged exponential fits are shown as solid lines. (*D*) Maximum areas for each dataset determined from the averaged total $A_a(t)$ curves shown in (*C*). (*E*) Time constants for each dataset determined from the averaged total $A_a(t)$ curves shown in (*C*). (*F*) Average total force curves for blood platelets ($n \geq 18$) upon stimulation by different concentrations of thrombin are shown as dotted lines, and the SEs are included as the transparent areas. Averaged exponential fits are shown as solid lines. (*G*) Maximum forces for each dataset comprising the averaged total force curves shown in (*F*). (*H*) Time constants for each dataset comprising the averaged total force curves shown in (*F*). The violin plot outlines illustrate the kernel probability density; the width of the shaded area represents the proportion of the data points located there. In each violin plot, the white circle indicates the median, the thick gray bar indicates interquartile range, and the thin gray line indicates the full range of distribution without outliers. Experimental conditions with a statistical difference below a 5% significance level are marked by asterisks (*). To see this figure in color, go online.

RESULTS

Effect of thrombin concentration on platelet spreading and force generation

To investigate the spreading speed and force evolution of platelets when activated by different thrombin concentrations between 0.05 and 11.2 U/mL, we record movies of the emerging actin structures and, in parallel, the bead patterns below the platelets, which are then used to calculate the force fields with TFM procedures. All gels are incubated with a fibrinogen solution with a concentration of 100 μg/mL. At a thrombin concentration of 0.05 U/mL, we do not observe spreading, actin structure formation, or force generation (see Fig. S5 in the supporting material), in contrast to the situation at 0.1 U/mL. We thus find that the threshold thrombin concentration for platelet activation lies between 0.05 and 0.1 U/mL. Above this threshold, the actin structures in thrombin-activated platelets dramatically change upon spreading on a fibrinogen-coated gel, as shown for a typical example in Fig. 1 *B* and Video S1. Note that imaging at high magnification is challenging when the optical path encompasses a PAA gel in addition to a glass coverslip. We analyze the increase of the area occupied by actin structures A_a with respect to time, rather than the fluorescence intensity, as it has been shown that the binding kinetics of the SiR-actin probe is slower than the actin polymerization dynamics (26). Fig. 1 *C* shows $A_a(t)$, color coded for the different thrombin concentrations used for platelet activation. The dashed lines denote the data, averaged from a minimum of 18 platelets each, whereas the transparent areas show the SEs. The individual data curves are shown in the supporting material in Fig. S6. For comparison, we fit the data with an exponential growth function,

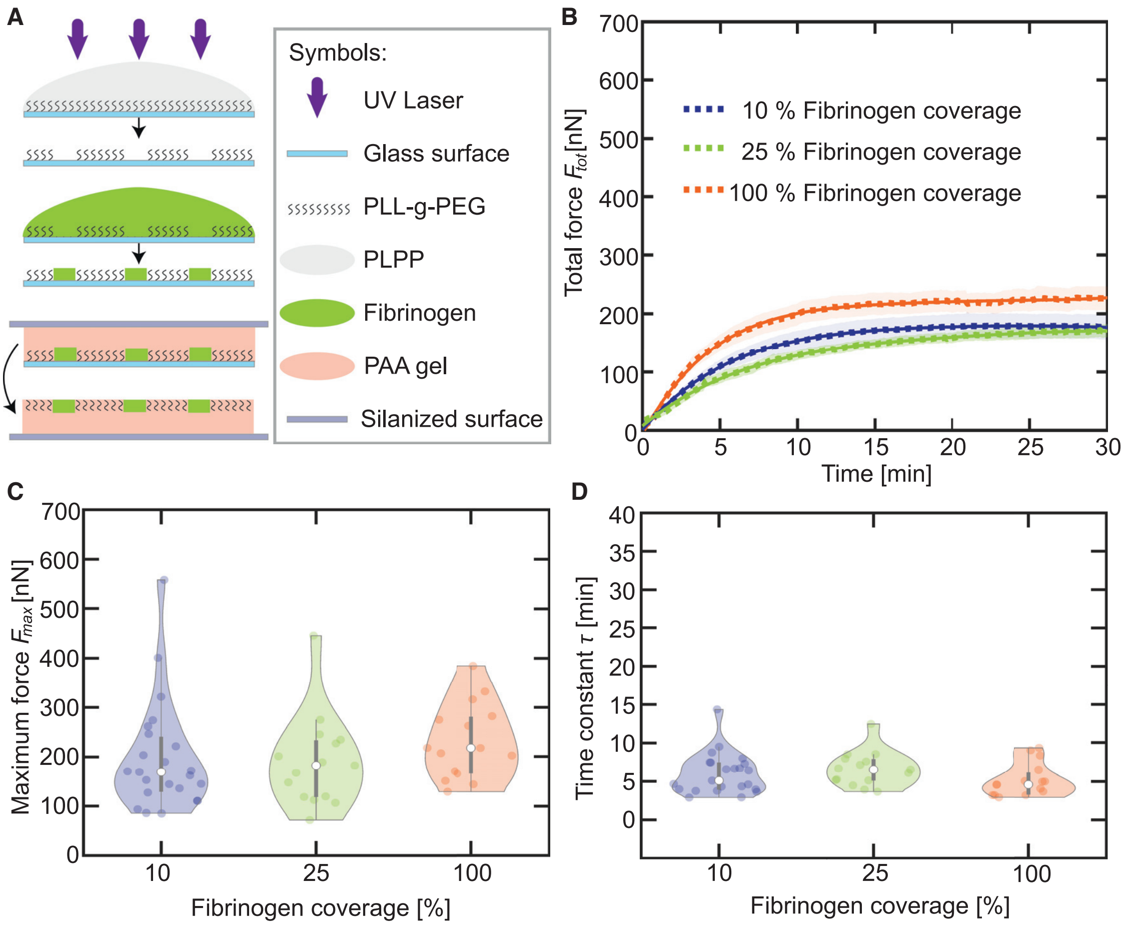

FIGURE 2 Fibrinogen coverage has no effect on force generation. (*A*) Schematic representation of the preparation of micro-patterned gel surfaces with varying fibrinogen coverage (100%, 25%, 10%) by photo patterning (LIMAP). (*B*) Average total force curves for blood platelets ($n \geq 15$) upon stimulation by thrombin (0.1 U/mL) on gels covered by various concentrations of fibrinogen (dotted lines); the SEs are included as the transparent areas. Averaged exponential fits are shown as solid lines. Each dataset comprising the averaged curves is fitted and the maximum forces and time constants are determined. (*C*) Maximum forces for each dataset determined from the averaged total force curves shown in (*B*). (*D*) Time constants for each dataset determined the averaged total force curves shown in (*B*). The violin plot outlines illustrate the kernel probability density; the width of the shaded area represents the proportion of the data points located there. In each violin plot, the white circle indicates the median, the thick gray bar indicates interquartile range, and the thin gray line indicates the full range of distribution without outliers. To see this figure in color, go online.

$$A_a(t) = A_{max} \cdot [1 - B_a \cdot \exp(-t / \tau_A)], \quad (3)$$

where A_{max} is the maximum area, $A_{max}(1 - B_a)$ is the initial detected area, and τ_A is the time constant for the time-resolved area increase. The fit parameters A_{max} and τ_A are shown in Fig. 1 *D* and *E*, respectively. At a significance level of 5%, we observe a statistical difference for the maximum area A_{max} between 0.5 and 11.2 U/mL thrombin, but not between the other conditions and not for the time constant τ_{A} (Kruskall-Wallis rank sum test and Dunn's test).

The total force $F_{tot}(t)$, derived from the traction forces according to Eq. 1 for each thrombin concentration is shown in Fig. 1 *F*. The averaged data are shown as dotted lines and the SEs as transparent areas. The individual data curves are shown in the supporting material in Fig. S7. For a quantitative comparison, we again fit an exponential growth function,

$$F_{tot}(t) = F_{max} \cdot \left[1 - B_f \cdot \exp(-t / \tau_F)\right], \quad (4)$$

where F_{max} is the maximum force, $F_{max}(1 - B_f)$ is the initial force, and τ_F is the time constant. The fit parameters F_{max} and τ_F are shown in Fig. 1 *G* and *H*, respectively. At a significance level of 5%, we observe a statistical difference for the time constant τ_{F} between 5 U/mL and 0.1, 0.5, and 1 U/mL, but not between the other conditions and not for the maximum force F_{max} (Kruskall-Wallis rank sum test and Dunn's test). Thus, our data show that, despite their small size, platelets produce large forces of several hundreds of nanonewtons and that these forces are not systematically dependent of the thrombin concentration. Interestingly, the development of the force magnitude and the actin structures are on the same timescale.

Effect of fibrinogen coverage on platelet spreading and force generation

In a next step, we systematically investigate—similar to the experiments described above—the force evolution in dependence of the fibrinogen coverage. We use a thrombin concentration of 0.1 U/mL just above the threshold determined before and a fibrinogen solution with a concentration of 100 μg/mL. The varying surface coverage (100%, 25%, 10%) is obtained by photo patterning (LIMAP) as shown in Fig. 2 *A*. We again record the substrate deformation and determine the total force $F_{tot}(t)$ for each fibrinogen coverage (see Fig. 2 *B*). The individual data curves are shown in the supporting material in Fig. S8. Each single measurement curve is fitted exponentially according to Eq. 4, and the averaged fit curves are shown as solid lines. The fit parameters F_{max} and τ are shown in Fig. 2 *C* and *D*, respectively, and they show no significant difference (Kruskall-Wallis rank sum test). Imaging the actin structures within platelets spreading on LIMAP-coated surfaces leads to high background signal and we are thus not able to determine the temporal development of the spread area. However, Fig. S9 in the supporting material shows a comparison of the contraction forces on fibrinogen-coated gels and on 100% photo patterned gels and they agree well,

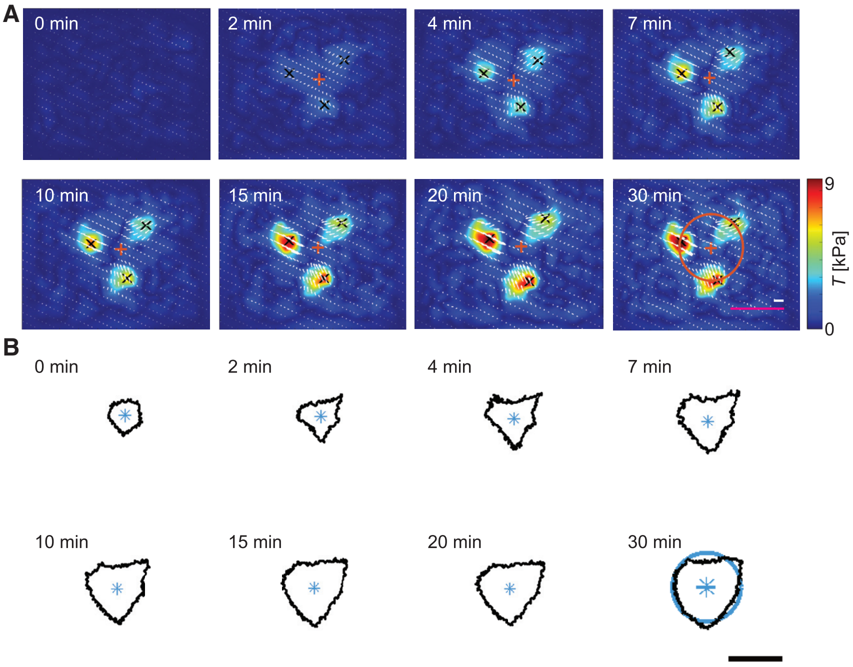

FIGURE 3 Time-resolved traction forces reveal emergence of force hotspots and show same geometry as cell shape. (*A*) Image sequence showing the temporal evolution of the force distribution. Black crosses represent the centers of the force hotspots and white arrows indicate the direction of the force field. For each image, the centroid of the hotspots is calculated and is represented as an orange cross. As an example, the orange circle shown on the $t = 30$ min image has a radius equal to the mean radial distance of the force (MRD_f) between the centroid and the black crosses. Corresponding MRD_f are calculated for each time point of the video and each individual platelet. The magenta scale bar corresponds to 5 μm, the white scale bar corresponds to 5 kPa, and they refer to all sub-panels. (*B*) Image sequence showing the time evolution of the actin boundary corresponding to the same time points shown in (*A*). For each image, the centroid is represented by a blue star. As an example, the blue circle shown in the $t = 30$ min image has a radius equal to the mean radial distance (MRD_a) between the centroid and the actin boundary. Corresponding MRD_a are calculated for each time point of the video and each individual platelet. The scale bar corresponds to 5 μm and refers to all sub-panels. The example platelet shown here is also shown in Figs. 1 *B*, 4 *A*, and 5. To see this figure in color, go online.

underlining the validity of our method. Taken together, our results show that neither the thrombin concentration in the platelet suspension nor the fibrinogen coverage on the substrate influence the force generation in a systematic manner.

Spatial correlation of force fields and cell shape in spreading platelets

To better understand the correlation between force generation and the cell shape, in Fig. 3 *A* and *B* we show the traction fields and cell contours, respectively, for a typical time series. We observe that both processes show the same triangular geometry. The white arrows indicate the force vectors generated by the platelet. This force is mostly concentrated at the three corners of the triangular cell in force hotspots (32). We further observe that they point toward the geometrical center. We note that the internal forces are likely higher than the values we record here (see Figs. 1 *F* and 2 *B*), because TFM can only measure the vectorial sum of the forces acting through the adhesions onto the substrate. The overlay of the actin boundary and the force map is shown as Video S2, the bead patterns used to determine the force map are shown in Video S3.

To quantify the spatial and temporal correlation of the force fields and cell shape, we first determine the position of the force hotspots using the MATLAB function "Fast 2D peak finder" (41). Here, we focus on those peaks that exceed a level of 50% of the highest traction force at each time point. For each platelet, we determine the centroid of these peaks $C_f(x_{cf}, y_{cf})$ (see orange crosses in Fig. 3 *A*) and the mean radial distance of the force MRD_f, defined as the average Euclidean distance between the centroid C_f and the detected hotspots:

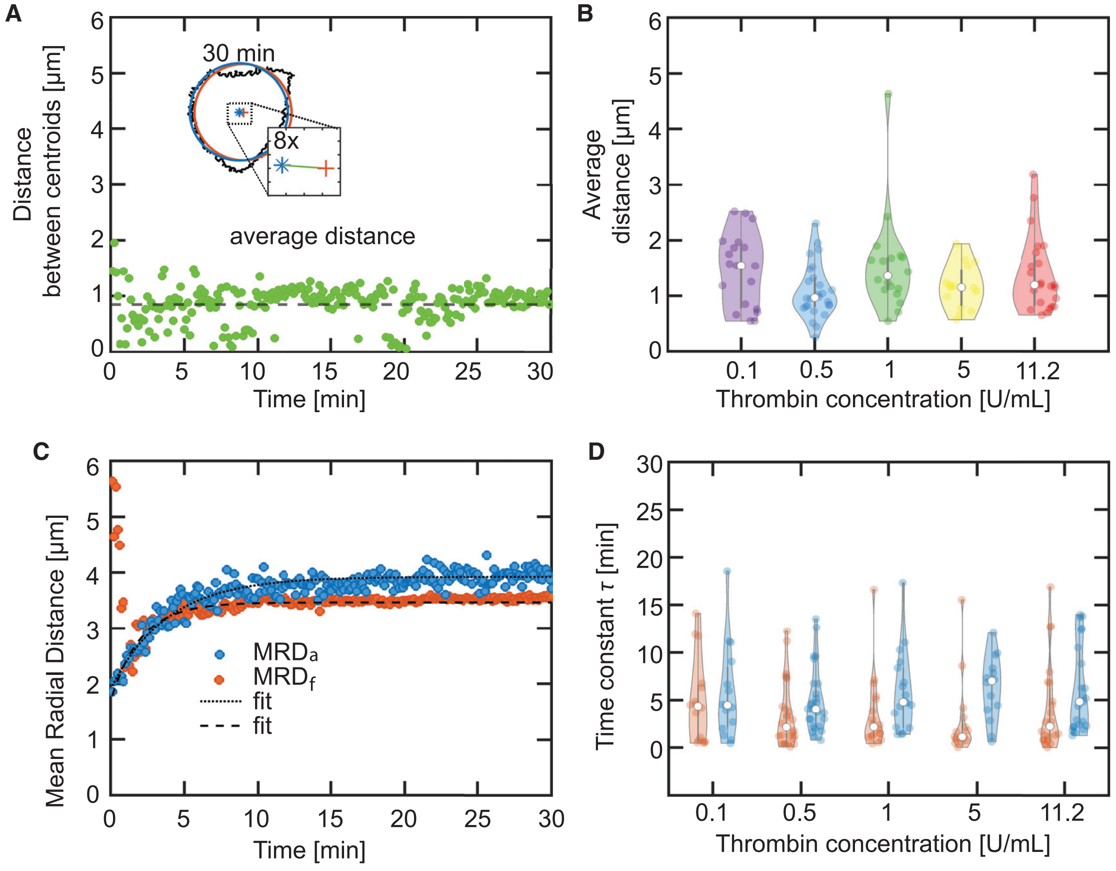

FIGURE 4 The centroids of the force hotspots and the actin boundary collapse with time. (*A*) The inset shows an overlay of the actin boundary (black), the MRD_a circle (blue), and the MRD_f circle (orange) for the $t = 30$ min image. The distance between the centroids of the two circles is shown in the zoom-in and is represented by the green line. The distance between the centroids is calculated for each time point and is plotted in a time-resolved manner. The average distance is represented as the dashed black line. This analysis is repeated for every platelet used in the experiments with differing thrombin concentrations. The example platelet shown here is also shown in Figs. 1 *B*, 3 *A*, and 5. (*B*) Distribution of the average distance between MRD_a and MRD_f circles for each thrombin concentration. (*C*) Temporal evolution of the MRD_a (blue) and MRD_f (orange) for the example shown in Fig. 3 and (*A*). (*D*) Distribution of the time constant for the radial evolution for each MRD_a circle (blue violin plots) and MRD_f circle (orange violin plots). See axis labels for different conditions. The violin plot outlines illustrate the kernel probability density; the width of the shaded area represents the proportion of the data points located there. In each violin plot, the white circle indicates the median, the thick gray bar indicates interquartile range, and the thin gray line indicates the full range of distribution without outliers. To see this figure in color, go online.

$$\mathrm{MRD}_f = \frac{1}{K_f} \cdot \sum_{k=1}^{K_f} \sqrt{\left(x_{cf} - x_f(k)\right)^2 + \left(y_{cf} - y_f(k)\right)^2}, \quad (5)$$

where K_f is number of detected peaks and (x_f, y_f) are coordinates of the detected peaks. Second, we determine the centroid of the actin boundary $C_a(x_{ca}, y_{ca})$ (see blue stars in Fig. 3 *B*) at each time point. Additionally, we introduce a variable for the mean radial distance of the actin area MRD_a, defined as the average Euclidean distance between the centroid C_a and the actin boundary:

$$\mathrm{MRD}_a = \frac{1}{K_a} \cdot \sum_{k=1}^{K_a} \sqrt{(x_{ca} - x_a(k))^2 + (y_{ca} - y_a(k))^2}, \quad (6)$$

where K_a is number of pixels on the actin boundary and (x_a, y_a) are the coordinates of the pixels. In Fig. 4 *A*, we show a typical example of the time-dependent distance between the centroid of the force hotspots C_f and centroid of the actin boundary C_a. The averaged value is indicated by the black dashed line. For each measurement, we determine the time-averaged distance, shown in Fig. 4 *B*. The results show no significant difference (Kruskall-Wallis rank sum test, $p > 0.05$) between the thrombin concentrations. These results show that the cell as a whole arrives relatively fast at its final shape and an equilibrium of mutually balancing forces, which effectively are centered on the centroid. Thus, shape and force fields nicely correspond to each other.

In Fig. 4 *C* we present an example of $\mathrm{MRD}_a(t)$ and $\mathrm{MRD}_f(t)$ of one typical platelet. It is evident that both curves show a very similar exponential increase. We quantify this trend by fitting the curves with an exponential model:

$$\mathrm{MRD}(t) = \mathrm{MRD}_{max} \cdot [1 - B_{\mathrm{MRD}} \cdot \exp(-(t+d)/\tau_{\mathrm{MRD}})], \quad (7)$$

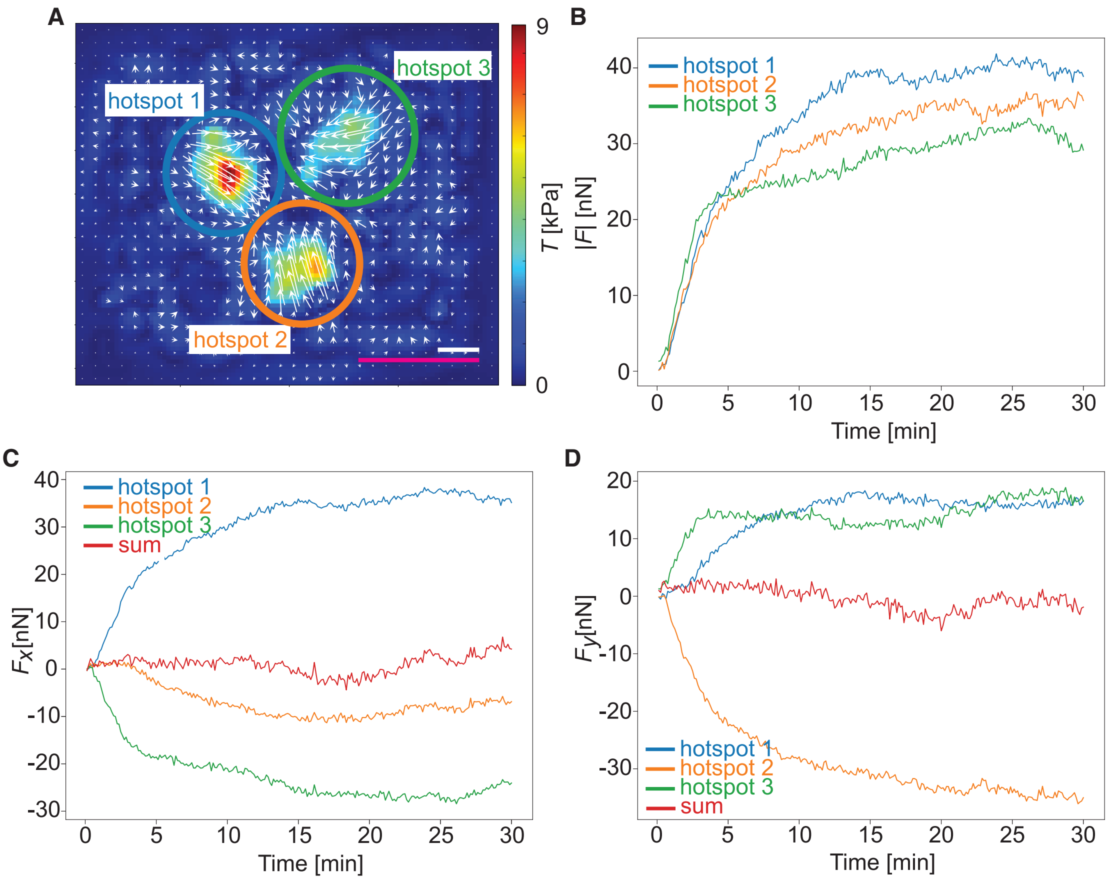

FIGURE 5 The force hotspots form a balanced system of strongly localized forces. (*A*) Reconstructed tangential traction forces at the final time point of spreading for the cell presented in Fig. 3 with three manually defined adhesion hotspot areas indicated by blue, green, and orange circles. The color coding is valid for all subfigures. The magenta scale bar corresponds to 5 μm and white scale bar corresponds to 5 kPa. (*B*) Time evolution of the magnitude of the traction force vectors obtained by integrating the traction vector field in each individually marked adhesion area. (*C*) Time evolution of the *x* components of the traction force vectors for the each individually marked adhesion area. The red curve shows sum of all three components. (*D*) The time evolution of the *y* components of the traction force vectors for each individually marked adhesion area. The red curve shows sum of all three components. The example platelet shown here is also shown in Figs. 1 *B*, 3, and 4 *A*. To see this figure in color, go online.

where MRD_{max} is the maximum MRD_a or MRD_f, d is the time offset, and τ is the time constant. We determine the time constants as plotted in Fig. 4 *D*. In agreement with all previous results, τ_{MRD_a} and τ_{MRD_f} also show no significant difference between the measured conditions (Kruskall-Wallis rank sum test, $p > 0.05$). However, when comparing the medians and interquartile ranges (IQRs) of τ_{MRD_a} ($n = 104$, 5.2 $\pm$ 3.9 min) and τ_{MRD_f} ($n = 104$, 3.8 $\pm$ 3.4 min) for all conditions, we find that τ_{MRD_f} is significantly faster (Wilcoxon rank sum test, $p < 0.05$). Thus, the distribution of force hotspots is equilibrated early on, whereas the platelet shape, as given by the area covered by actin structures, is still developing further.

In Fig. 5, we analyze the force balance of one adherent platelet. We calculate the force vectors for the three hotspots, which are all directed toward a point close to the centroid of the cell with a strength between 20 and 40 nN each at the final time point of spreading (Fig. 5 *A* and *B*). These traces confirm the time constant for the increase in contractility concluded from the evolution of total force in Fig. 2 *B*. As shown in Fig. 5 *C* and *D*, the x and y components of the three hotspot forces nicely add up to zero, thus confirming force balance and the high resolution of our measurements. Overall we conclude that this system is a geometrically simple system of strongly localized and balanced forces.

Spatial correlation of force fields and subcellular structures in spreading platelets

Imaging of the cytoskeletal structures through the PAA gel is challenging and does not give the same resolution as fixed and stained samples (compare the examples in Fig. 1 *B* and Video S1). Therefore, to complement the dynamic data we record during the force measurements, we chemically fix platelets at the end of the spreading process and label actin (SiR-actin) and vinculin (immunostaining). We then embed the samples and perform superresolution STED microscopy through the glass slide on top of the platelets. In Fig. 6 *A*, we show representative examples of the images obtained by this procedure (actin in cyan and vinculin in magenta). We find that the triangular pattern shown in Figs. 3 and 5 is very common (compare Fig. 6 *A*.iii) but that other geometrical patterns also occur, namely spindles (Fig. 6 *A*.i), squares (Fig. 6 *A*.ii), polygonal (Fig. 6 *A*.iv), and circular shapes (Fig. 6 *A*.v). In Fig. 6 *B* we show the corresponding force maps recorded at the end of the spreading process, just before fixation and STED imaging, where the white arrows indicate the direction of the force field and showing areas with concentrated force hotspots at the edges of the platelets. Again, shapes and force fields show similar geometries. In Fig. 6 *C* we finally show the time courses leading to these force fields. Despite the very different cell organizations, these

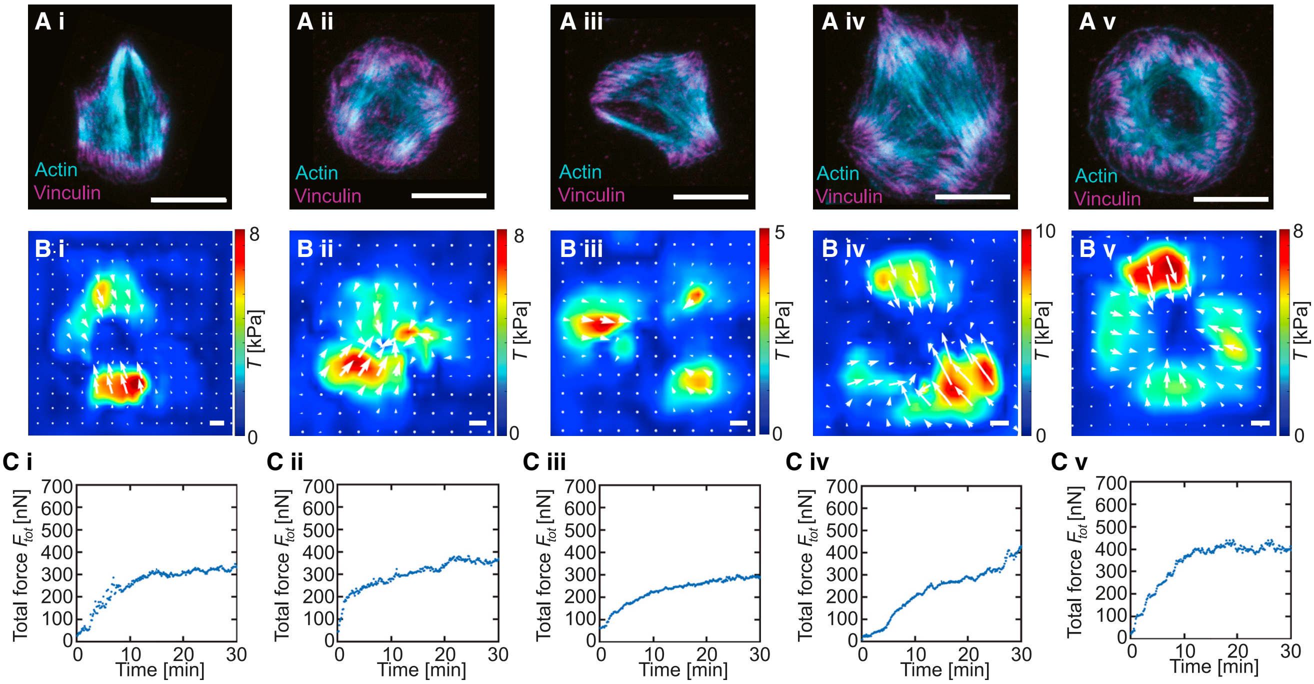

FIGURE 6 STED imaging of fixed and stained cells reveals details of cell organization during force generation. Typical examples of i) spindle-like, ii) square, iii) triangular, iv) polygonal, and v) circular platelets. (*A*) Superresolution STED imaging; actin in cyan and vinculin in magenta; the white scale bars correspond to 5 μm. (*B*) Traction forces before fixation with the white arrows indicating the direction of the force fields; the white scale bars correspond to 5 kPa. (*C*) Time evolution of the total force produced by the cell before fixation. See Fig. S10 in the supporting material for five additional examples. To see this figure in color, go online.

time courses and the plateau levels for the forces are very similar.

In the STED images from Fig. 6 *A*, actin in cyan represents intracellular force generation, whereas vinculin in magenta represents adhesion and force transmission to the substrate. To contract the extracellular environment, these two systems have to work together. To analyze this aspect in quantitative detail, we turn to a pairwise correlation between vinculin intensity distribution, actin stress fiber orientation, and force fields. In Fig. 7 *A*, we show the radially integrated vinculin intensity (23) (red crosses and solid fit curves), indicative of the positions of the focal adhesions, and the radially integrated force magnitude (blue). In cells i to iv, the maxima of the curves overlay strongly, demonstrating a pronounced angular correlation of the traction forces and the focal adhesions. The circular cell (v) is an exception to this observation, as in this case, the vinculin is almost evenly distributed over all angles. In Fig. 7 *B*, we focus on the stress fibers, which are segmented (cyan) and shown together with the vinculin signal, which has been processed with thresholding and a moment analysis to indicate patch sizes and orientations (magenta ellipses). We see that the ends of the stress fibers usually correspond to vinculin patches and that their orientations tend to co-align. We note that the converse is not necessarily true, because there are also many vinculin spots that do not correspond to the endpoints of stress fibers. We also see that the circular cell is special because its stress fibers are arranged in a circular fashion and do not cross the interior of the cell; nevertheless, here too we see alignment between stress fibers and vinculin patches. Finally, Fig. 7 *C* shows that the forces at the ends of stress fibers correlate with the directions of the force field. This correlation is most obvious in the case of the spindle-shaped cell (i), when we have only one family of stress fibers. In the other cases, stress fibers of different orientations meet at one force hotspot, and then it seems to be the vectorial sum of these stress fibers that aligns with the force vectors. Again, this reflects the fact that TFM can only measure the vectorial sum of the forces transmitted through the adhesion.

Taken together, STED microscopy through glass cover slips enables us to resolve the adhesions and stress fibers in the platelets much more clearly than when using live-cell imaging through PAA gels. Notably, through the procedures established here, we are now able to combine the high resolution of STED microscopy with the dynamic traction force measurements by imaging the endpoint of these dynamics. For all 10 cells analyzed (five in Figs. 6 and 7; five more in Figs. S10 and S11 in the supporting material), we observe that traction hotspots align with the endpoints of actin stress fibers, which are anchored in focal adhesions, as shown by the vinculin immunostaining. The force arrows and the focal adhesions tend to be co-aligned, although the segmentations are not sufficient to perform a more detailed analysis. Overall, our data provide strong indication

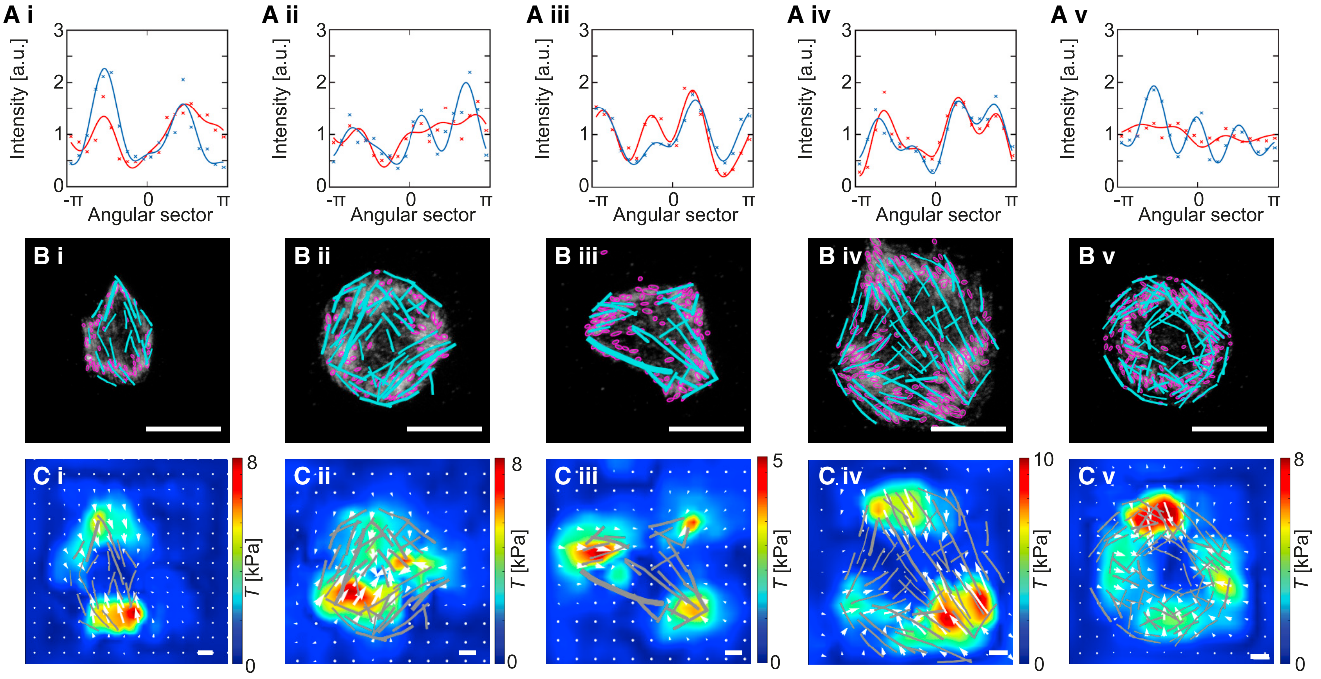

FIGURE 7 Actin structures, vinculin patterns, and force fields are correlated. The same cells (i–v) as in Fig. 6 are shown. (*A*) Circumferential profiles of the vinculin signal (red) and force magnitude (blue); the crosses show the data, the solid lines are fits. (*B*) Overlay of segmented vinculin spots (focal adhesions; magenta) and stress fibers (cyan); the white scale bars correspond to 5 μm. (*C*) Overlay of the traction forces field and the segmented stress fibers (gray); the white scale bars correspond to 5 kPa. See Fig. S11 in the supporting material for five additional examples. To see this figure in color, go online.

that the stress fibers ending in the focal adhesions indeed are the force-generating elements in platelets.

DISCUSSION

Due to the challenge of simultaneously measuring forces and imaging the cytoskeleton of the small and nucleus-free platelets, it is still not clear how exactly cytoskeletal organization and force generation correspond to each other. Here we directly connect force generation and cytoskeletal structure formation by combining our earlier advances in in situ actin imaging (26) and TFM (32). The lack of a nucleus in platelets leads us to use SiR-actin as a probe to visualize the formation of stress fibers in human platelets and we reveal similar structures as have been observed by immunostaining of chemically fixed platelets (22–24) as well as in time-resolved experiments with spreading platelets on stiff glass substrates (26). We confirm our new findings by super-resolution STED microscopy, which reveals even more details but requires fixation. For TFM, we use state-of-the-art methods to achieve high spatial and temporal resolution, which is essential to investigate the relation between force field evolution and the temporal development of cytoskeletal structures. In agreement with our earlier work (32), we find that the maximum total force of few hundreds of nanonewtons is generated within 30 min and with the average time constants for the force increase and area increase 6.0 ± 2.9 min and 5.5 ± 3.6, respectively. This observation is also in a good agreement with the observed time constant for actin structure formation on glass (4.1 ± 2.4 min) (26).

Because of the limited resolution of our live-cell imaging, in particular for the early time points, we analyze the overall substrate coverage by actin, which corresponds to the spreading area, rather than individual fibrous actin structures. The limitations in resolution are caused by the slow binding kinetics of the SiR-actin probe (25) as well as by the fact that the imaging is performed though a relatively thick PAA gel with an increasing background by unbound dyes. Our results confirm that platelets produce the largest traction forces in force hotspots (32). We are able to spatially and temporarily correlate these hotspots with the endpoints of straight actin structures, which we interpret as force-generating actin stress fibers. Consistent with our results reported here, a recent study found that at a high-level tension regime integrins are focused at two to three spots at the periphery after 4 min (42). Interestingly, we observe a significantly slower time constant (Wilcoxon rank sum test, $p < 0.5$) for the spatial development of actin structures compared to the time constant for the positions of the hotspots. This indicates that the hotspots are spatially stabilized before the actin structures are fully rearranged. This could indicate that the spatial force distribution is organized at early time points of the actin polymerization; at a later stage, the acto-myosin interactions increase the force and stabilize the contraction.

Our approach of combining live-cell imaging with time-resolved TFM and STED imaging of the final actin/vinculin structures provides a consistent picture of force generation in platelets. The actin stress fibers, which can be segmented well in the STED images, are anchored in focal adhesions, which we visualize by vinculin staining, and correlate in direction with the measured forces, with the caveat that, when different stress fibers are anchored to the same adhesion, their forces seem to add up. The shape of each platelet is mostly defined by these stress fibers, which lead to simple geometric shapes, such as spindles or triangles. Additional vinculin structures, and therefore adhesion points, are found in the regions between the endpoints of the stress fibers, where they might serve as anchor points for lamellipodia and other intracellular structures. When analyzing the force fields that the platelets exert on the elastic substrate, we observe hotspots, which emerge early in the spreading process, are colocalized with the vinculin staining in the focal adhesions, remain spatially stable over time, and increase in force magnitude, presumably through growth of stress fibers. Thus, over a time course of about 30 min, a total force per platelet of several hundreds of nanonewtons is reached.

Neither the total force, nor the time constant for force generation, nor the actin area or the correlation of actin structures and forces fields are systematically influenced by varying concentrations of thrombin added to the platelets suspension as long as the threshold concentration for activation by thrombin is ensured. In agreement with earlier results by Myers et al. (30) for stiffness values of 25 or 50 kPa, which are closest to our value of 34 kPa, the effect of thrombin is indeed not very pronounced and may be negligible given the platelet-to-platelet variation we encounter in our experiment. We might have obtained different results for lower stiffness, as reported earlier (30), but here we focus on a physiologically relevant stiffness value (35). Our measured total platelet forces, integrated over the total platelet spread area, are higher than the forces measured by Myers et al. (30), likely because, in that work, only the 1D force exerted on two dots is determined, whereas we take the full platelet force field into account. Because these forces are strongly localized, we also expect that platelets in physiological 3D environments exert forces that are larger and more localized than formerly appreciated. To prove this expectation, in the future one would have to develop methods for 3D-TFM that allow for spatially resolving the forces of the very small platelets, such as using 3D-printed structured environments (43).We also find that the total force is not influenced by the fibrinogen coverage on the substrate. Biologically, this could indicate that platelet contraction, and possibly function in general, is a very robust process that is not easily influenced by external parameters once

activated. In the future, it would be interesting to also study other pathways to platelet activation, such as ADP or serotonin (6), and different substrate coatings, such as fibronectin and collagen. In contrast to our results, a recent study shows that an increasing thrombin concentration increases platelet forces in microthrombi (28). Hence, our results on a single platelet may indicate that the force increase in microthrombi is more likely connected to the cell-cell interaction or to the tension of fibrin fibers in the larger cluster of cells. It is not clear yet if all platelets within a microthrombus actively contribute to the force increase and how. Therefore, further research on small ensembles of platelets is needed to validate this hypothesis. An investigation of their adhesion and dynamics of the forces could provide information in this regard.

Taken together, our study demonstrates a close relation between filamentous actomyosin structures and force generation in healthy blood platelets. Our approach has the potential to better understand in the future how bleeding disorders such as Glanzmann's thrombasthenia might arise because they disturb the relation between formation of actin structures and force generation.

SUPPORTING MATERIAL

Supporting material can be found online at https://doi.org/10.1016/j.bpj.2023.07.010.

AUTHOR CONTRIBUTIONS

S.K. and U.S.S. designed research and supervised this work. A.Z. performed research. J.B. and D.P. contributed data analysis tools. R.G. and G.L. supported the superresolution experiments. J.B. and A.Z. analyzed data. A.Z. and S.K. wrote the original draft of the paper. A.Z., J.B., U.S.S., and S.K. worked on the final version. All authors reviewed and approved the paper.

ACKNOWLEDGMENTS

We thank the University Medical Center Göttingen, in particular Alexander Strate, Tobias Legler, and Joachim Riggert, for providing platelet concentrates; Jana Hanke and Anna Blob for technical support; and Axel Munk, Charlotta Lorenz, and Florian Rehfeldt for fruitful discussions. This work was funded by the German Research Foundation (DFG) in the framework of SFB 937, project A12 (to S.K.) and under Germany's Excellence Strategy (EXC 2067/1-390729940 to S.K. and EXC 2082/1-390761711 to U.S.S.). U.S.S. and S.K. also acknowledge support by the Max Planck School Matter to Life. J.B. thanks the Carl Zeiss Foundation for financial support. The work was further financially supported by the European Research Council (ERC, grant no. CoG 724932, to S.K.). U.S.S. is a member of the Interdisciplinary Center for Scientific Computing (IWR) at Heidelberg.

DECLARATION OF INTERESTS

The authors declare no competing interests.

REFERENCES

1. A. Michelson, M. Cattaneo, and …, P. Newmaneds 2019. Platelets, Fourth edition Academic Press.
2. Patel-Hett, S., J. L. Richardson, …, J. E. Italiano. 2008. Visualization of microtubule growth in living platelets reveals a dynamic marginal band with multiple microtubules. *Blood.* 111:4605–4616. https://doi.org/10.1182/blood-2007-10-118844.
3. Cerecedo, D., I. Martínez-Vieyra, …, I. J. Galván. 2013. Haemostatic role of intermediate filaments in adhered platelets: Importance of the membranous system stability. *J. Cell. Biochem.* 114:2050–2060. https://doi.org/10.1002/jcb.24546.
4. Diagouraga, B., A. Grichine, …, K. Sadoul. 2014. Motor-driven marginal band coiling promotes cell shape change during platelet activation. *J. Cell Biol.* 204:177–185. https://doi.org/10.1083/jcb.201306085.
5. Niederman, R., and T. D. Pollard. 1975. Human platelet myosin. II. In vitro assembly and structure of myosin filaments. *J. Cell Biol.* 67:72–92. https://doi.org/10.1083/jcb.67.1.72.
6. Blockmans, D., H. Deckmyn, and J. Vermylen. 1995. Platelet activation. *Blood Rev.* 4:1–4. https://pubmed.ncbi.nlm.nih.gov/8563516/.
7. Holme, P. A., U. Ørvim, …, K. S. Sakariassen. 1997. Shear-induced platelet activation and platelet microparticle formation at blood flow conditions as in arteries with a severe stenosis. *Arterioscler. Thromb. Vasc. Biol.* 17:646–653. https://doi.org/10.1161/01.atv.17.4.646.
8. Litvinov, R. I., O. V. Gorkun, …, J. W. Weisel. 2007. Polymerization of fibrin: Direct observation and quantification of individual B:b knob-hole interactions. *Blood.* 109:130–138. https://doi.org/10.1182/blood-2006-07-033910.
9. Jennings, L. K. 2009. Mechanisms of platelet activation: Need for new strategies to protect against platelet-mediated atherothrombosis. *Thromb. Haemostasis.* 102:248–257. https://doi.org/10.1160/th09-03-0192.
10. Thomas, M. R., and R. F. Storey. 2015. The role of platelets in inflammation. *Thromb. Haemostasis.* 114:449–458. https://doi.org/10.1160/th14-12-1067.
11. Johnson, G. J., L. A. Leis, …, J. G. White. 2007. The critical role of myosin IIA in platelet internal contraction. *J. Thromb. Haemostasis.* 5:1516–1529. https://doi.org/10.1111/j.1538-7836.2007.02611.x.
12. Sadoul, K. 2015. New explanations for old observations: Marginal band coiling during platelet activation. *J. Thromb. Haemostasis.* 13:333–346. https://doi.org/10.1111/jth.12819.
13. Dmitrieff, S., A. Alsina, …, F. J. Nédélec. 2017. Balance of microtubule stiffness and cortical tension determines the size of blood cells with marginal band across species. *Proc. Natl. Acad. Sci. USA.* 114:4418–4423. https://doi.org/10.1073/pnas.1618041114.
14. Aquino, D., A. Schönle, …, A. Egner. 2011. Two-color nanoscopy of three-dimensional volumes by 4Pi detection of stochastically switched fluorophores. *Nat. Methods.* 8:353–359. https://doi.org/10.1038/nmeth.1583.
15. Hartwig, J. H. 1992. Mechanisms of actin rearrangements mediating platelet activation. *J. Cell Biol.* 118:1421–1442. https://doi.org/10.1083/jcb.118.6.1421.
16. Hartwig, J. H., G. M. Bokoch, …, T. P. Stossel. 1995. Thrombin receptor ligation and activated rac uncap actin filament barbed ends through phosphoinositide synthesis in permeabilized human platelets. *Cell.* 82:643–653. https://doi.org/10.1016/0092-8674(95)90036-5.
17. Hartwig, J. H., S. Kung, …, A. Toker. 1996. D3 phosphoinositides and outside-in integrin signaling by glycoprotein IIb-IIIa mediate platelet actin assembly and filopodial extension induced by phorbol 12-myristate 13-acetate. *J. Biol. Chem.* 271:32986–32993. https://doi.org/10.1074/jbc.271.51.32986.
18. Hartwig, J. H. 2006. The platelet: Form and function. *Semin. Hematol.* 43:S94–S100. https://doi.org/10.1053/j.seminhematol.2005.11.004.
19. Sandmann, R., and S. Köster. 2016. Topographic cues reveal two distinct spreading mechanisms in blood platelets. *Sci. Rep.* 6:22357. https://doi.org/10.1038/srep22357.

20. Horev, M. B., Y. Zabary, …, B. Geiger. 2020. Differential dynamics of early stages of platelet adhesion and spreading on collagen IV-and fibrinogen-coated surfaces. *F1000Research.* 9. https://doi.org/10.12688/f1000research.23598.2.

21. Calaminus, S. D. J., J. M. Auger, …, S. P. Watson. 2007. MyosinIIa contractility is required for maintenance of platelet structure during spreading on collagen and contributes to thrombus stability. *J. Thromb. Haemostasis.* 5:2136–2145. https://doi.org/10.1111/j.1538-7836.2007.02696.x.

22. Poulter, N. S., A. Y. Pollitt, …, S. G. Thomas. 2015. Platelet actin nodules are podosome-like structures dependent on Wiskott–Aldrich syndrome protein and ARP2/3 complex. *Nat. Commun.* 6:7254. https://doi.org/10.1038/ncomms8254.

23. Lickert, S., S. Sorrentino, …, I. Schoen. 2018. Morphometric analysis of spread platelets identifies integrin αIIb β3-specific contractile phenotype. *Sci. Rep.* 8:5428. https://doi.org/10.1038/s41598-018-23684-w.

24. Calaminus, S. D. J., S. Thomas, …, S. P. Watson. 2008. Identification of a novel, actin-rich structure, the actin nodule, in the early stages of platelet spreading. *J. Thromb. Haemostasis.* 6:1944–1952. https://doi.org/10.1111/j.1538-7836.2008.03141.x.

25. Lukinavičius, G., L. Reymond, …, K. Johnsson. 2014. Fluorogenic probes for live-cell imaging of the cytoskeleton. *Nat. Methods.* 11:731–733. https://doi.org/10.1038/nmeth.2972.

26. Paknikar, A. K., B. Eltzner, and S. Köster. 2019. Direct characterization of cytoskeletal reorganization during blood platelet spreading. *Prog. Biophys. Mol. Biol.* 144:166–176. https://doi.org/10.1016/j.pbiomolbio.2018.05.001.

27. Jen, C. J., and L. V. McIntire. 1982. The structural properties and contractile force of a clot. *Cell Motil.* 2:445–455. https://doi.org/10.1002/cm.970020504.

28. Liang, X. M., S. J. Han, …, N. J. Sniadecki. 2010. Platelet retraction force measurements using flexible post force sensors. *Lab Chip.* 10:991–998. https://doi.org/10.1039/b918719g.

29. Lam, W. A., O. Chaudhuri, …, D. A. Fletcher. 2011. Mechanics and contraction dynamics of single platelets and implications for clot stiffening. *Nat. Mater.* 10:61–66. https://doi.org/10.1038/nmat2903.

30. Myers, D. R., Y. Qiu, …, W. A. Lam. 2017. Single-platelet nanomechanics measured by high-throughput cytometry. *Nat. Mater.* 16:230–235.

31. Schwarz Henriques, S., R. Sandmann, …, S. Köster. 2012. Force field evolution during human blood platelet activation. *J. Cell Sci.* 125:3914–3920. https://doi.org/10.1242/jcs.108126.

32. Hanke, J., D. Probst, …, S. Köster. 2018. Dynamics of force generation by spreading platelets. *Soft Matter.* 14:6571–6581. https://doi.org/10.1039/c8sm00895g.

33. Zelená, A., S. Isbaner, …, S. Köster. 2020. Time-resolved MIET measurements of blood platelet spreading and adhesion. *Nanoscale.* 12:21306–21315. https://doi.org/10.1039/d0nr05611a.

34. Vignaud, T., H. Ennomani, and M. Théry. 2014. Chapter 6 - polyacrylamide hydrogel micropatterning. *In* Micropatterning in Cell Biology Part B. M. Piel and M. Théry, eds Academic Press, pp. 93–116, Volume 120 of Methods in Cell Biology. https://www.sciencedirect.com/science/article/pii/B9780124171367000069.

35. Lundkvist, A., E. Lilleodden, …, M. Balooch. 1996. Viscoelastic properties of healthy human artery measured in saline solution by AFM-based indentation technique. *MRS Proc.* 436, 353. https://doi.org/10.1557/proc-436-353.

36. Melero, C., A. Kolmogorova, …, C. Ballestrem. 2019. Light-induced molecular adsorption of proteins using the PRIMO system for micropatterning to study cell responses to extracellular matrix proteins. *J. Vis. Exp.* https://doi.org/10.3791/60092.

37. Schindelin, J., I. Arganda-Carreras, …, A. Cardona. 2012. Fiji: An open-source platform for biological-image analysis. *Nat. Methods.* 9:676–682. https://doi.org/10.1038/nmeth.2019.

38. The MathWorks Inc.. 2020. MATLAB Version: 9.8.0.1359463 (R2020a). The MathWorks Inc..

39. Kittler, J., and J. Illingworth. 1986. Minimum error thresholding. *Pattern Recogn.* 19:41–47. https://doi.org/10.1016/0031-3203(86)90030-0.

40. Hauke, L., A. Primeßnig, …, F. Rehfeldt. 2023. FilamentSensor 2.0: An open-source modular toolbox for 2D/3D cytoskeletal filament tracking. *PLoS One.* 18:1–21. https://doi.org/10.1371/journal.pone.0279336.

41. Natan 2021. Fast 2D peak finder, MATLAB Central File Exchange. https://www.mathworks.com/matlabcentral/fileexchange/37388-fast-2d-peak-finder.

42. Wang, Y., D. N. LeVine, …, X. Wang. 2018. Force-activatable biosensor enables single platelet force mapping directly by fluorescence imaging. *Biosens. Bioelectron.* 100:192–200. https://doi.org/10.1016/j.bios.2017.09.007.

43. Link, R., K. Weißenbruch, …, U. S. Schwarz. 2023. Cell shape and forces in elastic and structured environments: From single cells to organoids. *Adv. Funct. Mater.* 2302145. https://doi.org/10.1002/adfm.202302145.

Biophysical Journal, Volume *122*

Supplemental information

Force generation in human blood platelets by filamentous actomyosin structures

Anna Zelená, Johannes Blumberg, Dimitri Probst, Rūta Gerasimaitė, Gražvydas Lukinavičius, Ulrich S. Schwarz, and Sarah Köster

Supplementary figures

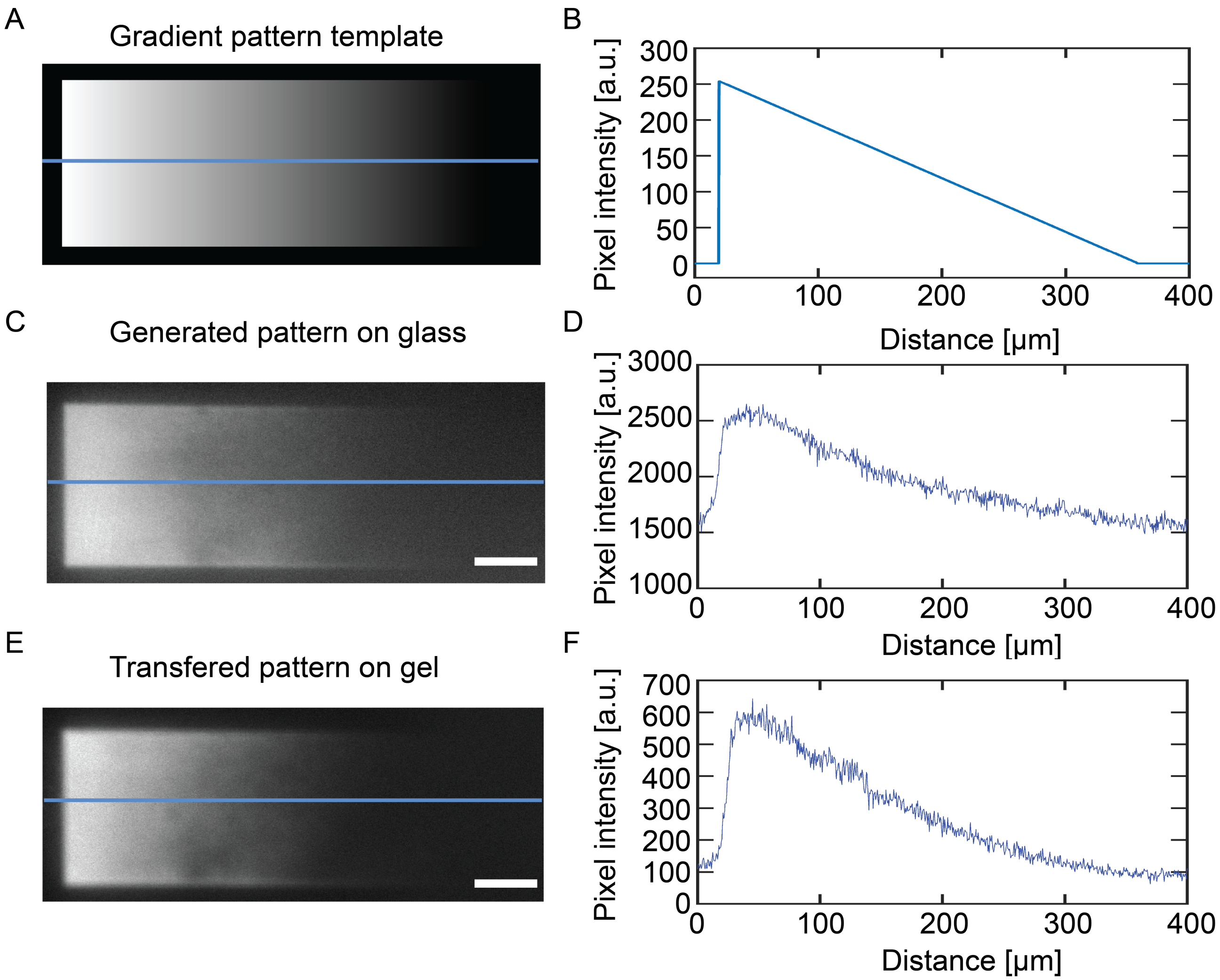

Fig. S1 (A) Template pattern in gray scale. The blue line marks the line scan shown in (B). (C, D) Corresponding gradient pattern generated with LIMAP on glass after incubation with 100 μg/mL fluorescently labeled fibrinogen using a laser power of 2000 mJ/mm^2. (E, F) Corresponding gradient pattern obtained after transfer to the PAA gel. Scale bars correspond to 50 μm.

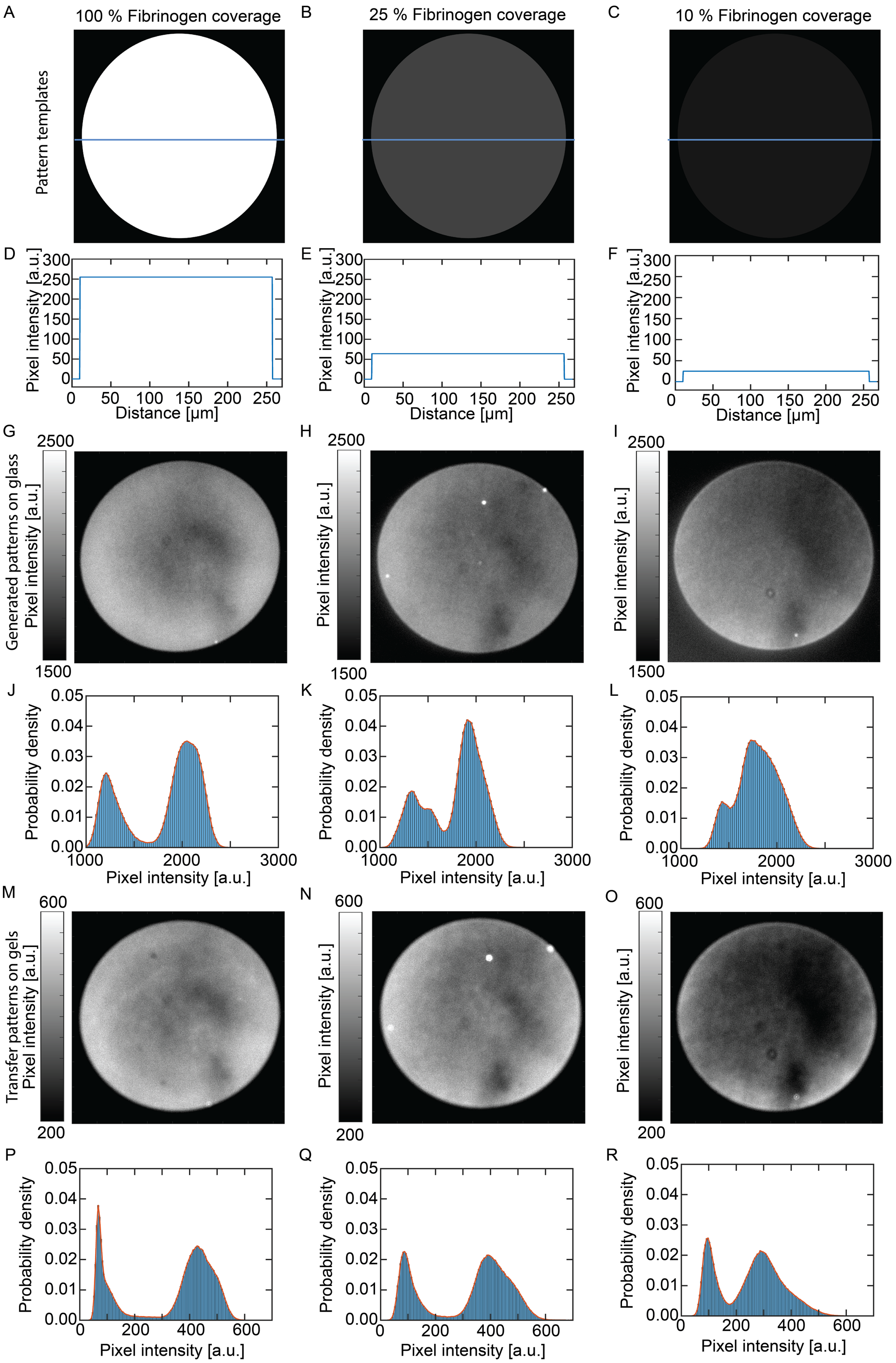

Fig. S2 (A, B, C) Template patterns in gray scale. The blue lines mark the line scans shown in (D, E, F). (G, H, I) Corresponding circular patterns with a diameter of 250 μm generated using LIMAP on glass after incubation with 100 μg/mL fluorescently labeled fibrinogen using a laser power of 2000 mJ/mm^2. (J, K, L) Corresponding histograms. (M, N, O) Circular patterns from (G, H, I) after transfer to the gels. (P, Q, R) Corresponding histograms.

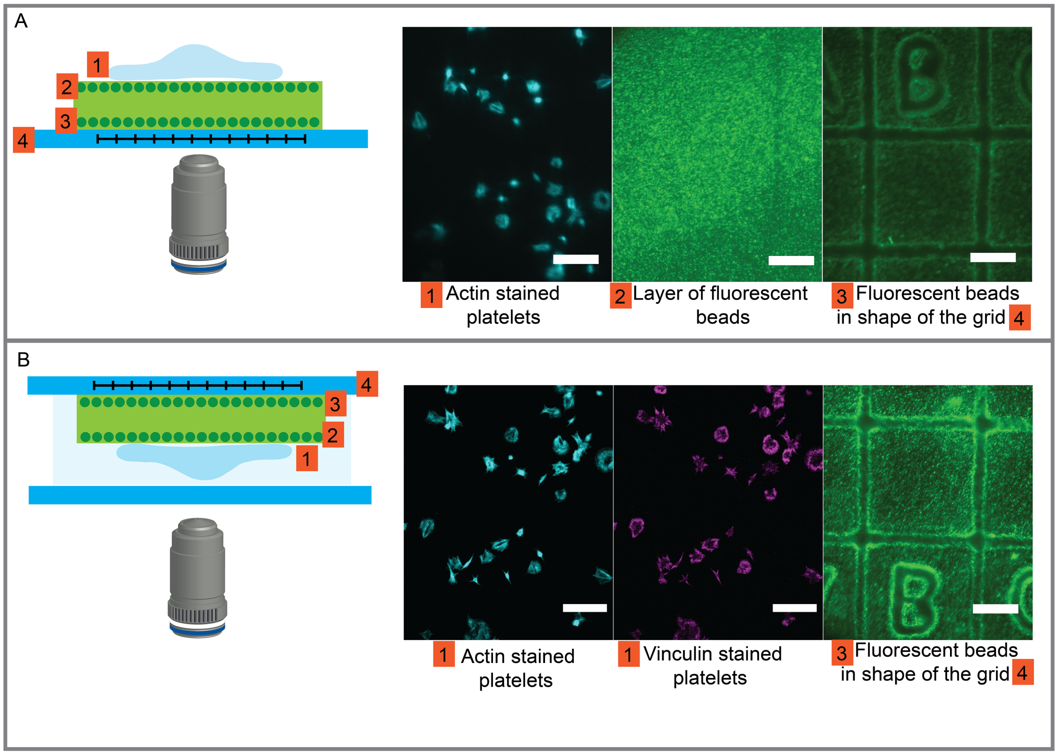

Fig. S3 Schematic of the procedure taken for superresolution imaging of platelets. A) Step one: life cell imaging and simultaneous TFM through the thick PAA gel with embedded fluorescent beads. (1) Shows an example image of actin structures (SiR-actin) in the platelets; (2) shows the bead layer just underneath the spreading platelet; (3) shows the grid pattern on the glass slide (4), which is used for relocalization of the same platelet. B) Step two: STED imaging of fixed platelets. (1) Shows the same actin structures as in (A), now at high resolution, and the corresponding vinculin structures (focal adhesions); (3) the beads take on the grid pattern and can be used for relocating the same cells. Scale bars correspond to 20 μm.

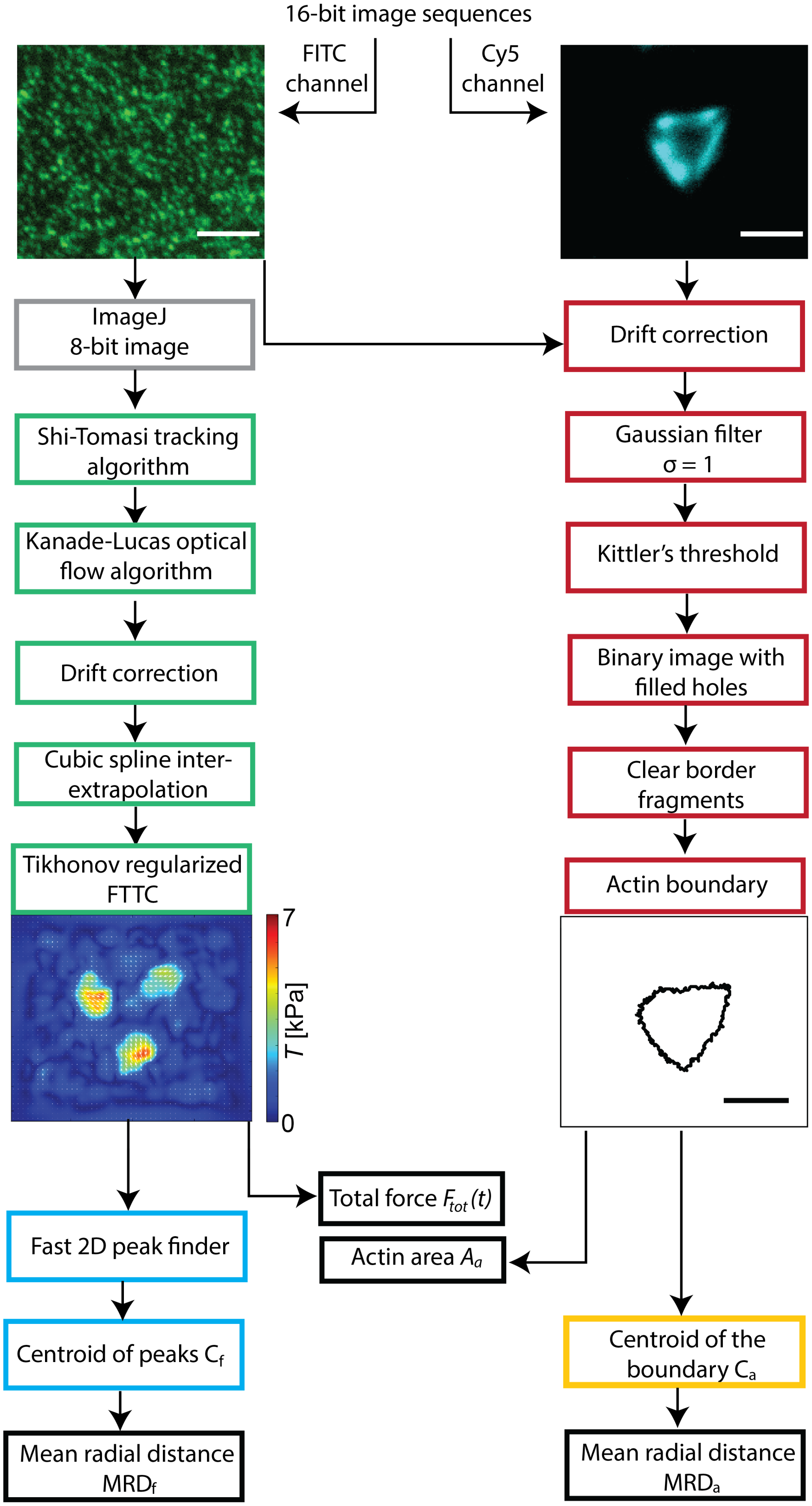

Fig. S4 Scheme of algorithms used for the analysis for traction force and actin area computation. The colors describe the classification of each step: green boxes show the individual steps in the algorithm for regularization-based approach of TFM, gray box shows input data for TFM, red boxes show the actin area determination, the yellow box shows the determination of the mean radial distance of the actin area MRD_a, blue boxes show the determination of the mean radial distance of the force MRD_f and black boxes show the data outputs. Scale bars correspond to 5 μm

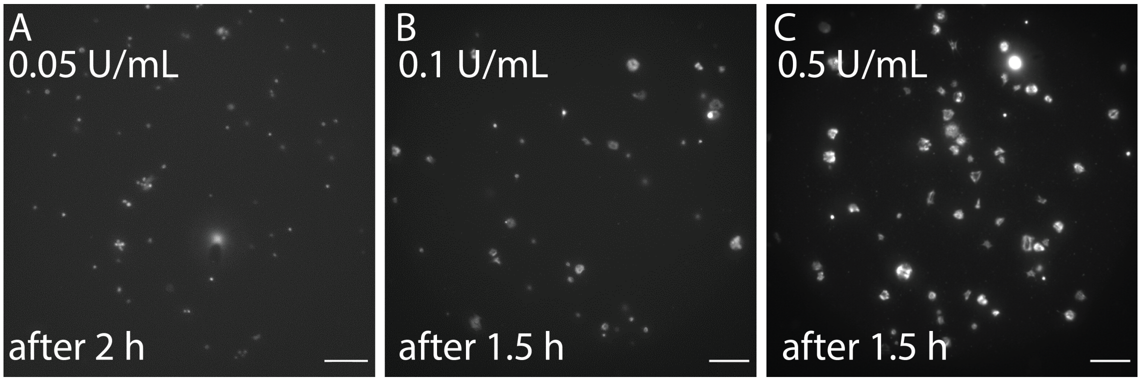

Fig. S5 Examples of activated platelets on gels labeled with SiR-actin after a minimum of 1.5 hours. A) Platelets activated by 0.05 U/mL thrombin. B) Platelets activated by 0.1 U/mL thrombin. C) Platelets activated by 0.5 U/mL thrombin. Scale bars correspond to 20 μm.

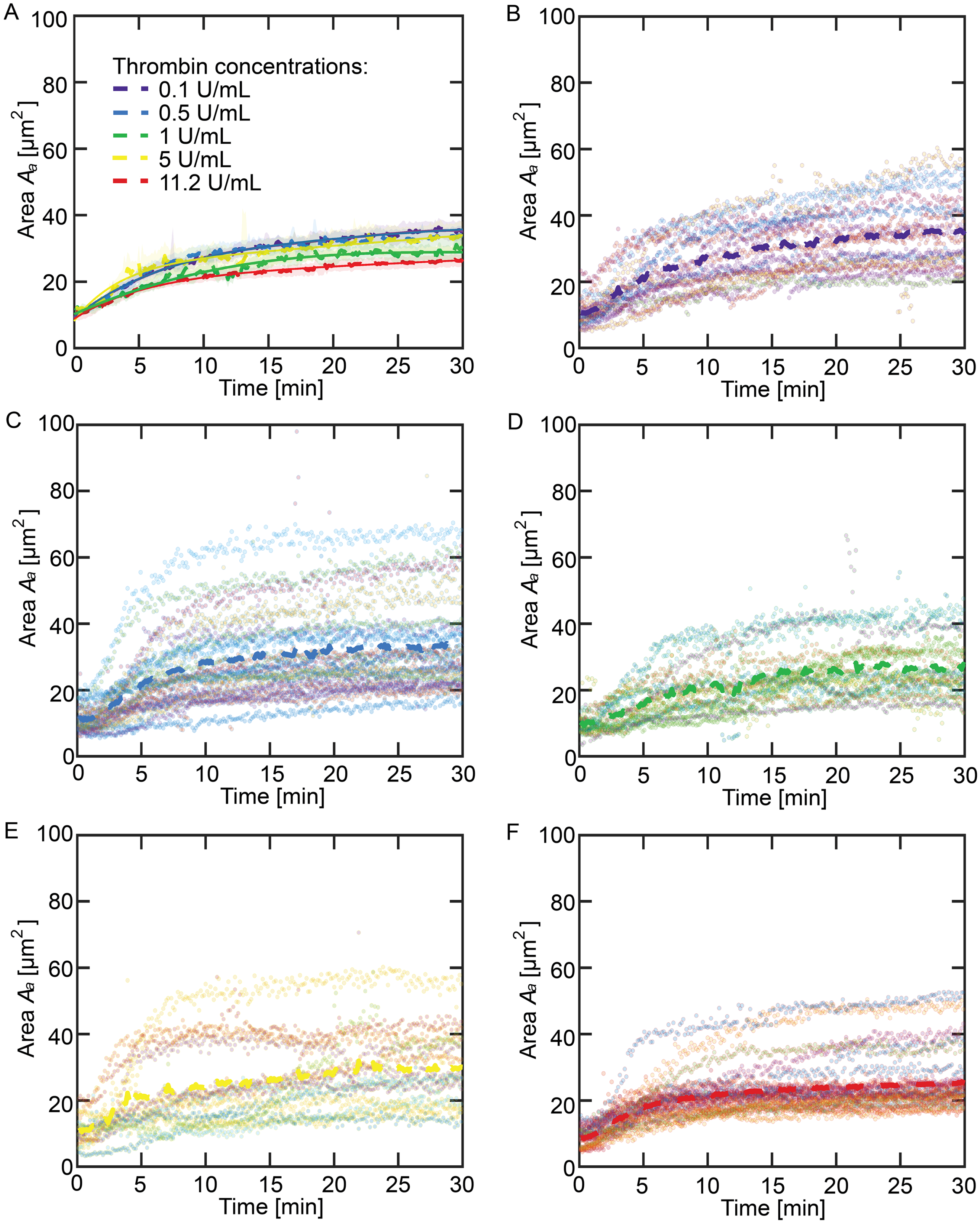

Fig. S6 The average time-dependent total area covered by actin structures $A_a(t)$ upon stimulation by different concentrations of thrombin. (A) Same plot as shown in Fig. 1C in the main text. Individual data curves for (B) 0.1 u/mL, (C) 0.5 U/mL, (D) 1 U/mL, (E) 5 U/mL, (F) 11.2 U/mL.

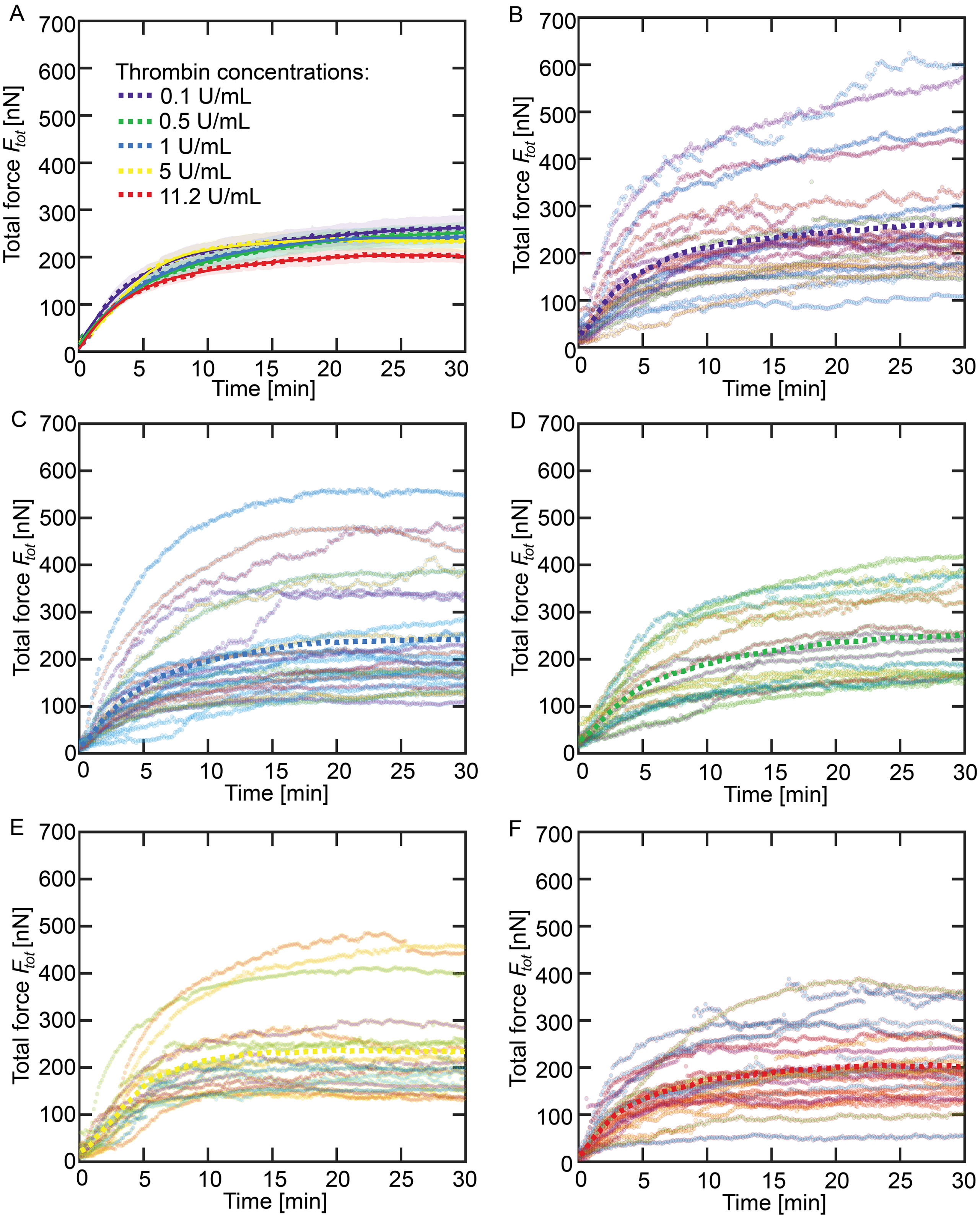

Fig. S7 Average total force curves for blood platelets upon stimulation by different concentrations of thrombin. (A) Same plot as shown in Fig. 1F in the main text. Individual data curves for (B) 0.1 u/mL, (C) 0.5 U/mL, (D) 1 U/mL, (E) 5 U/mL, (F) 11.2 U/mL.

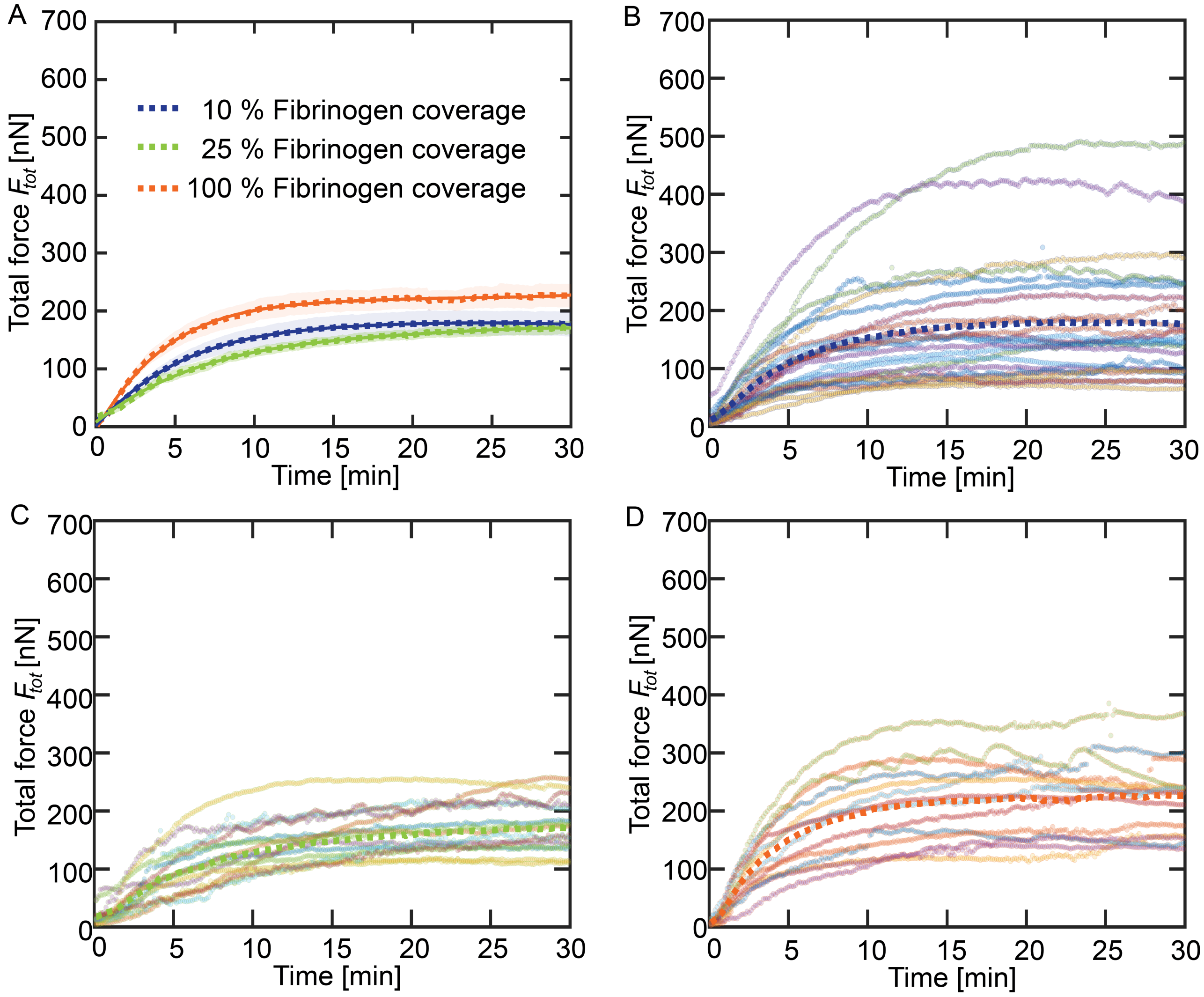

Fig. S8 Average total force curves for blood platelets upon stimulation by thrombin (0.1 U/ml) on gels covered by various concentrations of fibrinogen. (A) Same plot as shown in Fig. 2B in the main text. Individual data curves for (B) 10%, (C) 25%, (D) 100% fibrinogen coverage.

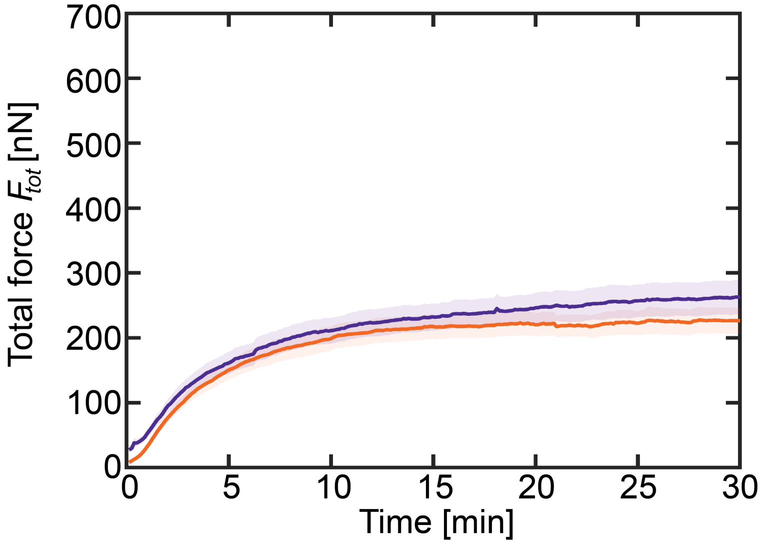

Fig. S9 Average total force curves for blood platelets upon stimulation by 0.1 U/mL thrombin on fibrinogen-coated gels obtained either by photo patterning (LIMAP) (orange line, the standard error is included as the orange transparent area) or by regular coating (blue line, the standard error is included as the blue transparent area).

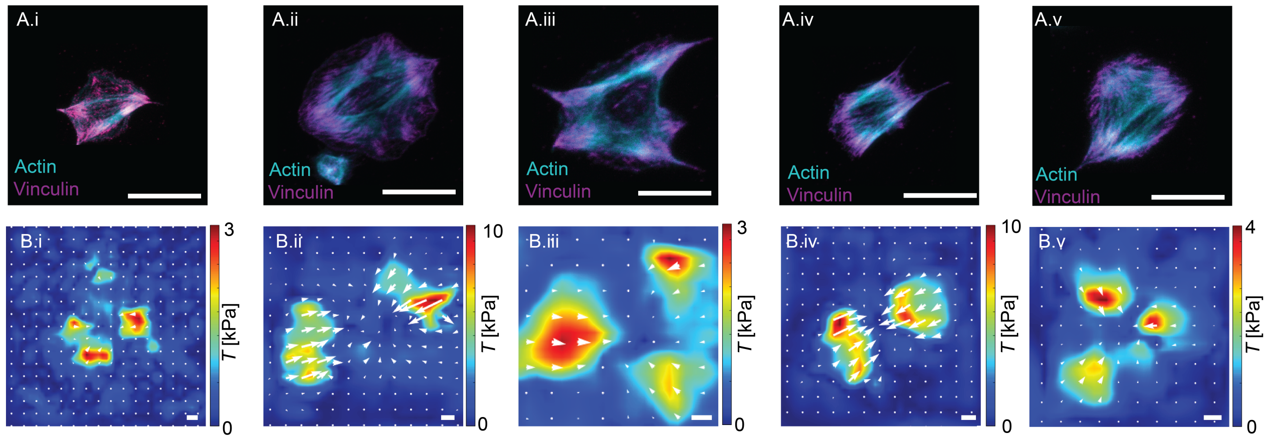

Fig. S10 STED-imaging of fixed and stained cells reveals details of cell organization during force generation. (A) Superresolution STED-imaging; actin in cyan and vinculin in magenta; the white scale bars correspond to 5 μm. (B) Traction forces before fixation with the white arrows indicating the direction of the force fields; the white scale bars correspond to 5 kPa.

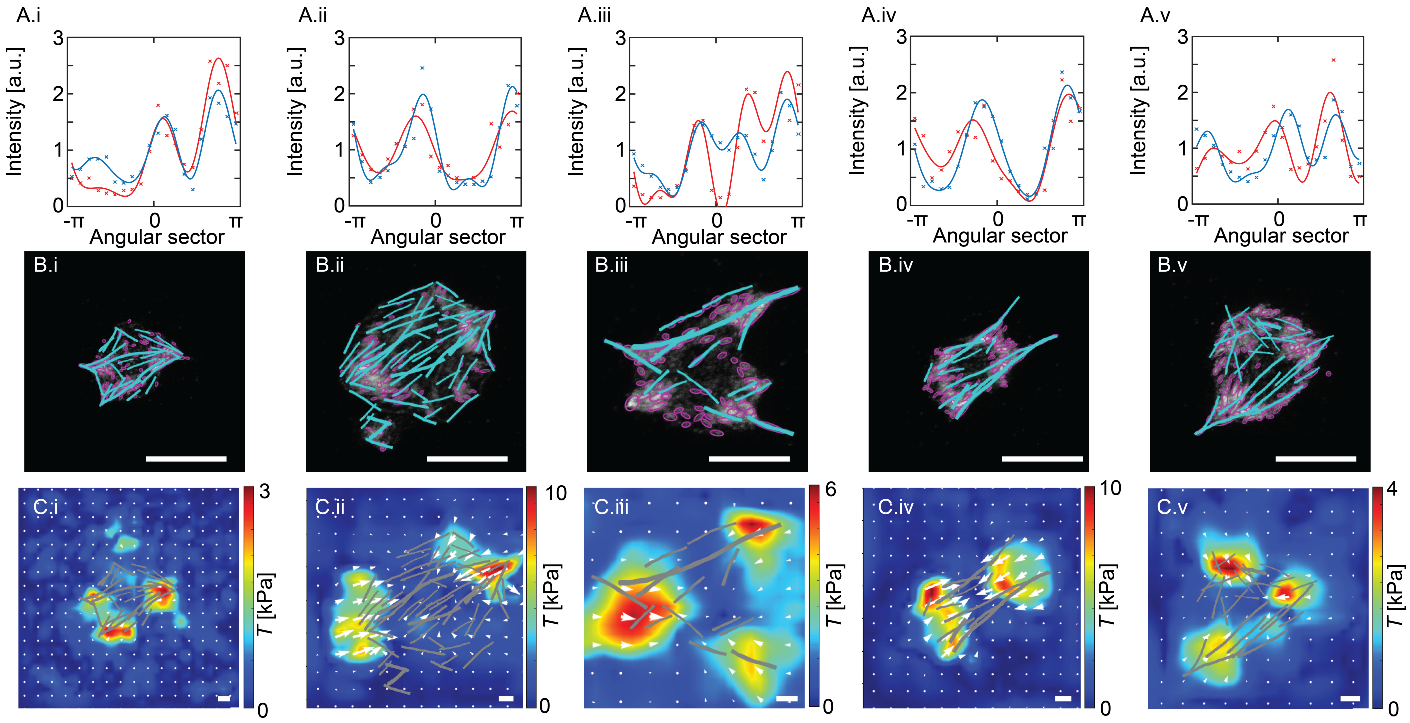

Fig. S11 Actin structures, vinculin patterns and force fields are correlated. The same cells (i) to (v) as in Fig. S10 are shown. (A) Circumferential profiles of the vinculin signal (red) and force magnitude (blue); the crosses show the data, the solid lines are fits. (B) Overlay of segmented vinculin spots (focal adhesions; magenta) and stress fibers (cyan); the white scale bars correspond to 5 μm. (C) Overlay of the traction forces field and the segmented stress fibers (gray); the white scale bars correspond to 5 kPa.

Captions of supplementary movies

Movie S1 Typical example for the development of the actin structure in a spreading platelet; 7.5 s per frame. Note that the contrast for each frame has been adjusted based on the intensity histogram for better optical visualization of early time points. Scale bar corresponds to 5 μm.

Movie S2 Temporal development of the traction force map, corresponding to Movie S1; with the detected boundary of the actin area indicated as black lines. The centroid is represented by a blue star. Magenta crosses represent the centers of the force hotspots and white arrows indicate the direction of the force field. For each image, the centroid of the hotspots is calculated and is represented as an orange cross. Scale bar corresponds to 5 μm.

Movie S3 Temporal development of the fluorescent bead positions used for calculation of traction force map. The data correspond to Movies S1 and S2. Scale bar corresponds to 5 μm.

Determination of adhesion sizes and orientations from the vinculin data

In order to extract the position of each adhesion, we first apply a 2nd order Sobel filter to compute the Laplacian. This identifies the points where the intensity is maximal. We apply a threshold to create a bitmap around these points. We then perform a connected component analysis and only retain sufficently large patches. For each component i, the orientation ϕ_i, the length a_i of its semi-mayor axis and its excentricity ε_i can be calculated using a moment analysis. We calculate the center of the adhesion $(x_0, y_0)^T$ as

$$\begin{pmatrix} x_0 \\ y_0 \end{pmatrix} = \int_{\Omega_i} \begin{pmatrix} x \\ y \end{pmatrix} \mathrm{d}x\,\mathrm{d}y,$$

where Ω_i is the area of the patch found in the component analysis. We then find the centered moment matrix M_i by

$$M_i = \int_{\Omega_i} \begin{pmatrix} (x-x_0)^2 & (x-x_0)(y-y_0) \\ (x-x_0)(y-y_0) & (y-y_0)^2 \end{pmatrix},$$

using summation to numerically solve the integral. If the adhesion was an ellipse, we would have for the moment matrix

$$M_{el} = \alpha a^4 \sqrt{1+\varepsilon^2} \begin{pmatrix} 1+\varepsilon^2 \sin^2(\phi) & \varepsilon^2 \cos(\phi)\sin(\phi) \\ \varepsilon^2 \cos(\phi)\sin(\phi) & 1+\varepsilon^2 \cos^2(\phi) \end{pmatrix},$$

where the factor α takes into account that the threshold will only retain the innermost parts of the adhesion. By comparing both expressions for the moment matrix, we find that

$$\begin{aligned}
\phi &= \frac{1}{2} \operatorname{arctan2}(2M_{i,xy}, M_{i,xx} - M_{i,yy}) \\
S &= \alpha a^4 \sqrt{1+\varepsilon^2}(2+\varepsilon^2) = M_{i,xx} + M_{i,yy} \\
T &= \alpha a^4 \sqrt{1+\varepsilon^2}\varepsilon^2 = \sqrt{(M_{i,xx} - M_{i,yy})^2 + 4 * M_{i,xy}} \\
\varepsilon &= \sqrt{\frac{2T}{S-T}} \\
a &= \left(\frac{2T}{\alpha\sqrt{1+\varepsilon^2}\varepsilon^2} \right)^{1/4}.
\end{aligned}$$

The $\operatorname{arctan2}$ function follows the usual convention, such that $\operatorname{arctan2}(y,x) = \arctan(y/x)$ for positiv y and x values. In our analysis, we empirically selected a threshold value of -2.5, retained components comprimising more them 25 pixels and used a scaling factor $\alpha = 3^{-4}$.
